# Uncovering misperceptions of social inequalities: what matters most, objective class or subjective social status?

**DOI:** 10.3389/fsoc.2025.1617413

**Published:** 2025-11-07

**Authors:** Giacomo Melli, Leo Azzollini

**Affiliations:** 1 Department of Sociology, University of Oxford, Oxford, United Kingdom; 2 Center for Social Inequality Studies & Life Course Longitudinal Laboratory, Department of Sociology and Social Research, University of Trento, Trento, Italy

**Keywords:** social stratification, social inequality, perceptions of inequality, social class, subjective social status, perceived inequality structure

## Abstract

Perceptions of social inequality are shaped not only by individuals’ objective social class but also, and more powerfully, by their subjective social status. Drawing on data from the International Social Survey Programme (ISSP) covering 35 countries and 96 country-years between 1992 and 2019, this study disentangles the distinct and interactive effects of class and subjective status on how people perceive social inequality. While individuals in lower objective classes are somewhat more likely to perceive society as unequal, this effect diminishes once subjective social status is considered. Subjective status proves to be a significantly stronger predictor: individuals who perceive themselves on the lower rungs of society consistently perceive social structures as being highly unequal. When class and status align, their effects on perceived inequality reinforce each other; when they diverge, subjective status predominates. This highlights the significance of integrating subjective dimensions into the study of social stratification. These findings contribute to a growing literature emphasizing the sociopolitical relevance of subjective evaluations of social position, and show that considering class and status together provides a more comprehensive understanding of how inequality is perceived.

## 1 Introduction

How do objective social class and subjective social status shape individuals’ perceptions of inequality in contemporary societies? Earlier social-scientific research primarily focused on objective social stratification characteristics such as social class (Lipset and Rokkan, 1967) or income (Meltzer and Richard, 1981), and on their influence on attitudes towards redistribution, which, in turn, have, shaped the political coalitions and policy choices that underpin welfare state development (Esping-Andersen, 1990). More recent contributions have emphasized the moderating and mediating dynamics of this relationship (Carriero, 2016; Schmidt-Catran, 2016; Fernández and Jaime-Castillo, 2018). At the same time, a growing body of research has investigated the political implications of *social status*, understood as the second component of stratification in the classic ‘class-status-parties’ Weberian framework (Weber, 1968 [1922]; Gidron and Hall, 2017, Nolan and Weisstanner, 2022; Oesch and Vigna, 2023; Melli, 2025). This research stream also examines its impact on attitudes towards redistribution, showing that it operates alongside the influence of social class (Brown-Iannuzzi et al., 2015; Bobzien, 2020; Kalleitner and Bobzien, 2024; Melli and Azzollini, 2025).

Both social class and social status are central to understanding stratification and political dynamics. Weber (1968 [1922]) characterized them as partially overlapping yet analytically distinct dimensions. Building on this tradition, the present study focuses on subjective social status, measured through self-placement on the social ladder (Adler et al., 2000). Subjective social position partially overlaps with but does not replicate Weber’s conception of status. It captures a related dimension of stratification that is highly relevant to individuals’ perceptions of inequality. This measure has gained wide application across countries owing to its straightforward reference to the top and bottom of the social hierarchy, which enables robust cross-national comparisons (Evans and Kelley, 2017; Raudenská, 2024).

Despite its conceptual relevance, the perspective of subjective social status has been only sparsely applied to the study of individuals’ *subjective* perception of inequality, an important antecedent of attitudes towards redistribution. A growing body of research indicates that perceptions of inequality can considerably influence socio-political outcomes (Andersen and Curtis, 2015; Haddon and Wu, 2022), yet these perceptions are only weakly related to *objective* levels of economic inequality (Kenworthy and Owens, 2011; Brooks and Manza, 2013). Prior work has mainly examined how perceptions of inequality are influenced by objective characteristics such as class (Haddon and Wu, 2022), whereas research incorporating subjective social status remains limited (Hajdu, 2024).

This study contributes to this emerging research stream in three ways. First, it integrates and contrasts theoretically the perspectives of objective social class and subjective social status, providing a unified framework for analysing how people perceive inequality in society. Second, it empirically disentangles their independent associations with perceptions of social inequality, by establishing and assessing their substantive influences as well as allowing systematic comparison between them. Third, it examines their joint effects by analysing mismatches between the two, asking whether individuals in the same objective class but with different subjective status perceive inequality differently, and vice versa.

To address these questions, the analysis relies on the International Social Survey Programme’s Social Inequality Modules (1992, 1999, 2009, and 2019), which cover 35 countries and 96 country-waves. This dataset provides one of the broadest comparative bases for studying inequality perceptions and is widely relied upon in social inequality research (see Hadler and Neumayr, 2023; Roberts et al., 2023; for more details on the dataset and its social inequality findings). Generalized Ordinal Logistic regression models with socio-demographic controls and country-wave fixed effects are employed to isolate the statistical effects of social class and subjective status. The findings indicate that subjective social status exerts a stronger and more consistent influence on perceptions of inequality than objective class, although its relative importance varies across class positions.

## 2 Theory and hypotheses

Before engaging with the description of the data and methods employed in this study, we briefly outline the theoretical framework related to perceptions of inequality, followed by a discussion of how these perceptions are influenced by social class and subjective status separately, before theorizing about their joint impact on the outcome of interest.

While the dangers posed by actual economic inequalities have long been understood (Piketty, 2014; Atkinson, 2015; Pickett and Wilkinson, 2015), subjective perceptions of inequality are increasingly recognized as equally, if not more, problematic for democratic societies (Andersen and Curtis, 2015). An extensive body of research shows that citizens often misperceive the extent of inequality (Neckerman and Torche, 2007; Mijs, 2021) and that the relationship between actual and perceived levels of inequality is weak (Kenworthy and Owens, 2011; Brooks and Manza, 2013; Margalit, 2013). Current research (Wiesner, 2025) provides further insight into this relationship, highlighting that citizens tend to better grasp trends in inequality, especially rising inequality, but struggle to compare inequality across countries. These misperceptions have political consequences: when inequality is not perceived as severe, citizens may be less likely to support redistributive policies (Mijs, 2021), helping to explain how economic inequalities persist even in societies with universal suffrage (Schröder and Neumayr, 2023).

Theoretically, *perceived* inequalities are considered as a stronger predictor of attitudes towards redistribution than *actual* inequalities (Bobzien, 2020), because mechanisms such as self-interest operate through what individuals *believe to be true* rather than through objective levels of inequality (Bobzien, 2020).

Thus, a better understanding of how individuals form their own perceptions of inequality, and how this process is related to dimensions of social stratification, can provide greater insights into the possible pathways to mitigate the objective levels of economic inequality. Reflecting the importance of this social phenomenon, there is increased attention on *how to measure* such perceptions of inequality. A mainstay in this stream of research focuses on whether respondents agree with the statement that ‘income differences in the country are too large’ (Roberts et al., 2023). Yet this item captures not only perceived extent but also judgments about legitimacy. A more profound and broad understanding comes from questions dealing with the perceived *shape* of inequality in society (Evans et al., 1992; Evans and Tilley, 2017; Hadler and Neumayr, 2023), which form the basis of our analysis. The absence of legitimacy judgements in the latter item constitutes both an advantage and a disadvantage: on one hand, it does not prime the respondents towards perceiving inequalities as unjust, but on the other, individuals who perceive societal structures as unequal may consider the latter as justified on the grounds of meritocracy. To address this issue, we replicate our analyses with the more conventional ‘income differences are too large’ item (Supplementary Tables A9–A11 and Supplementary Figures A2, A3).

Among the stratification dimensions affecting the perceptions of inequality, social class has received the most attention. Class is a foundational concept that captures economic power (Weber, 1968 [1922]) and positions individuals within wider occupational structures (Blau and Duncan, 1967). Beyond occupation, social class also accounts for pay type, the degree of independence in work tasks, and supervisory responsibility (Erikson et al., 1979; Evans and Mills, 1998; Rose and Harrison, 2007), as well as different work logics (Oesch, 2006). A vast literature shows its influence on socio-political outcomes (Lipset and Rokkan, 1967; Korpi, 2018 [1984]; Hout et al., 1995; Evans, 1999; Evans and Tilley, 2017), and its importance for perceptions of inequality has also been studied, albeit relatively less than for attitudes towards redistribution (Haddon and Wu, 2022). For redistributive attitudes, the classic finding is that individuals in disadvantaged classes support redistribution more strongly, as they have more to gain from it (Edlund and Lindh, 2015; Fernández and Jaime-Castillo, 2018), with a symmetric and opposite pattern holding for those in more advantaged social classes.

The logic developed to explain attitudes toward redistribution also provides a useful framework for understanding perceptions of inequality, which are widely regarded as a key antecedent of redistributive preferences (see Gimpelson and Treisman, 2018; Bobzien, 2020). Research shows that the *perceived* levels of inequality empirically matter *more* than the *objective* levels in shaping social policy preferences (Engelhardt and Wagener, 2014), including attitudes towards redistribution (Bobzien, 2020). Indeed, being unable to accurately assess the objective levels of economic inequality is associated with lower-than-expected levels of support for redistribution (Mijs, 2021). Thus, individuals in less advantaged social classes are more likely to perceive inequalities as more pronounced, since they are objectively situated in disadvantaged positions in the class structure. On the other hand, those in more advantaged social classes are less exposed to the adverse effects of inequality and more likely to envisage society as meritocratic (Mijs, 2021), thereby perceiving the distribution of resources as less unequal. Haddon and Wu (2022) confirm this expectation using ISSP data across several country-years: they show that working class members are considerably more likely to perceive inequality as stronger, while salariat members show the opposite pattern. Therefore, we posit that:

*Hypothesis 1:* Individuals from less advantaged objective social classes will be more likely to perceive social inequalities as more pronounced than those from more advantaged objective social classes.

While the role of objective social class is well established, attention to subjective social status as a predictor of perceptions of inequality is relatively recent (Hajdu, 2024). Subjective social status refers to the position within the social hierarchy that is perceived by individuals. Although both class and status have long been acknowledged in stratification research, as both concepts had footing in the Weberian three-component theory of stratification (Weber, 1968 [1922]), empirical work has traditionally emphasized class (Lipset and Rokkan, 1967), leaving status comparatively underexplored.

Recent work has shifted focus towards subjective social status, typically measured by asking individuals to place themselves on a ten-point ladder representing society. Studies show that subjective social status is a powerful predictor of political and social outcomes, including the rise of populism and radical right-wing parties (Gidron and Hall, 2017; Chan et al., 2020; Bolet, 2023; Melli and Scherer, 2024), as well as attitudes towards redistribution (Brown-Iannuzzi et al., 2015; Melli and Azzollini, 2025). For this latter outcome, the argument is straightforward: individuals’ preferences towards redistribution are powerfully associated with the *perceived* position within society (Bobzien, 2020; Kalleitner and Bobzien, 2024), in addition to the *objective* position within society: individuals who perceive themselves as occupying a higher position are less supportive of redistribution. A symmetric reasoning applies to individuals who perceive themselves in a ‘rung’ of society that is lower than their actual position: their preferences will also be influenced by what they believe, rather than by only what is objectively their position. Melli and Azzollini (2025) find empirical support for this argument by indicating that lower subjective social status is associated with stronger support for redistribution, even after accounting for objective variables such as social class and education.

But how should subjective social status affect the perceptions of inequality? Hajdu (2024) is one of the few articles to examine the link, albeit in reverse order: the study explores how subjective and objective economic inequality may influence the social status of an individual, with higher levels of both forms of inequality resulting in a lower subjective social status. In contrast, this study joins the growing body of research envisaging subjective social status as the *explanans*, rather than the *explanandum*, of socio-political phenomena (Gidron and Hall, 2017), and compares its role directly to the one played by social class. Nonetheless, we acknowledge that class and subjective status may be connected in a causal circle rather than in a causal chain, an issue we address empirically (Supplementary Table A8). Yet, some guidance on this issue can be found theoretically, by examining how subjective social status is determined. Subjective social status is shaped partly by objective factors such as occupation and education, but also strongly by local reference groups and social networks (Lin et al., 2001; Cansunar, 2021): individuals look at their own social network and tend to position themselves at the midpoint of this group (Bobzien, 2020; Melli, 2025). Thus, subjective status is grounded in personal and local social dynamics that may weigh more heavily on perceptions than national inequality indicators.

Thus, how can subjective social status shape perceptions of inequality in society, net of the influence of objective social class? The key mechanism here can be built from *grievance theory* (Runciman, 1966; Klandermans et al., 2008) and *self-interest* (Kraus et al., 2012; Bobzien, 2020). According to this stream of research, grievances can act as powerful catalysts of social action (Klandermans et al., 2008): dissatisfaction with one’s own socio-economic standing fuels perception of injustice and mobilization (Kern et al., 2015). In this context, individuals who feel closer to the bottom of society may view themselves as victims of distributive conflicts and express greater dissatisfaction with the socio-economic order. *In primis*, this can generate stronger perceptions of inequality: on one hand, because those near the societal bottom see more to gain from its reduction (Bobzien, 2020) and because self-interest may lead them to envisage societal inequality as harsher as a justification for their own struggles (Kraus et al., 2012).

Conversely, feeling closer to the top of society will lead to a symmetric outcome: individuals who place themselves closer to the top of society might be less likely to be dissatisfied with the broader socio-economic context and therefore less likely to perceive inequality at large. Their self-interest may further reinforce this view, as perceiving inequality as smaller legitimizes their perceived advantaged position and their role as beneficiaries of the existing distribution. Therefore, we posit that:

*Hypothesis 2:* Individuals with lower subjective social status will be more likely to perceive social inequalities as greater than those with higher subjective social status, net of objective social class.

While objective social class and subjective social status have so far been considered separately, they are closely intertwined, albeit not perfectly aligned (Melli, 2025). However, one may exert a stronger influence on inequality perceptions than the other. The next step is precisely to assess how the relationship between class and status shapes perceptions of inequality, and to determine which dimension exerts the greater influence. Subjective social status is typically influenced, but not exclusively determined, by objective social class (Melli, 2025). It reflects occupation but also concerns about education (Gidron and Hall, 2017; Chan et al., 2020), and the relative standing within one’s immediate social network (Lin et al., 2001; Bobzien, 2020; Cansunar, 2021).

Consider two examples. A lawyer in a multinational company within an urban area, and an elementary school teacher in a rural area. While the objective social classes of these two individuals differ (respectively, high-grade managers and professionals vs. low-grade managers and professionals), their networks may deeply influence their subjective social status (Lin et al., 2001): if the lawyer is consistently exposed to upper management and executives and is surrounded by individuals with tertiary degrees, their subjective social status may be affected downwards. Instead, the elementary school teacher in a rural area may perceive higher subjective status due to their social standing in the local area, as well as due to a relatively higher educational attainment compared to their network. Empirically, the two concepts overlap more often than not, but there are still considerable mismatches (Melli and Azzollini, 2025).

Having clarified the relationship between the two variables, the relative influence of class and subjective social status can be assessed through their joint association with perceptions of inequality. A useful way to illustrate this is by contrasting stylized cases. In ‘matching’ situations, where class and status coincide (high-high, or low-low), objective and subjective position reinforce each other. In ‘mismatching’ situations, however, individuals may perceive themselves in a lower or higher position than their objective class suggests. Again, *grievance theory* (Runciman, 1966; Klandermans et al., 2008) may help us. If the perceptions of inequality are influenced by dissatisfaction with one’s own socio-economic standing, *subjective* feelings about such standing may matter more than the *objective* standing: if there is a mismatch between where people *think* they are and where they *actually* are, any (dis)satisfaction may come more from the former than the latter.

Let us substantiate further this argument by examining the ‘high-class, low-status’ scenario: a person objectively belonging to a high class but perceiving themselves in a lower social position (such as the lawyer in a multinational company case before), may be dissatisfied with socio-economic affairs, and thus perceive inequalities as larger. Conversely, somebody objectively in a low class but perceiving a higher status, such as the rural teacher seen before, may reason symmetrically: by thinking they are doing relatively well socially (even if this is not the case), their socio-economic grievances may be smaller, and thus by feeling less exposed to socio-economic inequalities, they may perceive the latter as smaller. Even within the same objective class, subjective differences matter: a lawyer within a multinational company and an established lawyer with an own legal practice in a small town may occupy the same class, but if their levels of satisfaction about the socio-economic standing in society differs, they will assess themselves in different positions, and thus their perceptions of inequalities may differ despite similar occupational positions. Therefore, we posit that:

*Hypothesis 3:* When objective class and subjective social status diverge, perceptions of inequality will be shaped more strongly by subjective social status than by objective class.

*Hypothesis 4:* Within the same social class, individuals with lower (higher) subjective social status will be more likely to perceive social inequalities as stronger (weaker) than those with higher (lower) subjective status.

## 3 Materials and methods

### 3.1 Data

This study is based on data from the International Social Survey Programme (ISSP Research Group, 2024), utilizing the cumulative dataset of the Social Inequality Modules conducted in 1992, 1999, 2009, and 2019. The dataset comprises around 100,000 individuals from 35 countries: Australia, Austria, Bulgaria, Canada, Chile, Taiwan, Croatia, Cyprus, the Czech Republic, Denmark, Finland, France, Germany, Hungary, Iceland, Israel, Italy, Japan, Latvia, Lithuania, New Zealand, Norway, the Philippines, Poland, Portugal, Russia, the Slovak Republic, Slovenia, South Africa, Spain, Sweden, Switzerland, the United Kingdom, the United States, and Venezuela. In total, it encompasses 96 unique country-year cases. Sample numerosity by country and year is reported in Table 1.

**Table 1:** Sample numerosity by country and year.

Country	1992	1999	2009	2019	Total
Australia	1,500	1,274	1,101	646	4,521
Austria		786	768	848	2,402
Bulgaria	835	788	734	801	3,158
Canada	800	848			1,648
Chile		1,219	1,205	988	3,412
Croatia			878	825	1,703
Cyprus		764	826		1,590
Czech Republic	620	1,388	990	1,300	4,298
Denmark			1,172	739	1,911
Finland			677	725	1,402
France		1,553	1,996	1,102	4,651
Germany		1,057	959	935	2,951
Great Britain			724	1,096	1,820
Hungary	997	897	837		2,731
Iceland			777	897	1,674
Israel		1,066	959	1,027	3,052
Italy	881		829	860	2,570
Japan		980	896	848	2,724
Latvia		969	882		1,851
Lithuania			704	765	1,469
New Zealand	965	885	710	742	3,302
Norway	1,181	1,031	1,025	992	4,229
Philippines	1,113	1,086	1,044	3,753	6,996
Poland	1,236	806	1,020		3,062
Portugal		982	673		1,655
Russia		1,184	1,139	1,342	3,665
Slovak Republic	391	946	925		2,262
Slovenia		749	747	813	2,309
South Africa			2,892	2,015	4,907
Spain		895	890		1,785
Sweden		920	885	1,114	2,919
Switzerland			929	2,249	3,178
Taiwan			1,747	1,508	3,255
United States		900	937	1,196	3,033
Venezuela			861	1,048	1,909
Total	10,519	23,973	34,338	31,174	100,004

Data from ISSP Research Group (2024), unweighted.

### 3.2 Variables

**Figure 1:**
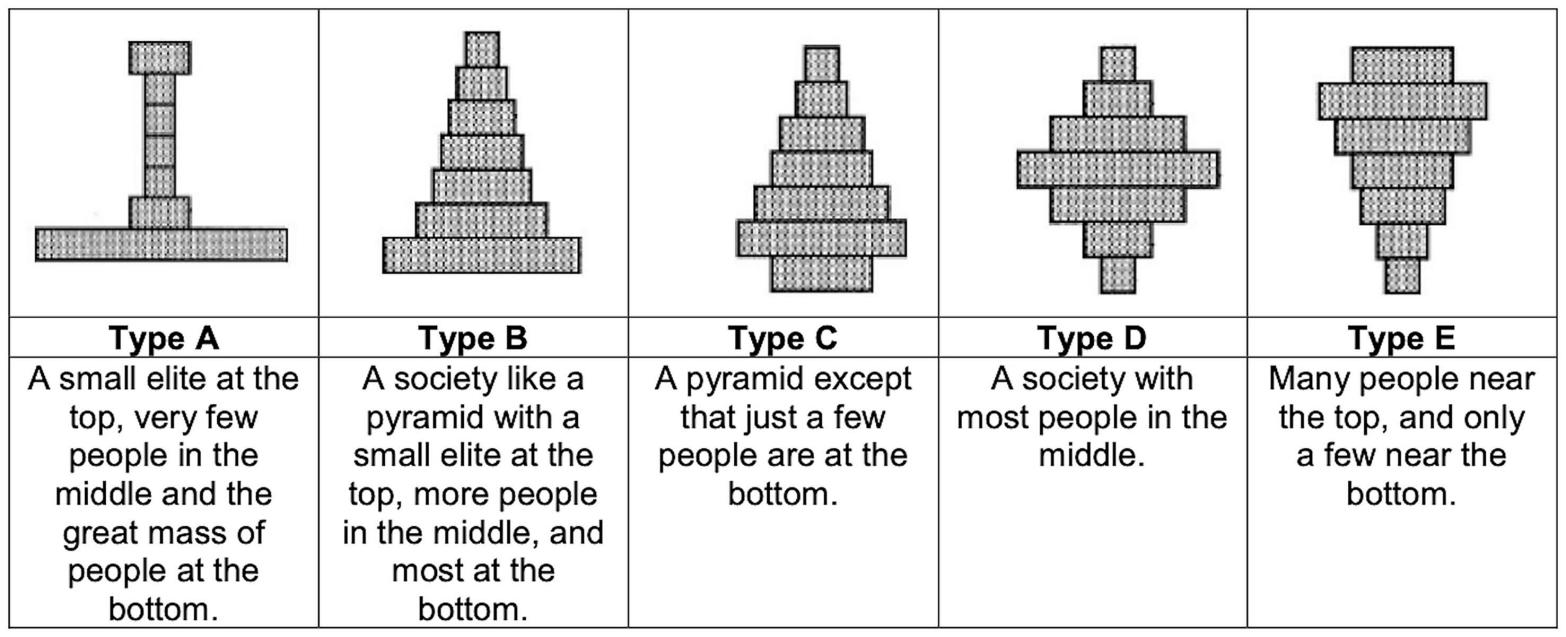
Question on perceived structure of social inequality. Respondents select one of five diagrams representing different societal structures: Type A shows a highly unequal society with many at the bottom; Types B and C reflect pyramid shapes with moderate inequality; Type D depicts most people in the middle; and Type E shows a highly equal society with most near the top. This measure captures perceptions of the shape of inequality without normative wording. Source: ISSP Research Group (2024).

Perceptions of inequality can be measured in various ways. In this study, we focus on perceptions of the *shape* of the social structure of inequality, or perceived social structure for brevity. This is captured by a question in all ISSP Social Inequality questionnaires that asks respondents to choose one of five diagrams representing the type of social structure they believe exists in their country (see Figure 1). While the question presents five discrete options, previous research has consistently treated them as reflecting an underlying continuum of inequality (Evans et al., 1992; Evans and Kelley, 2017; Gimpelson and Treisman, 2018; Hadler and Neumayr, 2023), an approach we follow here. Type A is typically interpreted as representing a highly unequal society, with many individuals at the very bottom, fewer in the middle, and some at the top. Types B and C reflect moderate levels of inequality, with a ‘pyramid’ shape characterized by a large base but also a sizeable middle. Types D and E suggest more equal structures: one with a dominant middle group and another with most individuals near the top and only a few below. The visual format is particularly valuable, as it prompts respondents to consider the overall shape of social stratification without invoking normative terms, thus capturing a crucial yet underexamined dimension of inequality perception.

An alternative measure included in the ISSP Social Inequality questionnaire captures perceptions of income inequality specifically, rather than the broader structure of social inequality. While useful and important, it reflects a different conceptual focus. Our interest lies in how individuals perceive the overall shape of stratification in society. Nonetheless, we include this alternative indicator in robustness checks (Supplementary Tables A11, A19), which yield results consistent with our main findings.

Subjective social status is measured with a widely used item in the literature (Gidron and Hall, 2017; Melli and Azzollini, 2025; Nolan and Weisstanner, 2022; Vigna, 2023), which asks respondents to place themselves on a ten-point ladder representing society. Because no explicit reference points are given, the item is well suited for cross-national comparisons (Evans and Kelley, 2004; Kelley and Evans, 1995), and its validity has been confirmed in recent studies (Raudenská, 2024). In all survey waves, this question was asked before the item on perceived social structure. Thus, the ordering of the survey items prompts respondents to first reflect on their subjective social status and only later to report their perceived social structure (the dependent variable). Research on question-order effects in survey research shows that earlier items can frame responses to subsequent ones (see Schuman and Ludwig, 1983; Stark et al., 2020). This sequencing is consistent with our modelling strategy, partially mitigating issues of reverse causality.

Social class is measured using the five-class version of the European Socio-Economic Classification (Rose and Harrison, 2007), which is derived from the Erikson-Goldthorpe-Portocarero (EGP) class schema (Erikson et al., 1979; Evans and Mills, 1998), itself grounded in Max Weber’s theory of class, status, and party (Weber, 1968 [1922]). This classification is operationalized on the basis of individuals’ employment status and occupational characteristics, grouping individuals who occupy similar positions within the labour market. The five-class version distinguishes between higher-grade managers and professionals, lower-grade managers and professionals, those in intermediate occupations (such as office clerks), the self-employed and small business owners, and the working class. These categories reflect differences in economic conditions that influence a range of outcomes, including income and the risk of unemployment (Chan and Goldthorpe, 2007). Social class, in this sense, captures the relative advantages and disadvantages associated with one’s objective position in the social hierarchy, and serves as a valuable tool for analyzing variation in material conditions and economic interests.

The final models also include a set of control variables that may influence individuals’ perceptions of the societal structure. These include household income (measured in country-year-specific terciles^1^), the highest level of educational attainment, the social class of origin (measured as the higher class of the two parents), age, gender, and year of data collection. For household income, education, social class, and class background, a separate category is included to account for missing information. Descriptive statistics are presented in Table 2.

**Table 2:** Descriptive statistics.

Variables	Mean/%	Std dev	Min	Max
Perceived social inequality structure				
A	25.78			
B	31.31			
C	19.59			
D	20.15			
E	3.17			
Subjective social status				
1—Lowest	3.66			
2	4.53			
3	10.05			
4	14.01			
5	22.76			
6	23.20			
7	13.32			
8	6.36			
9	1.29			
10—Highest	0.82			
Social class				
Higher-grade managers and professionals	9.19			
Lower-grade managers and professionals	16.12			
Intermediate occupations	17.70			
Self-employed and small employers	9.33			
Working class	27.05			
NA	20.61			
Household income				
Low	23.08			
Middle	27.63			
High	31.97			
NA	17.32			
Education				
Primary	30.53			
Secondary	42.98			
Tertiary	22.32			
NA	4.17			
Social class of origin				
Higher-grade managers and professionals	8.23			
Lower-grade managers and professionals	10.79			
Intermediate occupations	14.38			
Self-employed and small employers	16.16			
Working class	27.44			
NA	23.00			
Age	40.80	13.43	18	65
Female	51%		0	1
Wave				
1992	10.35			
1999	23.71			
2009	34.30			
2019	31.64			

Data from ISSP Research Group (2024), weighted. *N* = 100,004.

### 3.3 Analytical strategy

Because the dependent variable consists of five ordered categories, an ordered logit model was initially considered as part of selecting the most appropriate analytical strategy. This model rests on the proportional odds assumption, which requires the effects of predictors to remain constant across all cumulative splits of the outcome (e.g., comparing category 1 vs. 2–5, or categories 1–2 vs. 3–5). Formal tests showed that this proportional odds assumption was violated in our data.^2^ A multinomial logit model was also considered; however, it treats outcome categories as nominal and ignores their natural ordering. In addition, it relies on the Independence of Irrelevant Alternatives assumption, which posits that the relative odds between any two categories are unaffected by the presence or characteristics of other alternatives, a condition that does not hold in this case.^3^

For these reasons, Generalized Ordered Logit models (Williams, 2006, 2016) were employed. This specification selectively relaxes the proportional odds assumption, allowing coefficients to vary across outcome thresholds where necessary, while constraining them where the assumption is met. Model fit comparisons, including the Bayesian Information Criterion (BIC; Raftery, 1995), further support the adoption of this modelling strategy.

All models include country-wave fixed effects to account for both time-invariant and time-variant heterogeneity at the national level. Survey weights are applied to adjust for the sampling design and ensure nationally representative estimates. Standard errors are clustered by country-wave to account for potential intra-group correlation.

## 4 Results

**Figure 2:**
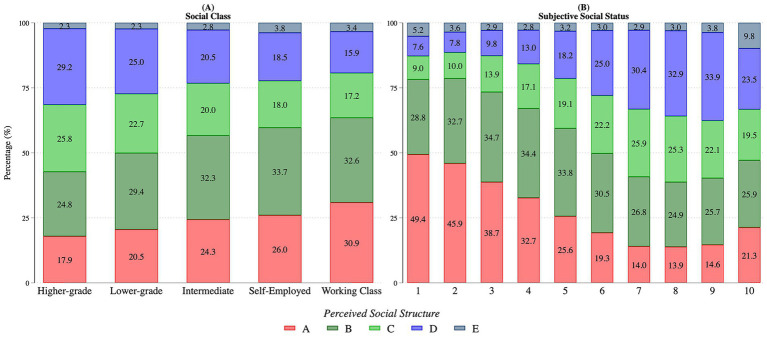
Perceived social inequality structure by social class **(A)** and subjective social status **(B)**. Stacked bars show the percentage distribution of respondents selecting each perceived inequality type (A–E, as defined in Figure 1). Panel A groups respondents by objective social class; Panel B groups them by subjective social status (1–10). Results are based on ISSP Social Inequality Modules (1992, 1999, 2009, and 2019), weighted. *N* = 100,004.

Commencing with descriptive results, Figure 2 illustrates considerable variation in individuals’ perceptions of the social structure within objective social class and subjective social status groups across the countries under study. These descriptive results indicate that most individuals perceive their society to resemble a pyramid, corresponding to Types B and C. This pattern holds across social classes, as shown in Figure 2A. Nevertheless, members of the working class are more likely to characterize their society as highly unequal, as reflected in the selection of Type A, compared to individuals from more advantaged classes. Conversely, the perception of a more equal diamond-shaped society (Type D) is more frequently expressed by those in higher-grade and lower-grade managerial and professional occupations than by working class members.

Figure 2B presents the bivariate association between perceived inequality structure and levels of subjective social status. A clear gradient emerges, as individuals who place themselves lower on the social ladder are more likely to perceive society as highly unequal (Type A, above 40% of the sample for rungs 1 and 2), while those who identify with higher social strata are more inclined to perceive their society as relatively equal. However, this association is far from perfect. Between 10 and 15 percent of respondents who rate their subjective status below 4 nonetheless describe their society as relatively equal, while more than 14% of those placing themselves at rung 7 or above still perceive society as unequal. These findings highlight not only the modest association between subjective social status and perceived inequality structure visible in Figure 2, but also suggest that the two measures capture distinct, though related, dimensions of social perception.

**Figure 3:**
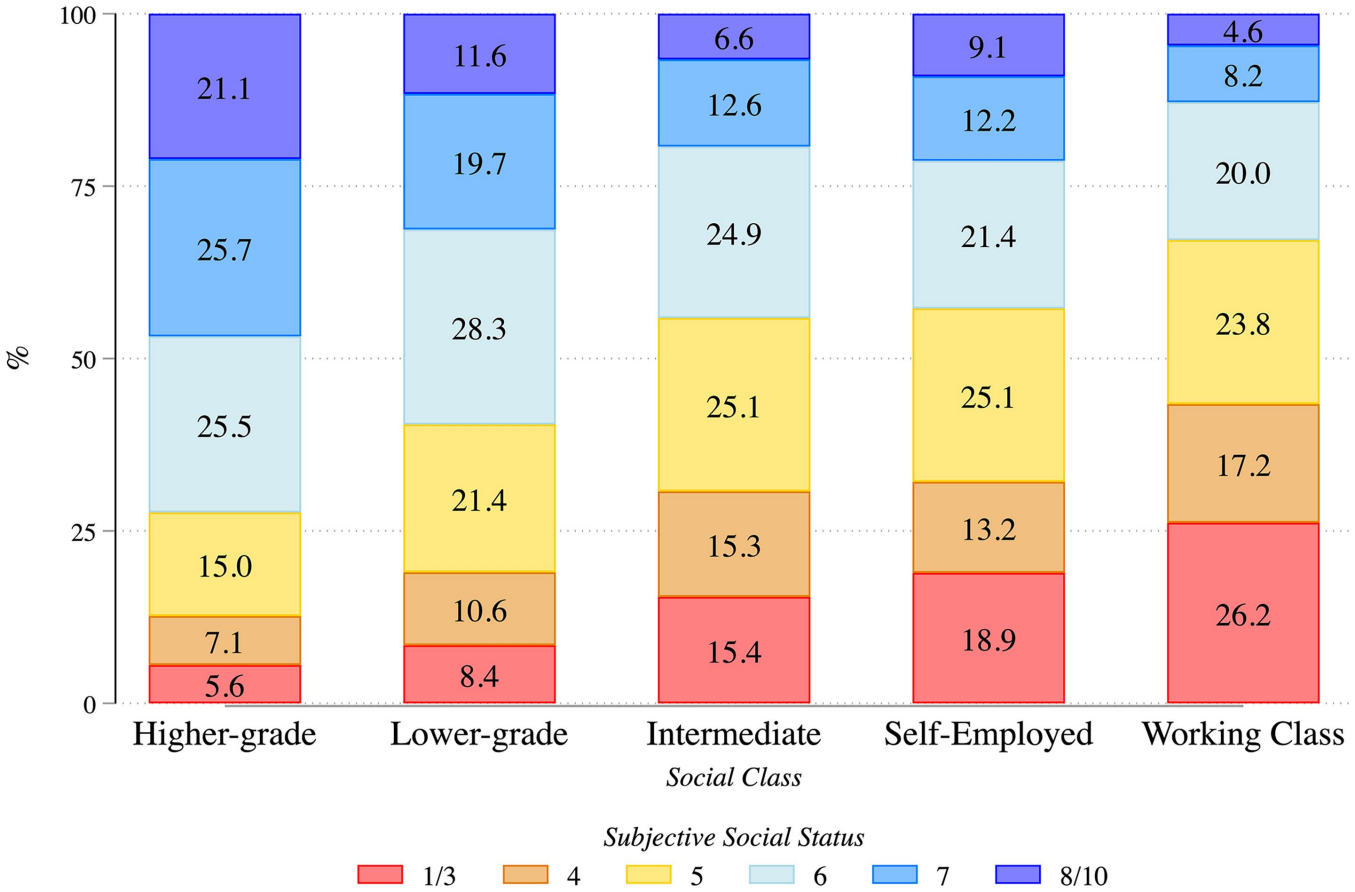
Distribution of subjective social status by social class. Stacked bars show the percentage distribution of self-reported subjective social status (1–10, collapsed into categories shown in the legend) within each objective social class. Results are based on ISSP Social Inequality Modules (1992, 1999, 2009, and 2019), weighted. *N* = 100,004.

The relationship between objective class and subjective status is also examined. Beyond the theoretical justification for a comprehensive analysis of social class and subjective social status, as discussed in the previous section, we assess empirically the latter by reporting the variation of subjective social status within objective social classes. As illustrated in Figure 3, while there is a general tendency for members of more advantaged social classes to position themselves subjectively in the higher strata of society (21% of the higher-grade salariat position report a subjective position of 8 and above), this association is far from perfect. Approximately 28% of individuals in the higher-grade managers and professionals class perceive themselves to be in the bottom half of the social ladder (up to 5), a figure that rises to 40 percent among lower-grade managers and professionals. If we shift the focus to the members of lower social classes, we see a similar pattern: while 26% of the working-class members consider themselves in the three bottom rungs of the social ladder, around 33% of working class members locate themselves in its upper half. While objective social class and subjective social class match more often than not, there are sizeable mismatches, which sets the stage for our Hypotheses 3 and 4. This mismatch has significant implications, as it underscores the need to account for subjective perceptions of social position within class-based analyses, an issue that has been highlighted in recent studies (Melli, 2025).

**Figure 4:**
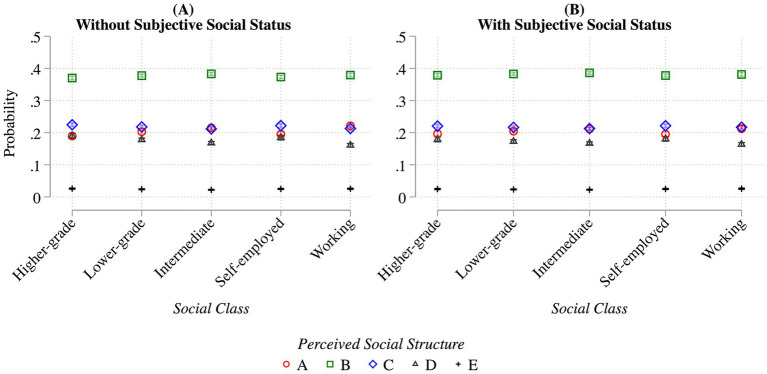
Predicted probabilities of perceived inequality structures by social class, without **(A)** and with **(B)** subjective social status. Points show predicted probabilities of choosing each perceived inequality type (A–E, see Figure 1) by objective social class. Panel A displays results from models including only social class; Panel B adds subjective social status. Estimates are based on generalized ordered logit models controlling for household income, education, social class of origin, age, gender, survey year, and country-wave fixed effects. Predictions are computed from Models 1 and 2 in Supplementary Table A5. Weighted ISSP data (1992, 1999, 2009, and 2019); *N* = 100,004.

**Figure 5:**
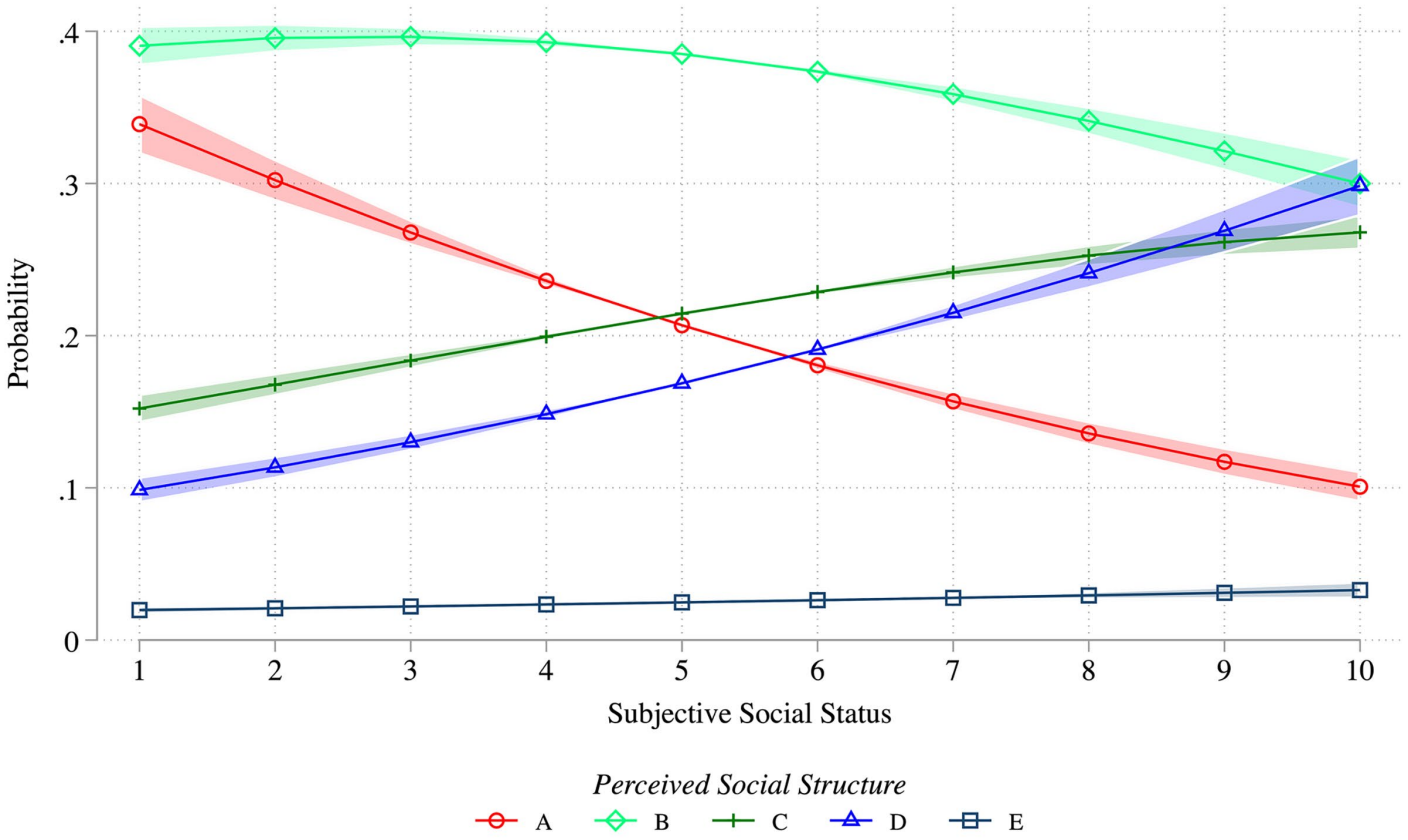
Predicted probabilities of perceived inequality structures across levels of subjective social status. Lines show predicted probabilities of selecting each perceived inequality type (A–E, see Figure 1) by subjective social status (1–10). Shaded areas represent 95% confidence intervals. Estimates are from a generalized ordered logit model controlling for household income, education, social class of origin, age, gender, survey year, and country-wave fixed effects. Predictions are based on Model 1 in Supplementary Table A5. Weighted ISSP data (1992, 1999, 2009, and 2019); *N* = 100,004.

**Figure 6:**
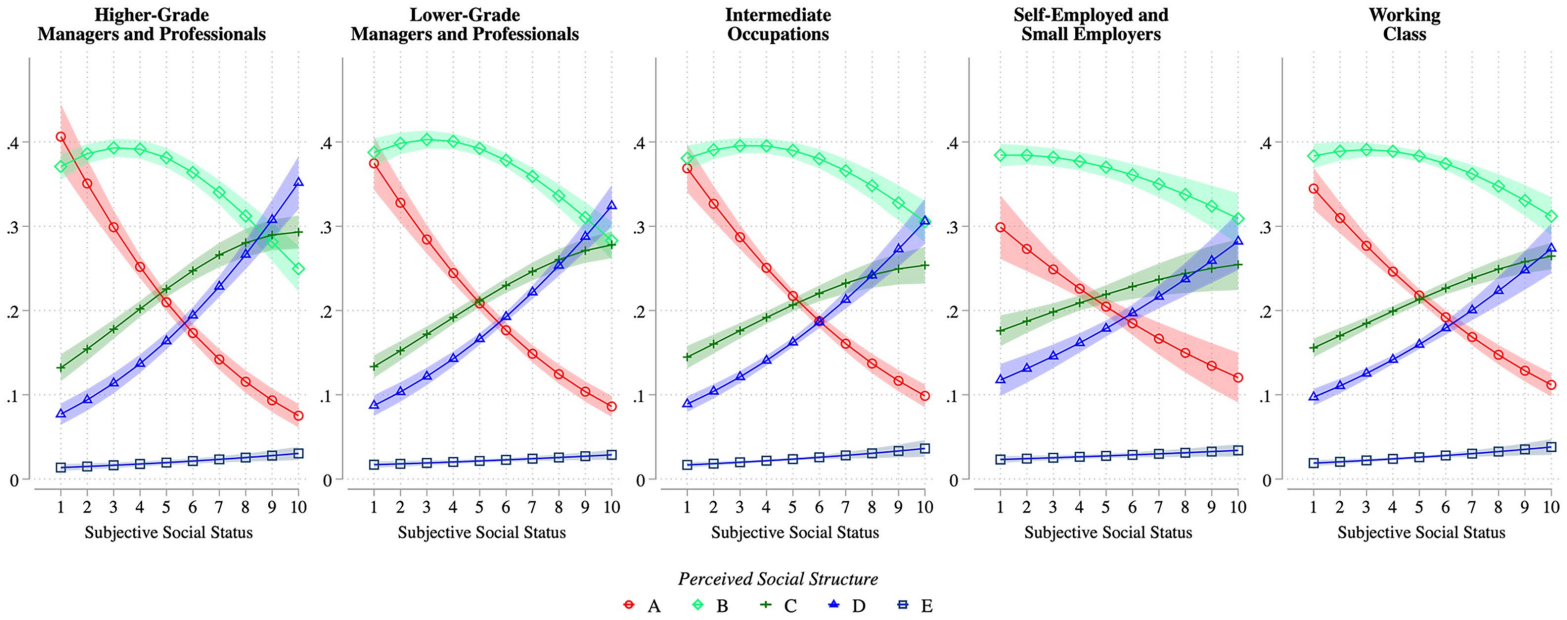
Predicted probabilities of perceived inequality structures by subjective social status within each social class. Lines show predicted probabilities of selecting each perceived inequality type (A–E, see Figure 1) across levels of subjective social status (1–10), separately for higher-grade professionals, lower-grade professionals, intermediate occupations, self-employed, and working class. Shaded areas represent 95% confidence intervals. Estimates are from a generalized ordered logit model controlling for household income, education, social class of origin, age, gender, survey year, and country-wave fixed effects. Predictions are based on Model 3 in Supplementary Table A5. Weighted ISSP data (1992, 1999, 2009, and 2019); *N* = 100,004.

Having presented descriptive results, the analysis proceeds to the more rigorous regression models. Figures 4–6 report the predicted probabilities based on the results of the Generalized Ordered Logit models. The latter are reported in full in Supplementary Table A5, owing to space limitations. Model 1 includes social class as the principal explanatory variable (see Figure 4A). Model 2 adds subjective social status (see Figures 4B, 5). Model 3 incorporates an interaction term between social class and subjective status to explore potential within-class heterogeneity, as suggested in H3-4 (see Figure 6). All models control for the full set of covariates described above and include country-wave fixed effects.

Starting with Model 1, people across different social classes tend to share broadly similar perceptions of societal structure, most frequently visualizing it as a pyramid-shaped (Type B), as illustrated in Figure 2. This general pattern is also reflected in Figure 4A, which presents the predicted probabilities of individuals from each social class selecting a particular social inequality structure. These probabilities are derived from Models 1 and 2 in Supplementary Table A5. In Panel A, subjective social status is not included in the model, although all other control variables are retained. Under this specification, individuals belonging to more advantaged classes are slightly more likely to perceive society as relatively equal (Types C and D) rather than highly unequal (Type A). This finding lends support to Hypothesis 1, which posits that individuals from lower social classes are more likely to perceive social inequalities as more pronounced than those from higher classes, albeit these differences are rather small.

However, once subjective social status is introduced into the model (Panel B), these class-based differences in perceptions largely disappear. In sum, when subjective social status is accounted for, differences in perceived societal structure between social classes, which were already relatively small to begin with, effectively vanish. The presence of within-class differences in the outcome variable driven by subjective status is also supported by a social class schema configuration with ten classes (ESEC-9 plus a category for missing, Rose and Harrison, 2007), available in Supplementary Table A7 and Supplementary Figure A1. Those results broadly reinforce the pattern that subjective social status plays a stronger role within the ESEC9 classes belonging to the Self-Employed and Working-Class groups.

Mediation analysis (Karlson et al., 2012) indicates that subjective social status accounts for 45%–90% of the association between objective social class and perceived social structure, effectively absorbing most class differences. By contrast, perceived social structure mediates less than 4 percent of the association between objective class and subjective status, suggesting that reverse causality is limited.^4^

The inclusion of subjective social status also alters the role of other covariates. The first two models in Supplementary Table A5 show that Social Class Origins follow the expected pattern: societal structure perceived as more unequal by those born in households belonging to a lower social class. The pattern is similar for household income, while having a Primary highest qualification is associated with a significantly different outcome relatively to the baseline of those with Tertiary education. Individuals with older ages are also more likely perceive society as more unequal, while gender does not play any role. Once subjective status is introduced, the coefficients for education completely lose statistical significance, and there are some reductions in both magnitude and significance for Class Origins and Household Income, although they do retain some significance. There are no changes for age and gender.

Figure 5 shows the predicted probabilities of selecting each type of social structure across levels of subjective status net of objective social class, based on estimates from Model 2 in Supplementary Table A5. Type B, the pyramid model reflecting moderate inequality, is the most frequently selected structure across nearly all levels of subjective status, with predicted probabilities consistently exceeding 40 percent, except at the highest rung. In contrast, Type E is the least frequently selected structure at all levels of subjective social status.

A clearer and symmetrical pattern emerges when comparing perceptions of the most unequal structure (Type A) with those of the more egalitarian structures (Types C and D). Individuals who place themselves at the lower end of the social ladder are more likely to view society as highly unequal and select Type A. Conversely, those who identify with the uppermost rungs of the ladder are substantially more likely to select the more equal social structure. These differences are most pronounced at the extremes of the subjective status scale. Towards the midpoint of the scale, however, the likelihood of selecting Types A, C, or D converges, generating a largely symmetrical distribution of perceptions across the range of subjective status.

Further insights on the role played by these variables arise by examining how their coefficients change when we shift from Model 1 (without subjective status) to Model 2 (Supplementary Table A5). The gaps between the highest categories (High Household Income, Tertiary Education, Higher-grade professional social origin) and the least categories are quite strong in Model 1 and arguably larger than the destination social class gap. When subjective status is introduced in Model 2, these other stratification gaps shrink but stay statistically significant, and are either comparable (for Low Household Income) or smaller in magnitude relatively to a one-point increase in the subjective status scale. This pattern remarks how subjective social status is again related to these variables but not identical. Taken together, Figures 4, 5 offer support for Hypothesis 2: individuals with lower subjective social status are more likely to perceive society as unequal than individuals with higher subjective social status, net of objective social class. Given that objective class and subjective status are related but not perfectly aligned, their interaction can generate additional insights.

The joint analysis of objective social class and subjective social status illuminates how individuals perceive inequality, as shown in Figure 6. Once again, the pyramid-shaped model (Type B), representing a society with moderate levels of inequality, is the most frequently selected option across all social classes and levels of subjective status. However, within each social class, individuals who place themselves in the lower part of the social ladder are more likely to perceive their society as highly unequal (Type A), even when their objective class position suggests relative advantage, as is the case for some members of the Higher-grade Managers and Professionals. This finding aligns with prior research indicating that subjective status plays an independent and meaningful role in shaping views about society and political attitudes more broadly (Nolan and Weisstanner, 2022; Melli and Azzollini, 2025).

Yet, the symmetrical pattern observed in Figure 5 no longer holds uniformly. A more complex pattern emerges. Specifically, the gap between the likelihood of selecting the most unequal model (Type A) and the more equal ones (Types C and D) narrows significantly at higher levels of subjective status among individuals in less advantaged social classes, disappearing altogether in the Intermediate Occupations and in the Working class. In contrast, at lower levels of subjective status, individuals are markedly more likely to select the most unequal societal model, with a pronounced gap compared to the more equal alternative.

These findings articulate the mediating role of subjective social status in the link between social class and perceived social structure, highlighting its greater explanatory power. More importantly, the strongest and most consistent differences emerge along lines of subjective social status, both across the population as a whole and within each social class. While both dimensions are strongly associated with views of the social structure, subjective status in particular uncovers patterns in the perception of social inequalities that are not readily apparent when analyzing social class alone. This underscores the importance of individuals’ self-assessed position in the social hierarchy as a key factor shaping perception of social inequality. These findings provide support for Hypotheses 3 and 4: when class and status diverge, subjective status predominates, and even within the same objective class, perceptions differ systematically by subjective social status. This pattern is particularly strong among the higher objective classes relatively to the working classes, pointing to a greater sensitivity to status-based processes at the top rather than the bottom. Overall, subjective status emerges as a stronger predictor of perceived inequality than objective class.

To test the robustness of our findings, we ran several additional analyses, reported in the Supplementary Materials. First, we conducted an additional analysis focusing on the most pronounced contrast that emerged: the likelihood of selecting Type A (the most unequal society) compared to all other categories. Supplementary Figure A5 presents the results from a logistic regression model predicting the choice of Type A as a function of social class, subjective social status, and their interaction. Full model estimates are reported in Supplementary Table A15.

As in the main analysis, we find only moderate variation across social classes in the likelihood of perceiving society as highly unequal. However, there is considerable heterogeneity within classes, particularly among those in more advantaged positions. Across all classes, individuals reporting lower subjective social status are more likely to perceive society as highly unequal than those who place themselves nearer to the top. This pattern is especially pronounced among higher-grade managers and professionals, where the gap between individuals with low and high subjective status is most evident. In contrast, the difference is smaller among the working class and appears flattest among the self-employed and small employers.

Further robustness checks include replacing subjective social status with subjective class identification (Supplementary Table A6); interacting objective class with education (Supplementary Table A17); using a more fine-grained version of the ESeC social class schema (Supplementary Table A7); and using an alternative measure of perceived inequality based on agreement with the statement ‘Income differences in [country] are too large.’ (Supplementary Table A9), whose results are broadly in line with the main analysis results: subjective social status shapes powerful differences also within objective classes, and especially within higher social classes. We also replicated results using models without survey weights and with listwise deletion. We further conducted robustness checks, extending our models to include attitudes towards redistribution through a mediation analysis, showing how perceived social structure is an important predictor of the latter. Additional models address concerns around reverse causality. Across specifications, the results remain consistent with the main analysis.

## 5 Discussion

This study examines how objective social class and subjective social status affect perceptions of social inequalities, both additively and multiplicatively. Drawing on data from 1992 to 2019 for 35 countries and 96 country-years, the analysis provides key insights into the relationship between these two dimensions of stratification and individuals’ perceptions of inequality.

Regarding Hypothesis 1, objective social class is found to influence the perception of the social inequality structure. The propensity of less advantaged social classes to perceive more unequal societies is substantial and consistent with existing literature, although relatively limited in effect size.

The relevance of objective social class for this outcome of interest changes substantially when the role of subjective social position is considered. First, these two Weberian-inspired stratification variables are clearly related but are not identical: there are substantial mismatches between them, which corroborates the importance of examining their separate and joint roles. When subjective social status is introduced (Hypothesis 2), the differences across objective classes become negligible. By contrast, subjective social status is strongly associated with the perceived social inequality structure: while the ‘standard pyramid’ remains the most frequently perceived shape of inequality, individuals who consider themselves on the lower rungs of society are more likely to perceive society as highly unequal than those on the upper rungs. As shown in Figure 5, this pattern is symmetric across the lower and upper ends of society and reaches a midpoint among those who place themselves in the middle. Introducing subjective social status into the model also reduces the magnitude of other stratification variables, such as education, origin social class, and household income, although some of these stratification gaps stay significant.

Objective social class continues to play a role when interacted with subjective social status. Given the considerable mismatches between objective and subjective positions in the social hierarchy (see Figure 3), these discrepancies also influence perceptions of the social inequality structure: when subjective social status and objective social class diverge, the former exerts a stronger effect. Similarly, among individuals belonging to the same social class but differing in the perceived social position, perceptions of inequality vary considerably. This is most evident among members of the most advantaged objective class (higher-grade managers and professionals), who display a similar probability of perceiving he most unequal social structure as those in the working class, provided that their subjective social position is low. However, when subjective status and objective class coincide, their influences reinforce one another: perceptions of society as least unequal are highest among those in the most advantaged social class with the highest subjective social status.

These findings offer three contributions to sociological research on inequality. First, this paper contributes the rapidly growing body of research on the importance of subjective social status for socio-political outcomes (Gidron and Hall, 2017; Nolan and Weisstanner, 2022; Oesch and Vigna, 2023; Melli and Azzollini, 2025; Melli, 2025), remarking how a comprehensive Weberian-inspired (1968 [1922]) framework encompassing both class and status can enhance understanding of already well-established attitudes and behavior. This framework connects research on the growing importance of subjective social status for attitudes towards redistribution (Bobzien, 2020; Kalleitner and Bobzien, 2024; Melli and Azzollini, 2025, as opposed to research focusing more on objective characteristics, see the discussion by Bobzien, 2020) with studies focusing on the precursor of perceived social inequality structure (Andersen and Curtis, 2015). More specifically, this paper integrates perspectives on class (Haddon and Wu, 2022) and status (Hajdu, 2024), which have thus far not been examined jointly in relation to perception of inequality.

If our first contribution emerges from introducing subjective social status in the literature on perceptions of inequality, the second concerns to the comparison with objective social class. While both class and status may be linked to perceptions of inequality through similar mechanisms drawn from grievance theory (Runciman, 1966; Klandermans et al., 2008) and self-interest (Bobzien, 2020), the findings remark that the *subjective* character of social status plays a key role to understand individuals’ attitudes: when the reality and perception of one’s position in society diverge, beliefs about societal inequality depend more on the latter than on the former. This is not limited to objective class. Introducing subjective status reduces, but does not completely absorb, the influence of other stratification dimensions such as education, origin class, and household income. These results underscore the importance of subjective social status for this outcome. As articulated in the literature, perceived social position is the outcome of a complex cognitive process shaped by an individual’s social network and context (Lin et al., 2001; Bobzien, 2020; Cansunar, 2021) as well as by objective stratification. Thus, subjective social status is a mediator and a complement for traditional stratification variables.

The third contribution arises from the *joint* consideration of these two stratification characteristics in interaction. By highlighting how subjective social status influences perceptions of social inequality structure differently across objective classes, and how it is associated with substantial differences in perceptions among members of the same social class, this analysis provides a more nuanced understanding of how these linked yet distinct dimensions interact in shaping perceptions of inequality.

This study presents some clear limitations. The first concerns the cross-sectional nature of the ISSP data: as individuals are not observed over time, and fluctuations in their subjective social status cannot be captured, a degree of endogeneity likely remains, which can only be mitigated through socio-demographic controls and country-wave fixed effects. This limitation also prevents a life-course approach, a perspective increasingly yielding insights into socio-political outcomes (Lersch, 2023). Such analyses may become feasible with future panel datasets including measures of perceived social inequality structure. A second limitation relates to potential reverse causality: as both subjective social status and the perceived inequality structure are attitudinal variables, the latter may influence the former, as suggested by Hajdu (2024). Although the risk cannot be entirely eliminated, it has been addressed both theoretically and empirically. Theoretically, the generation of subjective social status is understood to depend more on micro- and meso-level processes (Lin et al., 2001; Bobzien, 2020; Cansunar, 2021) than on generalized beliefs about society at large. Notably, we follow a well-established stream of literature that envisages subjective social status as the predictor of several socio-political outcomes (Gidron and Hall, 2017; Nolan and Weisstanner, 2022; Oesch and Vigna, 2023; Melli, 2025), including attitudinal variables as support for redistribution (Melli and Azzollini, 2025). Moreover, the sequence of questions in the ISSP survey supports this interpretation, as the subjective social position question consistently precedes the question on perceived social structure, aligning with established evidence on question-order effects (Schuman and Ludwig, 1983; Stark et al., 2020). Empirically, robustness checks demonstrate the limited impact of perceived social structure as a predictor of subjective social status (Supplementary Table A15), together with several other robustness checks and additional analyses.

In conclusion, this paper highlights the centrality of a joint perspective encompassing objective class and subjective status in generating new insights into well-established patterns of sociopolitical attitudes and behavior, contributing to a broader comprehension of the socio-political dynamics of contemporary democratic societies.

## Data Availability

Publicly available datasets were analyzed in this study. This data can be found at: International Social Survey Programme data available at GESIS Repository.

## Footnotes

^1^Household income is not measured uniformly across the ISSP country-waves, as the income question varies by country and survey year. To ensure comparability, income measures were recoded into tertiles within each country-year sample. This approach creates a relative income variable that allows for meaningful cross-national and longitudinal comparisons despite differences in income measurement.

^2^Reported in Supplementary Tables A3, A4.

^3^Results reported in Supplementary Tables A1, A2.

^4^Results reported in Supplementary Table A14.
