# Supplementary material for: Uncovering misperceptions of social inequalities: what matters most, objective class or subjective social status?
Source: Front Sociol. 2025 Nov 7;10:1617413. doi: 10.3389/fsoc.2025.1617413 (PMC12634657; doi:10.3389/fsoc.2025.1617413)
Supplement: Supplementary file 1 [file Data_Sheet_1.DOCX]

**Uncovering Misperceptions of Social Inequalities: What Matters Most, Objective Class or Subjective Social Status?**

***Supplementary Materials***

Table A1. Results of Multinomial Logistic Regression for Perceived Social Structure.

| **A (vs. B)** |  |
| --- | --- |
| Subjective Social Status | -0.119*** |
|  | (0.011) |
| Social Class (ref. Higher-grade professionals) |  |
| *Lower-grade professionals* | -0.052 |
|  | (0.049) |
| *Intermediate* | -0.003 |
|  | (0.049) |
| *Self-employed* | -0.029 |
|  | (0.062) |
| *Working class* | 0.015 |
|  | (0.056) |
| *NA* | -0.134** |
|  | (0.050) |
| Household Income (ref. High) |  |
| *Low* | 0.113*** |
|  | (0.034) |
| *Middle* | 0.059 |
|  | (0.036) |
| *NA* | 0.076 |
|  | (0.060) |
| Education (ref. Tertiary) |  |
| *Primary* | -0.043 |
|  | (0.050) |
| *Secondary* | -0.071+ |
|  | (0.037) |
| *NA* | -0.145* |
|  | (0.068) |
| Social Class of Origin (ref. Higher-grade professionals) |  |
| *Lower-grade professionals* | -0.046 |
|  | (0.049) |
| *Intermediate* | -0.049 |
|  | (0.049) |
| *Self-employed* | -0.129** |
|  | (0.049) |
| *Working class* | -0.077 |
|  | (0.048) |
| *NA* | -0.124* |
|  | (0.060) |
| Age | -0.001 |
|  | (0.001) |
| Female | -0.073** |
|  | (0.022) |
|  |  |
| Constant | -0.020 |
|  | (0.127) |
|  |  |
| **C (vs. B)** |  |
| Subjective Social Status | 0.076*** |
|  | (0.009) |
| Social Class (ref. Higher-grade professionals) |  |
| *Lower-grade professionals* | -0.117** |
|  | (0.040) |
| *Intermediate* | -0.141** |
|  | (0.047) |
| *Self-employed* | -0.080 |
|  | (0.063) |
| *Working class* | -0.114* |
|  | (0.045) |
| *NA* | -0.128* |
|  | (0.051) |
| Household Income (ref. High) |  |
| *Low* | -0.117** |
|  | (0.036) |
| *Middle* | -0.043 |
|  | (0.037) |
| *NA* | -0.132* |
|  | (0.052) |
| Education (ref. Tertiary) |  |
| *Primary* | -0.423*** |
|  | (0.064) |
| *Secondary* | -0.277*** |
|  | (0.041) |
| *NA* | -0.304*** |
|  | (0.081) |
| Social Class of Origin (ref. Higher-grade professionals) |  |
| *Lower-grade professionals* | -0.096+ |
|  | (0.052) |
| *Intermediate* | -0.154** |
|  | (0.059) |
| *Self-employed* | -0.136* |
|  | (0.054) |
| *Working class* | -0.263*** |
|  | (0.054) |
| *NA* | -0.182** |
|  | (0.068) |
| Age | -0.010*** |
|  | (0.001) |
| Female | -0.157*** |
|  | (0.024) |
|  |  |
| Constant | 0.679*** |
|  | (0.114) |
|  |  |
| **D (vs. B)** |  |
| Subjective Social Status | 0.153*** |
|  | (0.013) |
| Social Class (ref. Higher-grade professionals) |  |
| *Lower-grade professionals* | -0.109** |
|  | (0.040) |
| *Intermediate* | -0.151** |
|  | (0.053) |
| *Self-employed* | -0.028 |
|  | (0.062) |
| *Working class* | -0.162** |
|  | (0.053) |
| *NA* | -0.073 |
|  | (0.055) |
| Household Income (ref. High) |  |
| *Low* | -0.159*** |
|  | (0.043) |
| *Middle* | -0.104** |
|  | (0.037) |
| *NA* | -0.137** |
|  | (0.046) |
| Education (ref. Tertiary) |  |
| *Primary* | -0.298*** |
|  | (0.071) |
| *Secondary* | -0.167*** |
|  | (0.041) |
| *NA* | -0.175+ |
|  | (0.089) |
| Social Class of Origin (ref. Higher-grade professionals) |  |
| *Lower-grade professionals* | -0.119* |
|  | (0.055) |
| *Intermediate* | -0.236*** |
|  | (0.067) |
| *Self-employed* | -0.200** |
|  | (0.063) |
| *Working class* | -0.321*** |
|  | (0.062) |
| *NA* | -0.230*** |
|  | (0.068) |
| Age | -0.010*** |
|  | (0.001) |
| Female | -0.068** |
|  | (0.024) |
|  |  |
| Constant | -0.041 |
|  | (0.124) |
|  |  |
| **E (vs. B)** |  |
| Subjective Social Status | 0.049* |
|  | (0.022) |
| Social Class (ref. Higher-grade professionals) |  |
| *Lower-grade professionals* | -0.069 |
|  | (0.100) |
| *Intermediate* | -0.009 |
|  | (0.099) |
| *Self-employed* | 0.088 |
|  | (0.114) |
| *Working class* | 0.084 |
|  | (0.110) |
| *NA* | 0.074 |
|  | (0.106) |
| Household Income (ref. High) |  |
| *Low* | 0.239*** |
|  | (0.071) |
| *Middle* | -0.007 |
|  | (0.078) |
| *NA* | 0.116 |
|  | (0.078) |
| Education (ref. Tertiary) |  |
| *Primary* | 0.293** |
|  | (0.096) |
| *Secondary* | 0.004 |
|  | (0.076) |
| *NA* | 0.409** |
|  | (0.135) |
| Social Class of Origin (ref. Higher-grade professionals) |  |
| *Lower-grade professionals* | -0.036 |
|  | (0.111) |
| *Intermediate* | -0.172 |
|  | (0.116) |
| *Self-employed* | -0.151 |
|  | (0.108) |
| *Working class* | 0.026 |
|  | (0.100) |
| *NA* | -0.033 |
|  | (0.123) |
| Age | -0.010*** |
|  | (0.002) |
| Female | -0.140*** |
|  | (0.041) |
|  |  |
| Constant | -3.389*** |
|  | (0.235) |
|  |  |
|  |  |
| Country-Wave Fixed Effects | *Yes* |
| BIC | 261846 |
| AIC | 261113 |

*Note*: Data from ISSP Research Group (2024), weighted. N=100,004. The table shows relative log odds against the baseline category of the dependent variable. + p<0.10, * p<0.05, ** p<0.01, *** p<0.001

Table A2. Test for Independence of Irrelevant Alternatives for Model 1 in Table A1.

| Hausman tests of IIA assumption | | | |
| --- | --- | --- | --- |
|  | *Chi squared* | *df* | *P>Chi squared* |
| A | -164.980 | 346 |  |
| B | -450.129 | 346 |  |
| C | -100.794 | 346 |  |
| D | 174.685 | 347 | 1.000 |
| E | 42.719 | 346 | 1.000 |
| suest-based Hausman tests of IIA assumption | | | |
|  | *Chi squared* | *df* | *P>Chi squared* |
| A | 831.359 | 351 | 0.000 |
| B | 674.833 | 351 | 0.000 |
| C | 669.001 | 351 | 0.000 |
| D | 866.267 | 351 | 0.000 |
| E | 432.258 | 351 | 0.002 |

*Note*: Results of Hausman and suest-based Hausman tests of the Independence of Irrelevant Alternatives (IIA) assumption in multinomial logit models. The IIA assumption states that the odds of choosing one outcome over another are independent of the presence or characteristics of other alternatives. A significant test statistic provides evidence against IIA. N=100,004. H0: Odds(Outcome-J vs Outcome-K) are independent of other alternatives. Data from ISSP Research Group (2024).

Table A3. Results of Ordinal Logistic Regressions for Perceived Social Structure.

| Subjective Social Status | 0.152*** |
| --- | --- |
|  | (0.009) |
| Social Class (ref. Higher-grade professionals) |  |
| *Lower-grade professionals* | -0.033 |
|  | (0.023) |
| *Intermediate* | -0.070* |
|  | (0.029) |
| *Self-employed* | 0.021 |
|  | (0.034) |
| *Working class* | -0.069* |
|  | (0.032) |
| *NA* | 0.046 |
|  | (0.030) |
| Household Income (ref. High) |  |
| *Low* | -0.113*** |
|  | (0.025) |
| *Middle* | -0.083*** |
|  | (0.020) |
| *NA* | -0.100** |
|  | (0.031) |
| Education (ref. Tertiary) |  |
| *Primary* | -0.133** |
|  | (0.041) |
| *Secondary* | -0.070** |
|  | (0.025) |
| *NA* | 0.018 |
|  | (0.065) |
| Social Class of Origin (ref. Higher-grade professionals) |  |
| *Lower-grade professionals* | -0.042 |
|  | (0.031) |
| *Intermediate* | -0.102** |
|  | (0.037) |
| *Self-employed* | -0.047 |
|  | (0.032) |
| *Working class* | -0.125*** |
|  | (0.037) |
| *NA* | -0.052 |
|  | (0.046) |
| Age | -0.007*** |
|  | (0.001) |
| Female | -0.030+ |
|  | (0.016) |
|  |  |
| cut1 | -1.496*** |
|  | (0.100) |
|  |  |
| cut2 | 0.184* |
|  | (0.091) |
| cut3 | 1.293*** |
|  | (0.089) |
|  |  |
| cut4 | 3.719*** |
|  | (0.151) |
|  |  |
| Country-Wave Fixed Effects | *Yes* |
| BIC | 268868 |
| AIC | 268659 |

*Note*: N=100,004. Data from ISSP Research Group (2024). + p<0.10, * p<0.05, ** p<0.01, *** p<0.001

Table A4. Brant Test of Parallel Regression Assumption for Model 1 in Table A3.

| *Variable* | *chi2* | *p>chi2* | *df* |
| --- | --- | --- | --- |
| All | 7766.81 | 0.000 | 342 |
| Subjective Social Status | 114.29 | 0.000 | 3 |
| Social Class (ref. Higher-grade professionals) |  |  |  |
| *Lower-grade professionals* | 7.39 | 0.060 | 3 |
| *Intermediate* | 10.01 | 0.019 | 3 |
| *Self-Employed* | 9.06 | 0.028 | 3 |
| *Working class* | 11.92 | 0.008 | 3 |
| *NA* | 19.31 | 0.000 | 3 |
| Household Income (ref. High) |  |  |  |
| *Low* | 63.76 | 0.000 | 3 |
| *Middle* | 5.57 | 0.134 | 3 |
| *NA* | 26.41 | 0.000 | 3 |
| Education (ref. Tertiary) |  |  |  |
| *Primary* | 191.77 | 0.000 | 3 |
| *Secondary* | 96.34 | 0.000 | 3 |
| *NA* | 69.89 | 0.000 | 3 |
| Social Class of Origin (ref. Higher-grade professionals) |  |  |  |
| *Lower-grade professionals* | 3.68 | 0.298 | 3 |
| *Intermediate* | 7.75 | 0.051 | 3 |
| *Self-Employed* | 8.62 | 0.035 | 3 |
| *Working class* | 40.71 | 0.000 | 3 |
| *NA* | 20.18 | 0.000 | 3 |
| Age | 103.17 | 0.000 | 3 |
| Female | 54.15 | 0.000 | 3 |

*Note*: Results of Brant tests assessing whether the proportional odds (parallel regression) assumption holds for ordered logit models. A significant chi-squared statistic indicates violation of the assumption for the variable tested. The “All” row reports the global test across predictors; individual rows show tests for each variable. Data from ISSP Research Group (2024). N=100,004.

Table A5. Results of Generalised Ordered Logit Models for Ordinal Dependent Variables for Perceived Social Structure.

|  | (1) | (2) | (3) |
| --- | --- | --- | --- |
|  | Social Class | SSS and Social Class | Interaction Social Class * SSS |
|  |  |  |  |
| **A** |  |  |  |
| Social Class (ref. Higher-grade professionals) |  |  |  |
| *Lower-grade professionals* | -0.084** | -0.048+ | 0.164 |
|  | (0.026) | (0.026) | (0.107) |
| *Intermediate* | -0.158*** | -0.092** | 0.209+ |
|  | (0.033) | (0.033) | (0.119) |
| *Self-employed* | -0.035 | 0.010 | 0.584*** |
|  | (0.039) | (0.037) | (0.158) |
| *Working class* | -0.195*** | -0.096* | 0.341** |
|  | (0.040) | (0.040) | (0.115) |
| *NA* | 0.001 | 0.064+ | 0.521*** |
|  | (0.038) | (0.038) | (0.105) |
| Subjective Social Status |  | 0.169*** | 0.237*** |
|  |  | (0.012) | (0.019) |
| Social Class * SSS |  |  |  |
| *Lower-grade professionals * SSS* |  |  | -0.031+ |
|  |  |  | (0.019) |
| *Intermediate * SSS* |  |  | -0.051* |
|  |  |  | (0.022) |
| *Self-employed * SSS* |  |  | -0.110*** |
|  |  |  | (0.033) |
| *Working class * SSS* |  |  | -0.078*** |
|  |  |  | (0.023) |
| *NA * SSS* |  |  | -0.082*** |
|  |  |  | (0.022) |
| Household Income (ref. High) |  |  |  |
| *Low* | -0.337*** | -0.170*** | -0.167*** |
|  | (0.038) | (0.032) | (0.031) |
| *Middle* | -0.178*** | -0.096*** | -0.092*** |
|  | (0.024) | (0.021) | (0.021) |
| *NA* | -0.196*** | -0.150** | -0.145** |
|  | (0.052) | (0.053) | (0.053) |
| Education (ref. Tertiary) |  |  |  |
| *Primary* | -0.252*** | -0.137** | -0.130** |
|  | (0.051) | (0.049) | (0.048) |
| *Secondary* | -0.114** | -0.058 | -0.045 |
|  | (0.037) | (0.036) | (0.034) |
| *NA* | -0.145* | 0.022 | 0.021 |
|  | (0.071) | (0.075) | (0.073) |
| Social Class of Origin (ref. Higher-grade professionals) |  |  |  |
| *Lower-grade professionals* | -0.073* | -0.052 | -0.049 |
|  | (0.034) | (0.034) | (0.034) |
| *Intermediate* | -0.153*** | -0.124** | -0.121** |
|  | (0.042) | (0.041) | (0.041) |
| *Self-employed* | -0.105** | -0.065+ | -0.062+ |
|  | (0.037) | (0.036) | (0.036) |
| *Working class* | -0.188*** | -0.136** | -0.132** |
|  | (0.041) | (0.041) | (0.041) |
| *NA* | -0.114* | -0.071 | -0.068 |
|  | (0.050) | (0.050) | (0.050) |
| Age | -0.005*** | -0.004*** | -0.004*** |
|  | (0.001) | (0.001) | (0.001) |
| Female | -0.001 | 0.013 | 0.014 |
|  | (0.022) | (0.022) | (0.022) |
|  |  |  |  |
| Constant | 2.673*** | 1.421*** | 1.010*** |
|  | (0.090) | (0.115) | (0.111) |
|  |  |  |  |
| **B** |  |  |  |
| Social Class (ref. Higher-grade professionals) |  |  |  |
| *Lower-grade professionals* | -0.084** | -0.048+ | 0.116 |
|  | (0.026) | (0.026) | (0.120) |
| *Intermediate* | -0.158*** | -0.092** | 0.209+ |
|  | (0.033) | (0.033) | (0.119) |
| *Self-employed* | -0.035 | 0.010 | 0.584*** |
|  | (0.039) | (0.037) | (0.158) |
| *Working class* | -0.168*** | -0.075* | 0.341** |
|  | (0.038) | (0.037) | (0.115) |
| *NA* | -0.057 | -0.002 | 0.449*** |
|  | (0.039) | (0.038) | (0.124) |
| Subjective Social Status |  | 0.155*** | 0.220*** |
|  |  | (0.010) | (0.018) |
| Social Class * SSS |  |  |  |
| *Lower-grade professionals * SSS* |  |  | -0.031+ |
|  |  |  | (0.019) |
| *Intermediate * SSS* |  |  | -0.055** |
|  |  |  | (0.020) |
| *Self-employed * SSS* |  |  | -0.103*** |
|  |  |  | (0.030) |
| *Working class * SSS* |  |  | -0.077*** |
|  |  |  | (0.021) |
| *NA * SSS* |  |  | -0.082*** |
|  |  |  | (0.022) |
| Household Income (ref. High) |  |  |  |
| *Low* | -0.302*** | -0.148*** | -0.146*** |
|  | (0.036) | (0.031) | (0.031) |
| *Middle* | -0.178*** | -0.096*** | -0.092*** |
|  | (0.024) | (0.021) | (0.021) |
| *NA* | -0.186*** | -0.132*** | -0.128*** |
|  | (0.035) | (0.035) | (0.035) |
| Education (ref. Tertiary) |  |  |  |
| *Primary* | -0.392*** | -0.294*** | -0.285*** |
|  | (0.060) | (0.056) | (0.058) |
| *Secondary* | -0.248*** | -0.200*** | -0.185*** |
|  | (0.036) | (0.034) | (0.036) |
| *NA* | -0.256*** | -0.120 | -0.120 |
|  | (0.076) | (0.080) | (0.080) |
| Social Class of Origin (ref. Higher-grade professionals) |  |  |  |
| *Lower-grade professionals* | -0.073* | -0.052 | -0.049 |
|  | (0.034) | (0.034) | (0.034) |
| *Intermediate* | -0.153*** | -0.124** | -0.121** |
|  | (0.042) | (0.041) | (0.041) |
| *Self-employed* | -0.105** | -0.065+ | -0.062+ |
|  | (0.037) | (0.036) | (0.036) |
| *Working class* | -0.235*** | -0.187*** | -0.182*** |
|  | (0.043) | (0.044) | (0.043) |
| *NA* | -0.114* | -0.071 | -0.068 |
|  | (0.050) | (0.050) | (0.050) |
| Age | -0.010*** | -0.010*** | -0.010*** |
|  | (0.001) | (0.001) | (0.001) |
| Female | -0.094*** | -0.084*** | -0.080*** |
|  | (0.020) | (0.019) | (0.019) |
|  |  |  |  |
| Constant | 1.389*** | 0.243* | -0.139 |
|  | (0.102) | (0.102) | (0.128) |
|  |  |  |  |
| **C** |  |  |  |
| Social Class (ref. Higher-grade professionals) |  |  |  |
| *Lower-grade professionals* | -0.084** | -0.048+ | 0.186 |
|  | (0.026) | (0.026) | (0.126) |
| *Intermediate* | -0.158*** | -0.092** | 0.209+ |
|  | (0.033) | (0.033) | (0.119) |
| *Self-employed* | -0.035 | 0.010 | 0.584*** |
|  | (0.039) | (0.037) | (0.158) |
| *Working class* | -0.182*** | -0.092* | 0.341** |
|  | (0.042) | (0.042) | (0.115) |
| *NA* | -0.027 | 0.026 | 0.553*** |
|  | (0.039) | (0.038) | (0.133) |
| Subjective Social Status |  | 0.145*** | 0.203*** |
|  |  | (0.010) | (0.018) |
| Social Class * SSS |  |  |  |
| *Lower-grade professionals * SSS* |  |  | -0.031+ |
|  |  |  | (0.019) |
| *Intermediate * SSS* |  |  | -0.038* |
|  |  |  | (0.019) |
| *Self-employed * SSS* |  |  | -0.088*** |
|  |  |  | (0.025) |
| *Working class * SSS* |  |  | -0.065*** |
|  |  |  | (0.019) |
| *NA * SSS* |  |  | -0.082*** |
|  |  |  | (0.022) |
| Household Income (ref. High) |  |  |  |
| *Low* | -0.227*** | -0.082** | -0.082** |
|  | (0.033) | (0.030) | (0.030) |
| *Middle* | -0.178*** | -0.096*** | -0.092*** |
|  | (0.024) | (0.021) | (0.021) |
| *NA* | -0.114*** | -0.060+ | -0.057+ |
|  | (0.033) | (0.034) | (0.034) |
| Education (ref. Tertiary) |  |  |  |
| *Primary* | -0.123* | -0.031 | -0.040 |
|  | (0.053) | (0.051) | (0.049) |
| *Secondary* | -0.053 | -0.008 | -0.011 |
|  | (0.033) | (0.032) | (0.031) |
| *NA* | -0.039 | 0.080 | 0.064 |
|  | (0.067) | (0.069) | (0.069) |
| Social Class of Origin (ref. Higher-grade professionals) |  |  |  |
| *Lower-grade professionals* | -0.073* | -0.052 | -0.049 |
|  | (0.034) | (0.034) | (0.034) |
| *Intermediate* | -0.153*** | -0.124** | -0.121** |
|  | (0.042) | (0.041) | (0.041) |
| *Self-employed* | -0.105** | -0.065+ | -0.062+ |
|  | (0.037) | (0.036) | (0.036) |
| *Working class* | -0.169*** | -0.122** | -0.119** |
|  | (0.044) | (0.045) | (0.045) |
| *NA* | -0.114* | -0.071 | -0.068 |
|  | (0.050) | (0.050) | (0.050) |
| Age | -0.007*** | -0.007*** | -0.007*** |
|  | (0.001) | (0.001) | (0.001) |
| Female | -0.021 | -0.011 | -0.013 |
|  | (0.018) | (0.018) | (0.018) |
|  |  |  |  |
| Constant | -0.330*** | -1.417*** | -1.813*** |
|  | (0.087) | (0.102) | (0.115) |
|  |  |  |  |
| **D** |  |  |  |
| Social Class (ref. Higher-grade professionals) |  |  |  |
| *Lower-grade professionals* | -0.084** | -0.048+ | 0.253 |
|  | (0.026) | (0.026) | (0.164) |
| *Intermediate* | -0.158*** | -0.092** | 0.209+ |
|  | (0.033) | (0.033) | (0.119) |
| *Self-employed* | -0.035 | 0.010 | 0.584*** |
|  | (0.039) | (0.037) | (0.158) |
| *Working class* | -0.014 | 0.043 | 0.341** |
|  | (0.059) | (0.059) | (0.115) |
| *NA* | 0.063 | 0.114* | 0.769*** |
|  | (0.055) | (0.055) | (0.161) |
| Subjective Social Status |  | 0.058** | 0.091*** |
|  |  | (0.019) | (0.028) |
| Social Class * SSS |  |  |  |
| *Lower-grade professionals * SSS* |  |  | -0.031+ |
|  |  |  | (0.019) |
| *Intermediate * SSS* |  |  | -0.003 |
|  |  |  | (0.024) |
| *Self-employed * SSS* |  |  | -0.047+ |
|  |  |  | (0.027) |
| *Working class * SSS* |  |  | -0.011 |
|  |  |  | (0.023) |
| *NA * SSS* |  |  | -0.082*** |
|  |  |  | (0.022) |
| Household Income (ref. High) |  |  |  |
| *Low* | 0.081+ | 0.163*** | 0.160*** |
|  | (0.047) | (0.049) | (0.049) |
| *Middle* | -0.178*** | -0.096*** | -0.092*** |
|  | (0.024) | (0.021) | (0.021) |
| *NA* | 0.080 | 0.119 | 0.123+ |
|  | (0.076) | (0.074) | (0.075) |
| Education (ref. Tertiary) |  |  |  |
| *Primary* | 0.428*** | 0.441*** | 0.371*** |
|  | (0.091) | (0.092) | (0.093) |
| *Secondary* | 0.157* | 0.157* | 0.101 |
|  | (0.076) | (0.075) | (0.076) |
| *NA* | 0.517*** | 0.543*** | 0.468*** |
|  | (0.140) | (0.134) | (0.137) |
| Social Class of Origin (ref. Higher-grade professionals) |  |  |  |
| *Lower-grade professionals* | -0.073* | -0.052 | -0.049 |
|  | (0.034) | (0.034) | (0.034) |
| *Intermediate* | -0.153*** | -0.124** | -0.121** |
|  | (0.042) | (0.041) | (0.041) |
| *Self-employed* | -0.105** | -0.065+ | -0.062+ |
|  | (0.037) | (0.036) | (0.036) |
| *Working class* | 0.050 | 0.089+ | 0.094+ |
|  | (0.054) | (0.053) | (0.054) |
| *NA* | -0.114* | -0.071 | -0.068 |
|  | (0.050) | (0.050) | (0.050) |
| Age | -0.006*** | -0.007*** | -0.006*** |
|  | (0.002) | (0.002) | (0.002) |
| Female | -0.069* | -0.069* | -0.071* |
|  | (0.034) | (0.035) | (0.036) |
|  |  |  |  |
| Constant | -4.387*** | -4.845*** | -5.185*** |
|  | (0.105) | (0.175) | (0.226) |
|  |  |  |  |
|  |  |  |  |
| Country-Wave Fixed Effects | *Yes* | *Yes* | *Yes* |
| BIC | 259456 | 257732 | 256935 |
| AIC | 258952 | 257228 | 256297 |

*Note*: Data from ISSP Research Group (2024), weighted. N=100,004. + p<0.10, * p<0.05, ** p<0.01, *** p<0.001

Table A6. Results of Generalised Ordered Logit Models for Ordinal Dependent Variables for Perceived Social Structure, replication with Class Identification.

|  | (1) | (2) |
| --- | --- | --- |
|  | M1 | M2 |
|  |  |  |
| **A** |  |  |
| Class Identification (ref. Upper class) |  |  |
| *Middle class* | -0.316*** | -0.363*** |
|  | (0.029) | (0.047) |
| *Working class* | -0.679*** | -0.900*** |
|  | (0.049) | (0.095) |
| *NA* | -0.503*** | -0.420*** |
|  | (0.063) | (0.114) |
| Social Class (ref. Higher-grade professionals) |  |  |
| *Lower-grade professionals* | -0.058* | -0.069 |
|  | (0.025) | (0.051) |
| *Intermediate* | -0.104** | -0.146* |
|  | (0.033) | (0.069) |
| *Self-employed* | 0.004 | -0.201* |
|  | (0.038) | (0.096) |
| *Working class* | -0.092* | -0.166+ |
|  | (0.040) | (0.085) |
| *NA* | 0.041 | -0.002 |
|  | (0.037) | (0.083) |
| Household Income (ref. High) |  |  |
| *Low* | -0.245*** | -0.243*** |
|  | (0.031) | (0.030) |
| *Middle* | -0.118*** | -0.116*** |
|  | (0.022) | (0.022) |
| *NA* | -0.165*** | -0.165** |
|  | (0.050) | (0.050) |
| Education (ref. Tertiary) |  |  |
| *Primary* | -0.150** | -0.151** |
|  | (0.047) | (0.047) |
| *Secondary* | -0.065+ | -0.064+ |
|  | (0.036) | (0.036) |
| *NA* | -0.040 | -0.046 |
|  | (0.068) | (0.067) |
| Social Class of Origin (ref. Higher-grade professionals) |  |  |
| *Lower-grade professionals* | -0.049 | -0.048 |
|  | (0.034) | (0.034) |
| *Intermediate* | -0.111** | -0.109** |
|  | (0.042) | (0.042) |
| *Self-employed* | -0.050 | -0.046 |
|  | (0.037) | (0.036) |
| *Working class* | -0.117** | -0.114** |
|  | (0.040) | (0.040) |
| *NA* | -0.052 | -0.049 |
|  | (0.050) | (0.050) |
| Age | -0.005*** | -0.005*** |
|  | (0.001) | (0.001) |
| Female | -0.003 | -0.001 |
|  | (0.023) | (0.023) |
| Social Class * Class Identification |  |  |
| *Lower grade professionals * Middle class* |  | 0.013 |
|  |  | (0.052) |
| *Lower grade professionals * Working class* |  | 0.153+ |
|  |  | (0.091) |
| *Lower grade professionals * NA* |  | -0.120 |
|  |  | (0.104) |
| *Intermediate * Middle class* |  | 0.071 |
|  |  | (0.073) |
| *Intermediate * Working class* |  | 0.200+ |
|  |  | (0.114) |
| *Intermediate * NA* |  | -0.239 |
|  |  | (0.176) |
| *Self-employed * Middle class* |  | 0.159 |
|  |  | (0.102) |
| *Self-employed * Working class* |  | 0.482*** |
|  |  | (0.144) |
| *Self-employed * NA* |  | 0.123 |
|  |  | (0.164) |
| *Working class * Middle class* |  | 0.083 |
|  |  | (0.091) |
| *Working class * Working class* |  | 0.263* |
|  |  | (0.109) |
| *Working class * NA* |  | -0.207 |
|  |  | (0.145) |
| *NA * Middle class* |  | 0.043 |
|  |  | (0.083) |
| *NA * Working class* |  | 0.196+ |
|  |  | (0.117) |
| *NA * NA* |  | 0.016 |
|  |  | (0.134) |
|  |  |  |
| Constant | 2.920*** | 2.964*** |
|  | (0.098) | (0.106) |
|  |  |  |
| **B** |  |  |
| Class Identification (ref. Upper class) |  |  |
| *Middle class* | -0.316*** | -0.363*** |
|  | (0.029) | (0.047) |
| *Working class* | -0.670*** | -0.891*** |
|  | (0.042) | (0.098) |
| *NA* | -0.503*** | -0.420*** |
|  | (0.063) | (0.114) |
| Social Class (ref. Higher-grade professionals) |  |  |
| *Lower-grade professionals* | -0.058* | -0.069 |
|  | (0.025) | (0.051) |
| *Intermediate* | -0.104** | -0.146* |
|  | (0.033) | (0.069) |
| *Self-employed* | 0.004 | -0.201* |
|  | (0.038) | (0.096) |
| *Working class* | -0.067+ | -0.140 |
|  | (0.036) | (0.085) |
| *NA* | -0.020 | -0.065 |
|  | (0.038) | (0.079) |
| Household Income (ref. High) |  |  |
| *Low* | -0.208*** | -0.206*** |
|  | (0.031) | (0.030) |
| *Middle* | -0.118*** | -0.116*** |
|  | (0.022) | (0.022) |
| *NA* | -0.149*** | -0.149*** |
|  | (0.035) | (0.035) |
| Education (ref. Tertiary) |  |  |
| *Primary* | -0.299*** | -0.297*** |
|  | (0.058) | (0.057) |
| *Secondary* | -0.204*** | -0.200*** |
|  | (0.035) | (0.035) |
| *NA* | -0.155* | -0.158* |
|  | (0.075) | (0.073) |
| Social Class of Origin (ref. Higher-grade professionals) |  |  |
| *Lower-grade professionals* | -0.049 | -0.048 |
|  | (0.034) | (0.034) |
| *Intermediate* | -0.111** | -0.109** |
|  | (0.042) | (0.042) |
| *Self-employed* | -0.050 | -0.046 |
|  | (0.037) | (0.036) |
| *Working class* | -0.165*** | -0.162*** |
|  | (0.043) | (0.042) |
| *NA* | -0.052 | -0.049 |
|  | (0.050) | (0.050) |
| Age | -0.010*** | -0.010*** |
|  | (0.001) | (0.001) |
| Female | -0.096*** | -0.095*** |
|  | (0.020) | (0.020) |
| Social Class * Class Identification |  |  |
| *Lower grade professionals * Middle class* |  | 0.013 |
|  |  | (0.052) |
| *Lower grade professionals * Working class* |  | 0.153+ |
|  |  | (0.091) |
| *Lower grade professionals * NA* |  | -0.120 |
|  |  | (0.104) |
| *Intermediate * Middle class* |  | 0.071 |
|  |  | (0.073) |
| *Intermediate * Working class* |  | 0.200+ |
|  |  | (0.114) |
| *Intermediate * NA* |  | -0.239 |
|  |  | (0.176) |
| *Self-employed * Middle class* |  | 0.159 |
|  |  | (0.102) |
| *Self-employed * Working class* |  | 0.482*** |
|  |  | (0.144) |
| *Self-employed * NA* |  | 0.123 |
|  |  | (0.164) |
| *Working class * Middle class* |  | 0.083 |
|  |  | (0.091) |
| *Working class * Working class* |  | 0.263* |
|  |  | (0.109) |
| *Working class * NA* |  | -0.207 |
|  |  | (0.145) |
| *NA * Middle class* |  | 0.043 |
|  |  | (0.083) |
| *NA * Working class* |  | 0.196+ |
|  |  | (0.117) |
| *NA * NA* |  | 0.016 |
|  |  | (0.134) |
|  |  |  |
| Constant | 1.638*** | 1.680*** |
|  | (0.106) | (0.111) |
|  |  |  |
| **C** |  |  |
| Class Identification (ref. Upper class) |  |  |
| *Middle class* | -0.316*** | -0.363*** |
|  | (0.029) | (0.047) |
| *Working class* | -0.661*** | -0.882*** |
|  | (0.041) | (0.094) |
| *NA* | -0.503*** | -0.420*** |
|  | (0.063) | (0.114) |
| Social Class (ref. Higher-grade professionals) |  |  |
| *Lower-grade professionals* | -0.058* | -0.069 |
|  | (0.025) | (0.051) |
| *Intermediate* | -0.104** | -0.146* |
|  | (0.033) | (0.069) |
| *Self-employed* | 0.004 | -0.201* |
|  | (0.038) | (0.096) |
| *Working class* | -0.082+ | -0.155+ |
|  | (0.042) | (0.084) |
| *NA* | 0.008 | -0.037 |
|  | (0.037) | (0.078) |
| Household Income (ref. High) |  |  |
| *Low* | -0.133*** | -0.131*** |
|  | (0.028) | (0.028) |
| *Middle* | -0.118*** | -0.116*** |
|  | (0.022) | (0.022) |
| *NA* | -0.077* | -0.076* |
|  | (0.034) | (0.034) |
| Education (ref. Tertiary) |  |  |
| *Primary* | -0.032 | -0.028 |
|  | (0.050) | (0.050) |
| *Secondary* | -0.009 | -0.005 |
|  | (0.033) | (0.033) |
| *NA* | 0.058 | 0.056 |
|  | (0.065) | (0.064) |
| Social Class of Origin (ref. Higher-grade professionals) |  |  |
| *Lower-grade professionals* | -0.049 | -0.048 |
|  | (0.034) | (0.034) |
| *Intermediate* | -0.111** | -0.109** |
|  | (0.042) | (0.042) |
| *Self-employed* | -0.050 | -0.046 |
|  | (0.037) | (0.036) |
| *Working class* | -0.099* | -0.096* |
|  | (0.043) | (0.043) |
| *NA* | -0.052 | -0.049 |
|  | (0.050) | (0.050) |
| Age | -0.007*** | -0.007*** |
|  | (0.001) | (0.001) |
| Female | -0.022 | -0.021 |
|  | (0.018) | (0.018) |
| Social Class * Class Identification |  |  |
| *Lower grade professionals * Middle class* |  | 0.013 |
|  |  | (0.052) |
| *Lower grade professionals * Working class* |  | 0.153+ |
|  |  | (0.091) |
| *Lower grade professionals * NA* |  | -0.120 |
|  |  | (0.104) |
| *Intermediate * Middle class* |  | 0.071 |
|  |  | (0.073) |
| *Intermediate * Working class* |  | 0.200+ |
|  |  | (0.114) |
| *Intermediate * NA* |  | -0.239 |
|  |  | (0.176) |
| *Self-employed * Middle class* |  | 0.159 |
|  |  | (0.102) |
| *Self-employed * Working class* |  | 0.482*** |
|  |  | (0.144) |
| *Self-employed * NA* |  | 0.123 |
|  |  | (0.164) |
| *Working class * Middle class* |  | 0.083 |
|  |  | (0.091) |
| *Working class * Working class* |  | 0.263* |
|  |  | (0.109) |
| *Working class * NA* |  | -0.207 |
|  |  | (0.145) |
| *NA * Middle class* |  | 0.043 |
|  |  | (0.083) |
| *NA * Working class* |  | 0.196+ |
|  |  | (0.117) |
| *NA * NA* |  | 0.016 |
|  |  | (0.134) |
|  |  |  |
| Constant | -0.088 | -0.048 |
|  | (0.092) | (0.098) |
|  |  |  |
| **D** |  |  |
| Class Identification (ref. Upper class) |  |  |
| *Middle class* | -0.316*** | -0.363*** |
|  | (0.029) | (0.047) |
| *Working class* | -0.361*** | -0.583*** |
|  | (0.070) | (0.115) |
| *NA* | -0.503*** | -0.420*** |
|  | (0.063) | (0.114) |
| Social Class (ref. Higher-grade professionals) |  |  |
| *Lower-grade professionals* | -0.058* | -0.069 |
|  | (0.025) | (0.051) |
| *Intermediate* | -0.104** | -0.146* |
|  | (0.033) | (0.069) |
| *Self-employed* | 0.004 | -0.201* |
|  | (0.038) | (0.096) |
| *Working class* | 0.040 | -0.031 |
|  | (0.056) | (0.089) |
| *NA* | 0.103+ | 0.060 |
|  | (0.053) | (0.086) |
| Household Income (ref. High) |  |  |
| *Low* | 0.136** | 0.138** |
|  | (0.049) | (0.049) |
| *Middle* | -0.118*** | -0.116*** |
|  | (0.022) | (0.022) |
| *NA* | 0.117 | 0.118 |
|  | (0.076) | (0.076) |
| Education (ref. Tertiary) |  |  |
| *Primary* | 0.459*** | 0.463*** |
|  | (0.086) | (0.086) |
| *Secondary* | 0.176* | 0.181* |
|  | (0.075) | (0.075) |
| *NA* | 0.563*** | 0.562*** |
|  | (0.134) | (0.135) |
| Social Class of Origin (ref. Higher-grade professionals) |  |  |
| *Lower-grade professionals* | -0.049 | -0.048 |
|  | (0.034) | (0.034) |
| *Intermediate* | -0.111** | -0.109** |
|  | (0.042) | (0.042) |
| *Self-employed* | -0.050 | -0.046 |
|  | (0.037) | (0.036) |
| *Working class* | 0.103+ | 0.106* |
|  | (0.053) | (0.053) |
| *NA* | -0.052 | -0.049 |
|  | (0.050) | (0.050) |
| Age | -0.007*** | -0.007*** |
|  | (0.002) | (0.002) |
| Female | -0.071* | -0.071* |
|  | (0.035) | (0.035) |
| Social Class * Class Identification |  |  |
| *Lower grade professionals * Middle class* |  | 0.013 |
|  |  | (0.052) |
| *Lower grade professionals * Working class* |  | 0.153+ |
|  |  | (0.091) |
| *Lower grade professionals * NA* |  | -0.120 |
|  |  | (0.104) |
| *Intermediate * Middle class* |  | 0.071 |
|  |  | (0.073) |
| *Intermediate * Working class* |  | 0.200+ |
|  |  | (0.114) |
| *Intermediate * NA* |  | -0.239 |
|  |  | (0.176) |
| *Self-employed * Middle class* |  | 0.159 |
|  |  | (0.102) |
| *Self-employed * Working class* |  | 0.482*** |
|  |  | (0.144) |
| *Self-employed * NA* |  | 0.123 |
|  |  | (0.164) |
| *Working class * Middle class* |  | 0.083 |
|  |  | (0.091) |
| *Working class * Working class* |  | 0.263* |
|  |  | (0.109) |
| *Working class * NA* |  | -0.207 |
|  |  | (0.145) |
| *NA * Middle class* |  | 0.043 |
|  |  | (0.083) |
| *NA * Working class* |  | 0.196+ |
|  |  | (0.117) |
| *NA * NA* |  | 0.016 |
|  |  | (0.134) |
| Constant | -4.184*** | -4.144*** |
|  | (0.107) | (0.111) |
|  |  |  |
|  |  |  |
| Country-Wave Fixed Effects | *Yes* | *Yes* |
| BIC | 258615 | 258730 |
| AIC | 258092 | 258064 |

*Note*: Data from ISSP Research Group (2024), weighted. N=100,004. + p<0.10, * p<0.05, ** p<0.01, *** p<0.001

Table A7. Results of Generalised Ordered Logit Models for Ordinal Dependent Variables for Perceived Social Structure, replication with full ESeC schema.

|  | (1) | (2) | (3) |
| --- | --- | --- | --- |
|  | M1 | M2 | M3 |
|  |  |  |  |
| **A** |  |  |  |
| Social Class (ref. Large employers, higher mgrs/professionals) |  |  |  |
| *Lower mgrs/professionals, higher supervisory/technicians* | -0.084** | -0.048+ | 0.143 |
|  | (0.026) | (0.026) | (0.108) |
| *Intermediate occupations* | -0.156*** | -0.102** | 0.170 |
|  | (0.035) | (0.036) | (0.118) |
| *Small employers and self-employed (non-agriculture)* | -0.056 | -0.017 | 0.554*** |
|  | (0.042) | (0.040) | (0.148) |
| *Small employers and self-employed (agriculture)* | 0.053 | 0.138** | 0.517** |
|  | (0.055) | (0.053) | (0.200) |
| *Lower supervisors and technicians* | -0.129** | -0.065 | 0.275+ |
|  | (0.047) | (0.047) | (0.143) |
| *Lower sales and service* | -0.162*** | -0.081* | 0.188 |
|  | (0.039) | (0.039) | (0.129) |
| *Lower technical* | -0.183*** | -0.077+ | 0.293* |
|  | (0.044) | (0.044) | (0.120) |
| *Routine* | -0.213*** | -0.091* | 0.353** |
|  | (0.043) | (0.039) | (0.124) |
| *NA* | -0.026 | 0.070+ | 0.492*** |
|  | (0.034) | (0.038) | (0.110) |
| Household Income (ref. High) |  |  |  |
| *Low* | -0.334*** | -0.172*** | -0.170*** |
|  | (0.038) | (0.032) | (0.032) |
| *Middle* | -0.178*** | -0.097*** | -0.092*** |
|  | (0.024) | (0.021) | (0.021) |
| *NA* | -0.193*** | -0.150** | -0.146** |
|  | (0.051) | (0.053) | (0.054) |
| Education (ref. Tertiary) |  |  |  |
| *Primary* | -0.250*** | -0.144** | -0.148** |
|  | (0.051) | (0.049) | (0.048) |
| *Secondary* | -0.113** | -0.061+ | -0.058 |
|  | (0.037) | (0.036) | (0.035) |
| *NA* | -0.143* | 0.012 | 0.002 |
|  | (0.070) | (0.076) | (0.075) |
| Social Class of Origin (ref. Higher-grade professionals) |  |  |  |
| *Lower-grade professionals* | -0.073* | -0.052 | -0.049 |
|  | (0.034) | (0.034) | (0.034) |
| *Intermediate* | -0.152*** | -0.124** | -0.121** |
|  | (0.042) | (0.041) | (0.041) |
| *Self-employed* | -0.107** | -0.069+ | -0.066+ |
|  | (0.037) | (0.036) | (0.036) |
| *Working class* | -0.188*** | -0.136** | -0.133** |
|  | (0.041) | (0.041) | (0.041) |
| *NA* | -0.113* | -0.071 | -0.068 |
|  | (0.050) | (0.050) | (0.050) |
| Age | -0.005*** | -0.004*** | -0.004*** |
|  | (0.001) | (0.001) | (0.001) |
| Female | 0.005 | 0.018 | 0.019 |
|  | (0.022) | (0.022) | (0.022) |
| Subjective Social Status |  | 0.169*** | 0.229*** |
|  |  | (0.012) | (0.017) |
| Social Class * SSS |  |  |  |
| *Lower mgrs/professionals, higher supervisory/technicians * SSS* |  |  | -0.029+ |
|  |  |  | (0.017) |
| *Intermediate occupations * SSS* |  |  | -0.042* |
|  |  |  | (0.020) |
| *Small employers and self-employed (non-agriculture) * SSS* |  |  | -0.103*** |
|  |  |  | (0.029) |
| *Small employers and self-employed (agriculture) * SSS* |  |  | -0.061 |
|  |  |  | (0.040) |
| *Lower supervisors and technicians * SSS* |  |  | -0.054* |
|  |  |  | (0.024) |
| *Lower sales and service * SSS* |  |  | -0.039+ |
|  |  |  | (0.022) |
| *Lower technical * SSS* |  |  | -0.061** |
|  |  |  | (0.022) |
| *Routine * SSS* |  |  | -0.078*** |
|  |  |  | (0.023) |
| *NA * SSS* |  |  | -0.073*** |
|  |  |  | (0.021) |
|  |  |  |  |
| Constant | 2.675*** | 1.418*** | 1.050*** |
|  | (0.090) | (0.116) | (0.112) |
|  |  |  |  |
| **B** |  |  |  |
| Social Class (ref. Large employers, higher mgrs/professionals) |  |  |  |
| *Lower mgrs/professionals, higher supervisory/technicians* | -0.084** | -0.048+ | 0.143 |
|  | (0.026) | (0.026) | (0.108) |
| *Intermediate occupations* | -0.156*** | -0.102** | 0.170 |
|  | (0.035) | (0.036) | (0.118) |
| *Small employers and self-employed (non-agriculture)* | -0.056 | -0.017 | 0.554*** |
|  | (0.042) | (0.040) | (0.148) |
| *Small employers and self-employed (agriculture)* | 0.053 | 0.138** | 0.517** |
|  | (0.055) | (0.053) | (0.200) |
| *Lower supervisors and technicians* | -0.129** | -0.065 | 0.275+ |
|  | (0.047) | (0.047) | (0.143) |
| *Lower sales and service* | -0.162*** | -0.081* | 0.188 |
|  | (0.039) | (0.039) | (0.129) |
| *Lower technical* | -0.183*** | -0.077+ | 0.293* |
|  | (0.044) | (0.044) | (0.120) |
| *Routine* | -0.188*** | -0.091* | 0.353** |
|  | (0.041) | (0.039) | (0.124) |
| *NA* | -0.026 | -0.004 | 0.492*** |
|  | (0.034) | (0.037) | (0.110) |
| Household Income (ref. High) |  |  |  |
| *Low* | -0.303*** | -0.149*** | -0.146*** |
|  | (0.037) | (0.031) | (0.031) |
| *Middle* | -0.178*** | -0.097*** | -0.092*** |
|  | (0.024) | (0.021) | (0.021) |
| *NA* | -0.189*** | -0.132*** | -0.128*** |
|  | (0.035) | (0.035) | (0.035) |
| Education (ref. Tertiary) |  |  |  |
| *Primary* | -0.391*** | -0.293*** | -0.287*** |
|  | (0.060) | (0.057) | (0.058) |
| *Secondary* | -0.249*** | -0.199*** | -0.189*** |
|  | (0.036) | (0.034) | (0.036) |
| *NA* | -0.260*** | -0.123 | -0.125 |
|  | (0.076) | (0.081) | (0.079) |
| Social Class of Origin (ref. Higher-grade professionals) |  |  |  |
| *Lower-grade professionals* | -0.073* | -0.052 | -0.049 |
|  | (0.034) | (0.034) | (0.034) |
| *Intermediate* | -0.152*** | -0.124** | -0.121** |
|  | (0.042) | (0.041) | (0.041) |
| *Self-employed* | -0.107** | -0.069+ | -0.066+ |
|  | (0.037) | (0.036) | (0.036) |
| *Working class* | -0.232*** | -0.186*** | -0.181*** |
|  | (0.043) | (0.044) | (0.044) |
| *NA* | -0.113* | -0.071 | -0.068 |
|  | (0.050) | (0.050) | (0.050) |
| Age | -0.010*** | -0.010*** | -0.010*** |
|  | (0.001) | (0.001) | (0.001) |
| Female | -0.095*** | -0.083*** | -0.081*** |
|  | (0.019) | (0.019) | (0.019) |
| Subjective Social Status |  | 0.155*** | 0.215*** |
|  |  | (0.010) | (0.017) |
| Social Class * SSS |  |  |  |
| *Lower mgrs/professionals, higher supervisory/technicians * SSS* |  |  | -0.029+ |
|  |  |  | (0.017) |
| *Intermediate occupations * SSS* |  |  | -0.042* |
|  |  |  | (0.020) |
| *Small employers and self-employed (non-agriculture) * SSS* |  |  | -0.098*** |
|  |  |  | (0.027) |
| *Small employers and self-employed (agriculture) * SSS* |  |  | -0.061 |
|  |  |  | (0.040) |
| *Lower supervisors and technicians * SSS* |  |  | -0.054* |
|  |  |  | (0.024) |
| *Lower sales and service * SSS* |  |  | -0.046* |
|  |  |  | (0.022) |
| *Lower technical * SSS* |  |  | -0.061** |
|  |  |  | (0.022) |
| *Routine * SSS* |  |  | -0.079*** |
|  |  |  | (0.023) |
| *NA * SSS* |  |  | -0.084*** |
|  |  |  | (0.020) |
|  |  |  |  |
| Constant | 1.381*** | 0.245* | -0.136 |
|  | (0.102) | (0.101) | (0.123) |
|  |  |  |  |
| **C** |  |  |  |
| Social Class (ref. Large employers, higher mgrs/professionals) |  |  |  |
| *Lower mgrs/professionals, higher supervisory/technicians* | -0.084** | -0.048+ | 0.143 |
|  | (0.026) | (0.026) | (0.108) |
| *Intermediate occupations* | -0.156*** | -0.102** | 0.170 |
|  | (0.035) | (0.036) | (0.118) |
| *Small employers and self-employed (non-agriculture)* | -0.056 | -0.017 | 0.554*** |
|  | (0.042) | (0.040) | (0.148) |
| *Small employers and self-employed (agriculture)* | 0.053 | 0.138** | 0.517** |
|  | (0.055) | (0.053) | (0.200) |
| *Lower supervisors and technicians* | -0.129** | -0.065 | 0.275+ |
|  | (0.047) | (0.047) | (0.143) |
| *Lower sales and service* | -0.162*** | -0.081* | 0.188 |
|  | (0.039) | (0.039) | (0.129) |
| *Lower technical* | -0.183*** | -0.077+ | 0.293* |
|  | (0.044) | (0.044) | (0.120) |
| *Routine* | -0.204*** | -0.091* | 0.353** |
|  | (0.043) | (0.039) | (0.124) |
| *NA* | -0.026 | 0.031 | 0.492*** |
|  | (0.034) | (0.037) | (0.110) |
| Household Income (ref. High) |  |  |  |
| *Low* | -0.226*** | -0.084** | -0.082** |
|  | (0.033) | (0.030) | (0.030) |
| *Middle* | -0.178*** | -0.097*** | -0.092*** |
|  | (0.024) | (0.021) | (0.021) |
| *NA* | -0.114*** | -0.060+ | -0.057+ |
|  | (0.033) | (0.034) | (0.034) |
| Education (ref. Tertiary) |  |  |  |
| *Primary* | -0.123* | -0.036 | -0.033 |
|  | (0.052) | (0.049) | (0.050) |
| *Secondary* | -0.054+ | -0.011 | -0.003 |
|  | (0.032) | (0.032) | (0.032) |
| *NA* | -0.041 | 0.073 | 0.069 |
|  | (0.068) | (0.069) | (0.069) |
| Social Class of Origin (ref. Higher-grade professionals) |  |  |  |
| *Lower-grade professionals* | -0.073* | -0.052 | -0.049 |
|  | (0.034) | (0.034) | (0.034) |
| *Intermediate* | -0.152*** | -0.124** | -0.121** |
|  | (0.042) | (0.041) | (0.041) |
| *Self-employed* | -0.107** | -0.069+ | -0.066+ |
|  | (0.037) | (0.036) | (0.036) |
| *Working class* | -0.169*** | -0.122** | -0.119** |
|  | (0.044) | (0.044) | (0.044) |
| *NA* | -0.113* | -0.071 | -0.068 |
|  | (0.050) | (0.050) | (0.050) |
| Age | -0.007*** | -0.007*** | -0.007*** |
|  | (0.001) | (0.001) | (0.001) |
| Female | -0.018 | -0.008 | -0.008 |
|  | (0.018) | (0.017) | (0.018) |
| Subjective Social Status |  | 0.146*** | 0.202*** |
|  |  | (0.010) | (0.017) |
| Social Class * SSS |  |  |  |
| *Lower mgrs/professionals, higher supervisory/technicians * SSS* |  |  | -0.029+ |
|  |  |  | (0.017) |
| *Intermediate occupations * SSS* |  |  | -0.042* |
|  |  |  | (0.020) |
| *Small employers and self-employed (non-agriculture) * SSS* |  |  | -0.094*** |
|  |  |  | (0.025) |
| *Small employers and self-employed (agriculture) * SSS* |  |  | -0.061 |
|  |  |  | (0.040) |
| *Lower supervisors and technicians * SSS* |  |  | -0.054* |
|  |  |  | (0.024) |
| *Lower sales and service * SSS* |  |  | -0.039+ |
|  |  |  | (0.021) |
| *Lower technical * SSS* |  |  | -0.061** |
|  |  |  | (0.022) |
| *Routine * SSS* |  |  | -0.076*** |
|  |  |  | (0.023) |
| *NA * SSS* |  |  | -0.076*** |
|  |  |  | (0.019) |
| Constant | -0.331*** | -1.420*** | -1.784*** |
|  | (0.088) | (0.101) | (0.110) |
|  |  |  |  |
| D |  |  |  |
| Social Class (ref. Large employers, higher mgrs/professionals) |  |  |  |
| *Lower mgrs/professionals, higher supervisory/technicians* | -0.084** | -0.048+ | 0.143 |
|  | (0.026) | (0.026) | (0.108) |
| *Intermediate occupations* | -0.156*** | -0.102** | 0.170 |
|  | (0.035) | (0.036) | (0.118) |
| *Small employers and self-employed (non-agriculture)* | -0.056 | -0.017 | 0.554*** |
|  | (0.042) | (0.040) | (0.148) |
| *Small employers and self-employed (agriculture)* | 0.053 | 0.138** | 0.517** |
|  | (0.055) | (0.053) | (0.200) |
| *Lower supervisors and technicians* | -0.129** | -0.065 | 0.275+ |
|  | (0.047) | (0.047) | (0.143) |
| *Lower sales and service* | -0.162*** | -0.081* | 0.188 |
|  | (0.039) | (0.039) | (0.129) |
| *Lower technical* | -0.183*** | -0.077+ | 0.293* |
|  | (0.044) | (0.044) | (0.120) |
| *Routine* | -0.054 | -0.091* | 0.353** |
|  | (0.061) | (0.039) | (0.124) |
| *NA* | -0.026 | 0.065 | 0.492*** |
|  | (0.034) | (0.055) | (0.110) |
| Household Income (ref. High) |  |  |  |
| *Low* | 0.086+ | 0.168*** | 0.161** |
|  | (0.046) | (0.049) | (0.050) |
| *Middle* | -0.178*** | -0.097*** | -0.092*** |
|  | (0.024) | (0.021) | (0.021) |
| *NA* | 0.084 | 0.119 | 0.118 |
|  | (0.076) | (0.074) | (0.074) |
| Education (ref. Tertiary) |  |  |  |
| *Primary* | 0.457*** | 0.475*** | 0.419*** |
|  | (0.093) | (0.093) | (0.093) |
| *Secondary* | 0.175* | 0.177* | 0.140+ |
|  | (0.077) | (0.075) | (0.075) |
| *NA* | 0.541*** | 0.562*** | 0.514*** |
|  | (0.142) | (0.137) | (0.135) |
| Social Class of Origin (ref. Higher-grade professionals) |  |  |  |
| *Lower-grade professionals* | -0.073* | -0.052 | -0.049 |
|  | (0.034) | (0.034) | (0.034) |
| *Intermediate* | -0.152*** | -0.124** | -0.121** |
|  | (0.042) | (0.041) | (0.041) |
| *Self-employed* | -0.107** | -0.069+ | -0.066+ |
|  | (0.037) | (0.036) | (0.036) |
| *Working class* | 0.058 | 0.106* | 0.100+ |
|  | (0.054) | (0.053) | (0.053) |
| *NA* | -0.113* | -0.071 | -0.068 |
|  | (0.050) | (0.050) | (0.050) |
| Age | -0.007*** | -0.007*** | -0.007*** |
|  | (0.002) | (0.002) | (0.002) |
| Female | -0.082* | -0.079* | -0.089* |
|  | (0.034) | (0.035) | (0.035) |
| Subjective Social Status |  | 0.056** | 0.096*** |
|  |  | (0.019) | (0.027) |
| Social Class * SSS |  |  |  |
| *Lower mgrs/professionals, higher supervisory/technicians * SSS* |  |  | -0.029+ |
|  |  |  | (0.017) |
| *Intermediate occupations * SSS* |  |  | -0.042* |
|  |  |  | (0.020) |
| *Small employers and self-employed (non-agriculture) * SSS* |  |  | -0.067** |
|  |  |  | (0.026) |
| *Small employers and self-employed (agriculture) * SSS* |  |  | -0.061 |
|  |  |  | (0.040) |
| *Lower supervisors and technicians * SSS* |  |  | -0.054* |
|  |  |  | (0.024) |
| *Lower sales and service * SSS* |  |  | -0.003 |
|  |  |  | (0.023) |
| *Lower technical * SSS* |  |  | -0.061** |
|  |  |  | (0.022) |
| *Routine * SSS* |  |  | -0.036+ |
|  |  |  | (0.021) |
| *NA * SSS* |  |  | -0.057** |
|  |  |  | (0.020) |
|  |  |  |  |
| Constant | -4.358*** | -4.813*** | -5.114*** |
|  | (0.104) | (0.175) | (0.210) |
|  |  |  |  |
| Country-Wave Fixed Effects | *Yes* | *Yes* | *Yes* |
| BIC | 259428 | 257756 | 257875 |
| AIC | 258952 | 257232 | 257190 |

*Note*: Data from ISSP Research Group (2024), weighted. N=100,004. + p<0.10, * p<0.05, ** p<0.01, *** p<0.001

Figure A1. Predicted Probabilities of Perceived Inequality Structures by Subjective Social Status within Detailed ESeC Classes.


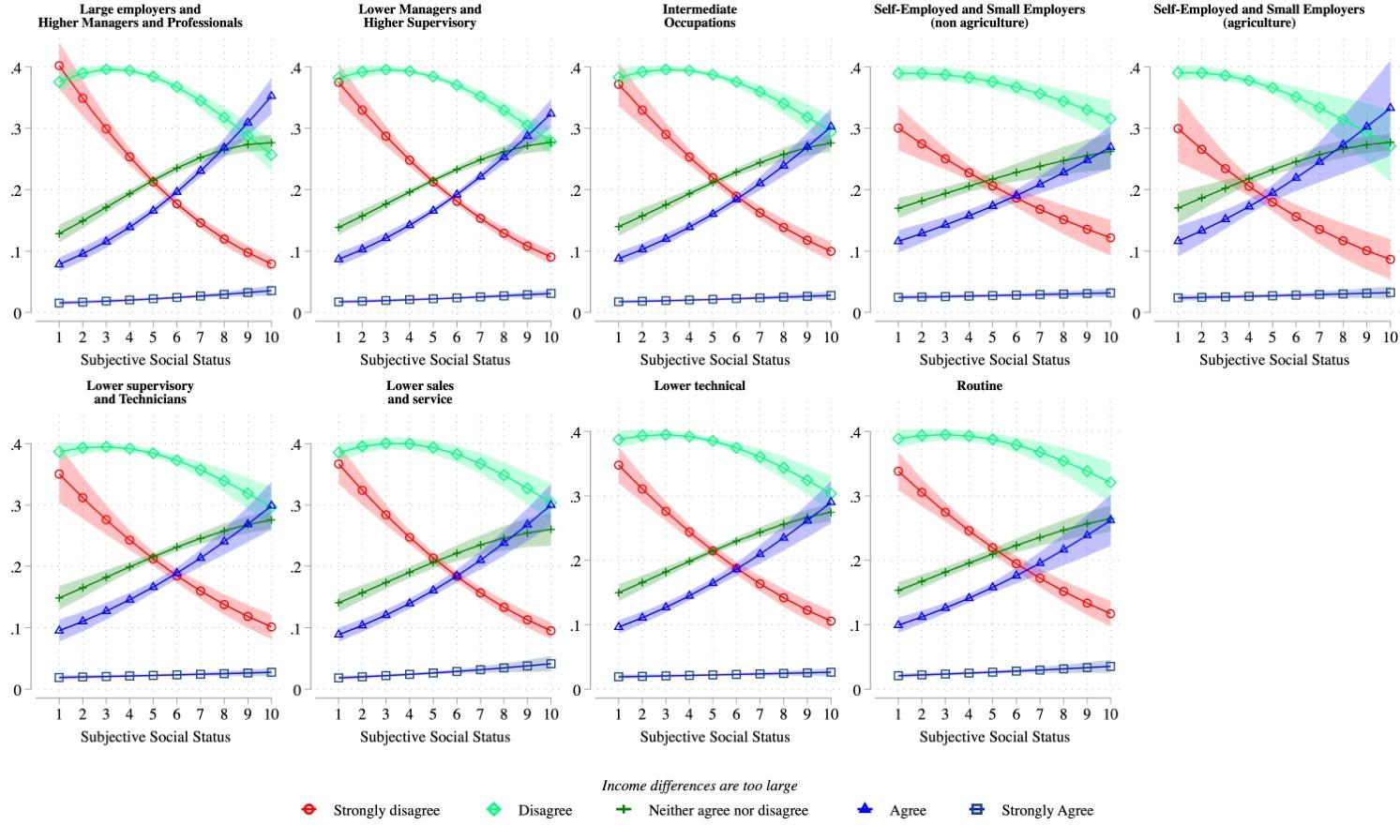


*Note*: Each panel shows predicted probabilities of selecting one of five perceived inequality structures: Type A (most unequal, many at the bottom), Types B and C (pyramid shapes with moderate inequality), Type D (most in the middle), and Type E (most near the top). Probabilities are plotted across levels of subjective social status (1–10) for each ESeC occupational class. Shaded areas represent 95% confidence intervals. Estimates are from a Generalized Ordered Logit model controlling for household income, education, social class of origin, age, gender, survey year, and country–wave fixed effects. Predictions are based on Models 3 in Table A7. Weighted ISSP data (1992, 1999, 2009, 2019).

Table A8. Mediation Analyses of the Relationship Between Social Class and Subjective Social Status via Perceived Social Structure and Between Social Class and Perceived Social Structure via Subjective Social Status.

| *Model Type* | Linear Regression Model | Linear Regression Model | Linear Regression Model | Logit Regression Model |
| --- | --- | --- | --- | --- |
| *Variable of Interest (X)* | Social Class | Social Class | Social Class | Social Class |
| *Outcome Variable (Y)* | Subjective Social Status | Perceived Social Structure | Subjective Social Status | Perceived Social Structure (A vs BCDE) |
| *Mediator Variable (Z)* | Perceived Social Structure | Subjective Social Status | Perceived Social Structure (A vs BCDE) | Subjective Social Status |
| **Social Class** |  |  |  |  |
| Higher grade professionals (ref.) |  |  |  |  |
| Lower grade professionals |  |  |  |  |
| *Reduced* | -0.243*** | -0.045*** | -0.243*** | 0.052 |
|  | (0.021) | (0.013) | (0.021) | (0.043) |
| *Full* | -0.235*** | -0.025+ | -0.241*** | 0.010 |
|  | (0.021) | (0.013) | (0.021) | (0.043) |
| *Difference* | -0.008 | -0.020*** | -0.002 | 0.042*** |
|  | (0.007) | (0.005) | (0.005) | (0.011) |
| Intermediate occupations |  |  |  |  |
| *Reduced* | -0.454*** | -0.082*** | -0.454*** | 0.143** |
|  | (0.026) | (0.016) | (0.027) | (0.044) |
| *Full* | -0.437*** | -0.044** | -0.447*** | 0.064 |
|  | (0.026) | (0.016) | (0.027) | (0.044) |
| *Difference* | -0.016* | -0.037*** | -0.007 | 0.078*** |
|  | (0.007) | (0.006) | (0.005) | (0.012) |
| Self-Employed |  |  |  |  |
| *Reduced* | -0.311*** | -0.023 | -0.311*** | 0.060 |
|  | (0.043) | (0.020) | (0.044) | (0.057) |
| *Full* | -0.305*** | 0.003 | -0.309*** | 0.006 |
|  | (0.043) | (0.020) | (0.044) | (0.057) |
| *Difference* | -0.006 | -0.026*** | -0.002 | 0.054*** |
|  | (0.007) | (0.005) | (0.005) | (0.012) |
| Working Class |  |  |  |  |
| *Reduced* | -0.658*** | -0.095*** | -0.658*** | 0.182*** |
|  | (0.026) | (0.019) | (0.026) | (0.054) |
| *Full* | -0.637*** | -0.041* | -0.647*** | 0.068 |
|  | (0.026) | (0.019) | (0.026) | (0.054) |
| *Difference* | -0.021** | -0.054*** | -0.010+ | 0.114*** |
|  | (0.007) | (0.006) | (0.005) | (0.014) |
| **Counfounding Percentages (%)** |  |  |  |  |
| Higher grade professionals (ref.) |  |  |  |  |
| Lower grade professionals | 3.41 | 45.01 | 0.71 | 80.14 |
| Intermediate occupations | 3.36 | 45.85 | 1.45 | 55.02 |
| Self-Employed | 1.93 | 112.64 | 0.55 | 89.79 |
| Working class | 3.14 | 57.05 | 1.57 | 62.50 |

*Note*: Linear and logistic regression models estimated with the KHB decomposition technique (Karlson et al., 2012). The reduced model includes only social class as predictor; the full model adds the mediator (either subjective social status or perceived social structure). The “difference” represents the indirect effect through the mediator. Coefficients are shown for each social class relative to higher-grade professionals. Confounding percentages report the share of the total effect explained by mediation; values above 100 indicate suppression, where the mediator reverses or exceeds the total effect. Standard errors in parentheses. Models control for household income terciles, education, social class background, age, gender, survey year, and country–wave fixed effects. Weighted ISSP data (1992, 1999, 2009, 2019). N=100,004. + p<0.10, * p<0.05, ** p<0.01, *** p<0.001

Table A9. Results of Linear Regression Models for Income Differences are Too Large.

|  | (1) | (2) | (3) | (4) | (5) | (6) |
| --- | --- | --- | --- | --- | --- | --- |
| Social Class (ref. Higher-grade professionals) |  |  |  |  |  |  |
| *Lower-grade professionals* | 0.174*** | 0.159*** | -0.140* | 0.166*** | 0.154*** | -0.131* |
|  | (0.022) | (0.021) | (0.057) | (0.021) | (0.021) | (0.055) |
| *Intermediate* | 0.179*** | 0.153*** | -0.213*** | 0.166*** | 0.145*** | -0.201** |
|  | (0.024) | (0.023) | (0.063) | (0.023) | (0.022) | (0.061) |
| *Self-employed* | 0.077** | 0.060* | -0.284** | 0.072* | 0.058* | -0.248** |
|  | (0.028) | (0.027) | (0.091) | (0.027) | (0.027) | (0.089) |
| *Working class* | 0.197*** | 0.160*** | -0.328*** | 0.182*** | 0.152*** | -0.306*** |
|  | (0.027) | (0.026) | (0.066) | (0.026) | (0.024) | (0.063) |
| *NA* | 0.086** | 0.064* | -0.343*** | 0.080** | 0.061* | -0.314*** |
|  | (0.028) | (0.027) | (0.065) | (0.027) | (0.026) | (0.063) |
| Subjective Social Status |  | -0.060*** | -0.122*** |  | -0.051*** | -0.108*** |
|  |  | (0.006) | (0.012) |  | (0.005) | (0.012) |
| Social Class * SSS |  |  |  |  |  |  |
| *Lower-grade professionals * SSS* |  |  | 0.047*** |  |  | 0.045*** |
|  |  |  | (0.011) |  |  | (0.010) |
| *Intermediate * SSS* |  |  | 0.059*** |  |  | 0.056*** |
|  |  |  | (0.012) |  |  | (0.012) |
| *Self-employed * SSS* |  |  | 0.054** |  |  | 0.048* |
|  |  |  | (0.019) |  |  | (0.019) |
| *Working class * SSS* |  |  | 0.085*** |  |  | 0.080*** |
|  |  |  | (0.013) |  |  | (0.012) |
| *NA * SSS* |  |  | 0.067*** |  |  | 0.062*** |
|  |  |  | (0.013) |  |  | (0.012) |
| Perceived Social Structure (ref. B) |  |  |  |  |  |  |
| *A* |  |  |  | 0.120*** | 0.105*** | 0.106*** |
|  |  |  |  | (0.012) | (0.011) | (0.011) |
| *C* |  |  |  | -0.153*** | -0.144*** | -0.143*** |
|  |  |  |  | (0.013) | (0.013) | (0.013) |
| *D* |  |  |  | -0.310*** | -0.294*** | -0.291*** |
|  |  |  |  | (0.022) | (0.021) | (0.021) |
| *E* |  |  |  | -0.127*** | -0.120*** | -0.120*** |
|  |  |  |  | (0.031) | (0.030) | (0.029) |
| Household Income (ref. High) |  |  |  |  |  |  |
| *Low* | 0.182*** | 0.120*** | 0.117*** | 0.160*** | 0.110*** | 0.107*** |
|  | (0.020) | (0.016) | (0.016) | (0.017) | (0.015) | (0.015) |
| *Middle* | 0.138*** | 0.105*** | 0.100*** | 0.125*** | 0.099*** | 0.094*** |
|  | (0.016) | (0.015) | (0.014) | (0.015) | (0.014) | (0.014) |
| *NA* | 0.088*** | 0.067*** | 0.063*** | 0.075*** | 0.059*** | 0.055*** |
|  | (0.017) | (0.016) | (0.016) | (0.016) | (0.015) | (0.015) |
| Education (ref. Tertiary) |  |  |  |  |  |  |
| *Primary* | 0.093*** | 0.053* | 0.050* | 0.071*** | 0.039* | 0.036+ |
|  | (0.023) | (0.021) | (0.020) | (0.021) | (0.019) | (0.019) |
| *Secondary* | 0.044* | 0.024 | 0.017 | 0.033+ | 0.017 | 0.010 |
|  | (0.019) | (0.018) | (0.017) | (0.017) | (0.017) | (0.017) |
| *NA* | -0.006 | -0.062** | -0.061** | -0.022 | -0.068*** | -0.067*** |
|  | (0.022) | (0.021) | (0.021) | (0.020) | (0.020) | (0.020) |
| Social Class of Origin (ref. Higher-grade professionals) |  |  |  |  |  |  |
| *Lower-grade professionals* | 0.069*** | 0.063*** | 0.060*** | 0.065*** | 0.060*** | 0.057*** |
|  | (0.015) | (0.015) | (0.015) | (0.015) | (0.015) | (0.015) |
| *Intermediate* | 0.092*** | 0.082*** | 0.078*** | 0.081*** | 0.074*** | 0.070*** |
|  | (0.020) | (0.019) | (0.019) | (0.018) | (0.018) | (0.018) |
| *Self-employed* | 0.072*** | 0.057** | 0.053** | 0.065** | 0.053** | 0.049* |
|  | (0.020) | (0.020) | (0.020) | (0.019) | (0.019) | (0.019) |
| *Working class* | 0.122*** | 0.104*** | 0.100*** | 0.106*** | 0.092*** | 0.089*** |
|  | (0.018) | (0.018) | (0.017) | (0.017) | (0.017) | (0.016) |
| *NA* | 0.034 | 0.018 | 0.014 | 0.024 | 0.010 | 0.007 |
|  | (0.026) | (0.025) | (0.025) | (0.023) | (0.023) | (0.023) |
| Age | 0.003*** | 0.003*** | 0.003*** | 0.003*** | 0.003*** | 0.003*** |
|  | (0.000) | (0.000) | (0.000) | (0.000) | (0.000) | (0.000) |
| Female | 0.100*** | 0.094*** | 0.093*** | 0.097*** | 0.092*** | 0.091*** |
|  | (0.014) | (0.013) | (0.013) | (0.014) | (0.013) | (0.013) |
|  |  |  |  |  |  |  |
| Constant | 3.586*** | 3.993*** | 4.382*** | 3.714*** | 4.050*** | 4.411*** |
|  | (0.061) | (0.051) | (0.060) | (0.053) | (0.046) | (0.057) |
|  |  |  |  |  |  |  |
| Observations | 98649 | 98649 | 98649 | 98649 | 98649 | 98649 |
| BIC | 256145 | 255058 | 254930 | 253922 | 253153 | 253044 |
| AIC | 255974 | 254877 | 254702 | 253713 | 252934 | 252778 |

*Note*: Data from ISSP Research Group (2024), weighted. + p<0.10, * p<0.05, ** p<0.01, *** p<0.001

Table A10. Mediation Analysis of the Relationship Between Subjective Social Status and Redistribution Preferences via Perceived Social Structure.

| *Model Type* | Linear Regression Model | Linear Regression Model |
| --- | --- | --- |
| *Variable of Interest (X)* | Subjective Social Status | Subjective Social Status |
| *Outcome Variable (Y)* | **Government should redistribute income** | **Income differences are too large** |
| *Mediator Variable (Z)* | Perceived Social Structure | Perceived Social Structure |
| **Subjective Social Status** |  |  |
| *Reduced* | -0.073*** | -0.065*** |
|  | (0.005) | (0.005) |
| *Full* | -0.065*** | -0.055*** |
|  | (0.005) | (0.004) |
| *Difference* | -0.009*** | -0.009*** |
|  | (0.001) | (0.001) |
|  |  |  |
| Confounding Percentage (%) | 11.97 | 14.59 |
| Observations | 78669 | 79204 |

*Note*: Linear regression models estimated with the KHB decomposition technique (Karlson et al., 2012). The reduced model includes subjective social status as predictor of redistribution preferences; the full model adds perceived social structure as mediator. The “Difference” shows the indirect effect of subjective status operating through perceived social structure. Results are shown for two outcome variables: agreement that government should redistribute income and agreement that income differences are too large. Confounding percentage indicates the share of the total effect mediated by perceived social structure. Standard errors in parentheses. Models control for social class, household income terciles, education, social class background, age, gender, survey year, and country–wave fixed effects. Weighted ISSP data (1992, 1999, 2009, 2019). N=100,004. + p<0.10, * p<0.05, ** p<0.01, *** p<0.001

Figure A2. Predicted Support for the Statement “Income Differences Are Too Large” by Social Class and Subjective Social Status, Without and With Perceived Social Structure


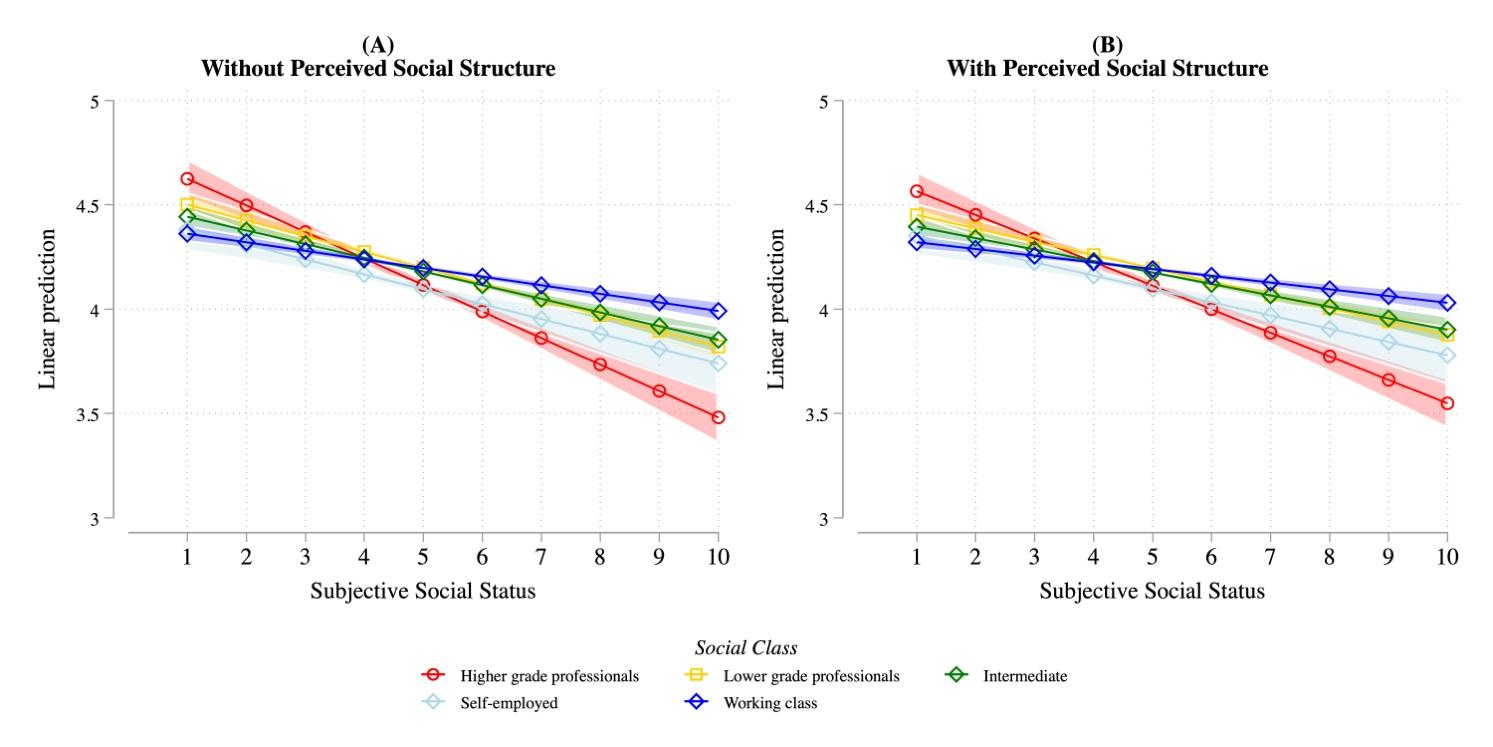


*Note*: Panels show predicted linear responses for higher-grade professionals, intermediate occupations, and working-class respondents across levels of subjective social status (1–10). Panel A reports results without including perceived social structure; Panel B includes perceived social structure as mediator. Shaded areas represent 95% confidence intervals. Estimates are from linear regression models controlling for household income terciles, education, social class of origin, age, gender, survey year, and country–wave fixed effects. Predictions are based on Models 3 and 6 in Table A9. Weighted ISSP data (1992, 1999, 2009, 2019).

Table A11. Results of Generalised Ordered Logit Models for Ordinal Dependent Variables for Income Differences are Too Large as a Function of Social Class and Subjective Social Status.

| **Strongly disagree** |  |
| --- | --- |
| Perceived Social Structure (ref. B) |  |
| *A* | 0.327*** |
|  | (0.038) |
| *C* | -0.055 |
|  | (0.091) |
| *D* | -0.537*** |
|  | (0.090) |
| *E* | -0.669*** |
|  | (0.123) |
| Social Class (ref. Higher-grade professionals) |  |
| *Lower-grade professionals* | 0.247 |
|  | (0.169) |
| *Intermediate* | -0.139 |
|  | (0.136) |
| *Self-employed* | -0.495** |
|  | (0.181) |
| *Working class* | -1.155*** |
|  | (0.228) |
| *NA* | -1.015** |
|  | (0.350) |
| Subjective Social Status | -0.264*** |
|  | (0.031) |
| Social Class * SSS |  |
| *Lower-grade professionals * SSS* | 0.054* |
|  | (0.022) |
| *Intermediate * SSS* | 0.111*** |
|  | (0.026) |
| *Self-employed * SSS* | 0.088* |
|  | (0.035) |
| *Working class * SSS* | 0.276*** |
|  | (0.039) |
| *NA * SSS* | 0.218*** |
|  | (0.059) |
| Household Income (ref. High) |  |
| *Low* | 0.231*** |
|  | (0.032) |
| *Middle* | 0.182*** |
|  | (0.028) |
| *NA* | 0.092 |
|  | (0.091) |
| Education (ref. Tertiary) |  |
| *Primary* | 0.233* |
|  | (0.091) |
| *Secondary* | 0.238* |
|  | (0.105) |
| *NA* | -0.088 |
|  | (0.160) |
| Social Class of Origin (ref. Higher-grade professionals) |  |
| *Lower-grade professionals* | 0.127*** |
|  | (0.033) |
| *Intermediate* | 0.129** |
|  | (0.040) |
| *Self-employed* | 0.084* |
|  | (0.039) |
| *Working class* | 0.258** |
|  | (0.099) |
| *NA* | -0.043 |
|  | (0.127) |
| Age | 0.008*** |
|  | (0.001) |
| Female | 0.354*** |
|  | (0.068) |
|  |  |
| Constant | 4.396*** |
|  | (0.231) |
|  |  |
| **Disagree** |  |
| Perceived Social Structure (ref. B) |  |
| *A* | 0.327*** |
|  | (0.038) |
| *C* | -0.253*** |
|  | (0.056) |
| *D* | -0.613*** |
|  | (0.061) |
| *E* | -0.475*** |
|  | (0.082) |
| Social Class (ref. Higher-grade professionals) |  |
| *Lower-grade professionals* | 0.043 |
|  | (0.149) |
| *Intermediate* | -0.139 |
|  | (0.136) |
| *Self-employed* | -0.495** |
|  | (0.181) |
| *Working class* | -0.753*** |
|  | (0.159) |
| *NA* | -0.599** |
|  | (0.213) |
| Subjective Social Status | -0.269*** |
|  | (0.023) |
| Social Class * SSS |  |
| *Lower-grade professionals * SSS* | 0.054* |
|  | (0.022) |
| *Intermediate * SSS* | 0.082** |
|  | (0.025) |
| *Self-employed * SSS* | 0.088* |
|  | (0.035) |
| *Working class * SSS* | 0.194*** |
|  | (0.028) |
| *NA * SSS* | 0.129*** |
|  | (0.036) |
| Household Income (ref. High) |  |
| *Low* | 0.231*** |
|  | (0.032) |
| *Middle* | 0.182*** |
|  | (0.028) |
| *NA* | 0.165** |
|  | (0.057) |
| Education (ref. Tertiary) |  |
| *Primary* | 0.203*** |
|  | (0.060) |
| *Secondary* | 0.123* |
|  | (0.054) |
| *NA* | 0.034 |
|  | (0.107) |
| Social Class of Origin (ref. Higher-grade professionals) |  |
| *Lower-grade professionals* | 0.127*** |
|  | (0.033) |
| *Intermediate* | 0.129** |
|  | (0.040) |
| *Self-employed* | 0.084* |
|  | (0.039) |
| *Working class* | 0.268*** |
|  | (0.045) |
| *NA* | -0.004 |
|  | (0.063) |
| Age | 0.008*** |
|  | (0.001) |
| Female | 0.305*** |
|  | (0.051) |
|  |  |
| Constant | 2.123*** |
|  | (0.148) |
|  |  |
| **Neither agree nor disagree** |  |
| Perceived Social Structure (ref. B) |  |
| *A* | 0.327*** |
|  | (0.038) |
| *C* | -0.348*** |
|  | (0.042) |
| *D* | -0.625*** |
|  | (0.052) |
| *E* | -0.296*** |
|  | (0.062) |
| Social Class (ref. Higher-grade professionals) |  |
| *Lower-grade professionals* | -0.034 |
|  | (0.138) |
| *Intermediate* | -0.139 |
|  | (0.136) |
| *Self-employed* | -0.495** |
|  | (0.181) |
| *Working class* | -0.509*** |
|  | (0.146) |
| *NA* | -0.589** |
|  | (0.180) |
| Subjective Social Status | -0.246*** |
|  | (0.019) |
| Social Class * SSS |  |
| *Lower-grade professionals * SSS* | 0.054* |
|  | (0.022) |
| *Intermediate * SSS* | 0.063* |
|  | (0.025) |
| *Self-employed * SSS* | 0.088* |
|  | (0.035) |
| *Working class * SSS* | 0.144*** |
|  | (0.025) |
| *NA * SSS* | 0.108*** |
|  | (0.033) |
| Household Income (ref. High) |  |
| *Low* | 0.231*** |
|  | (0.032) |
| *Middle* | 0.182*** |
|  | (0.028) |
| *NA* | 0.037 |
|  | (0.042) |
| Education (ref. Tertiary) |  |
| *Primary* | 0.131* |
|  | (0.053) |
| *Secondary* | 0.043 |
|  | (0.040) |
| *NA* | -0.162** |
|  | (0.057) |
| Social Class of Origin (ref. Higher-grade professionals) |  |
| *Lower-grade professionals* | 0.127*** |
|  | (0.033) |
| *Intermediate* | 0.129** |
|  | (0.040) |
| *Self-employed* | 0.084* |
|  | (0.039) |
| *Working class* | 0.210*** |
|  | (0.040) |
| *NA* | -0.035 |
|  | (0.059) |
| Age | 0.008*** |
|  | (0.001) |
| Female | 0.276*** |
|  | (0.041) |
|  |  |
| Constant | 1.193*** |
|  | (0.137) |
|  |  |
| **Agree** |  |
| Perceived Social Structure (ref. B) |  |
| *A* | 0.327*** |
|  | (0.038) |
| *C* | -0.338*** |
|  | (0.033) |
| *D* | -0.537*** |
|  | (0.040) |
| *E* | 0.009 |
|  | (0.066) |
| Social Class (ref. Higher-grade professionals) |  |
| *Lower-grade professionals* | -0.188 |
|  | (0.128) |
| *Intermediate* | -0.139 |
|  | (0.136) |
| *Self-employed* | -0.495** |
|  | (0.181) |
| *Working class* | -0.295* |
|  | (0.134) |
| *NA* | -0.471*** |
|  | (0.138) |
| Subjective Social Status | -0.180*** |
|  | (0.021) |
| Social Class * SSS |  |
| *Lower-grade professionals * SSS* | 0.054* |
|  | (0.022) |
| *Intermediate * SSS* | 0.041 |
|  | (0.025) |
| *Self-employed * SSS* | 0.088* |
|  | (0.035) |
| *Working class * SSS* | 0.080*** |
|  | (0.024) |
| *NA * SSS* | 0.072** |
|  | (0.024) |
| Household Income (ref. High) |  |
| *Low* | 0.231*** |
|  | (0.032) |
| *Middle* | 0.182*** |
|  | (0.028) |
| *NA* | 0.070+ |
|  | (0.039) |
| Education (ref. Tertiary) |  |
| *Primary* | 0.039 |
|  | (0.045) |
| *Secondary* | -0.032 |
|  | (0.038) |
| *NA* | -0.253*** |
|  | (0.071) |
| Social Class of Origin (ref. Higher-grade professionals) |  |
| *Lower-grade professionals* | 0.127*** |
|  | (0.033) |
| *Intermediate* | 0.129** |
|  | (0.040) |
| *Self-employed* | 0.084* |
|  | (0.039) |
| *Working class* | 0.163*** |
|  | (0.041) |
| *NA* | -0.014 |
|  | (0.061) |
| Age | 0.008*** |
|  | (0.001) |
| Female | 0.139*** |
|  | (0.023) |
|  |  |
|  |  |
| Constant | -1.137*** |
|  | (0.137) |
|  |  |
| Country-Wave Fixed Effects | *Yes* |
| BIC | 215842 |
| AIC | 214930 |

*Note*: Data from ISSP Research Group (2024), weighted. + p<0.10, * p<0.05, ** p<0.01, *** p<0.001

Figure A3. Predicted Agreement with the Statement “Income Differences Are Too Large” by Subjective Social Status within Social Classes.


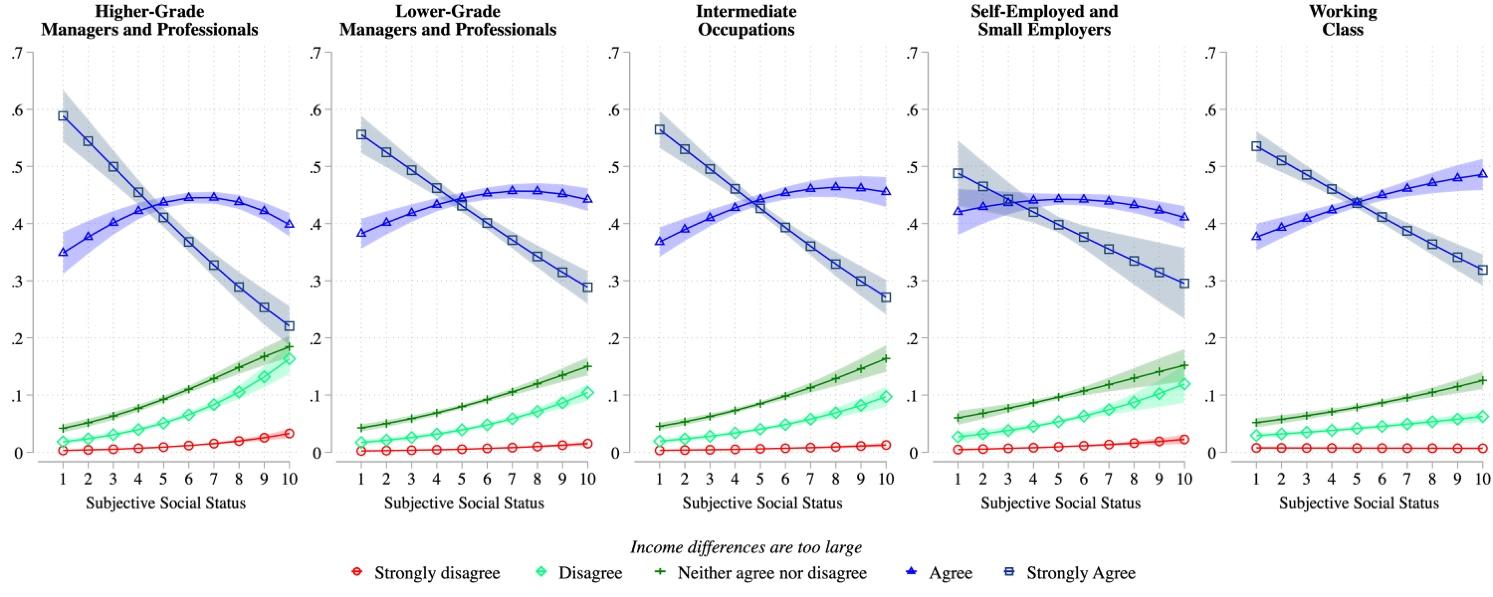


*Note*: Each panel shows predicted probabilities of response categories (strongly disagree to strongly agree) across levels of subjective social status (1–10), separately for higher-grade professionals, lower-grade professionals, intermediate occupations, self-employed, and working-class respondents. Shaded areas represent 95% confidence intervals. Estimates are from linear regression models controlling for household income terciles, education, social class of origin, age, gender, survey year, and country–wave fixed effects. Predictions are based on Model 1 in Table A11. Weighted ISSP data (1992, 1999, 2009, 2019).

Table A12. Results of Generalised Ordered Logit Models for Ordinal Dependent Variables for Perceived Social Structure, List Wise Deletion of Missing Values.

|  | (1) | (2) | (3) |
| --- | --- | --- | --- |
|  | M1 | M2 | M3 |
|  |  |  |  |
| **A** |  |  |  |
| Social Class (ref. Higher-grade professionals) |  |  |  |
| *Lower-grade professionals* | -0.109*** | -0.066* | 0.074 |
|  | (0.030) | (0.030) | (0.129) |
| *Intermediate* | -0.163*** | -0.084* | 0.214+ |
|  | (0.040) | (0.040) | (0.125) |
| *Self-employed* | -0.035 | 0.023 | 0.557*** |
|  | (0.049) | (0.046) | (0.163) |
| *Working class* | -0.166*** | -0.058 | 0.363** |
|  | (0.042) | (0.041) | (0.123) |
| Household Income (ref. High) |  |  |  |
| *Low* | -0.312*** | -0.139*** | -0.141*** |
|  | (0.045) | (0.040) | (0.040) |
| *Middle* | -0.141*** | -0.052 | -0.049 |
|  | (0.035) | (0.033) | (0.033) |
| Education (ref. Tertiary) |  |  |  |
| *Primary* | -0.288*** | -0.193*** | -0.202*** |
|  | (0.060) | (0.056) | (0.056) |
| *Secondary* | -0.131** | -0.083* | -0.081+ |
|  | (0.044) | (0.042) | (0.042) |
| Social Class of Origin (ref. Higher-grade professionals) |  |  |  |
| *Lower-grade professionals* | -0.089* | -0.073* | -0.068+ |
|  | (0.036) | (0.035) | (0.035) |
| *Intermediate* | -0.169*** | -0.142*** | -0.138*** |
|  | (0.041) | (0.040) | (0.040) |
| *Self-employed* | -0.112*** | -0.070* | -0.067* |
|  | (0.034) | (0.032) | (0.032) |
| *Working class* | -0.175*** | -0.130** | -0.126** |
|  | (0.042) | (0.044) | (0.043) |
| Age | -0.004** | -0.003** | -0.004** |
|  | (0.001) | (0.001) | (0.001) |
| Female | -0.005 | 0.009 | 0.011 |
|  | (0.025) | (0.026) | (0.026) |
| Subjective Social Status |  | 0.175*** | 0.228*** |
|  |  | (0.013) | (0.017) |
| Social Class * SSS |  |  |  |
| *Lower-grade professionals * SSS* |  |  | -0.020 |
|  |  |  | (0.020) |
| *Intermediate * SSS* |  |  | -0.047* |
|  |  |  | (0.020) |
| *Self-employed * SSS* |  |  | -0.091** |
|  |  |  | (0.031) |
| *Working class * SSS* |  |  | -0.072** |
|  |  |  | (0.022) |
|  |  |  |  |
| Constant | 2.764*** | 1.458*** | 1.134*** |
|  | (0.097) | (0.139) | (0.136) |
|  |  |  |  |
| **B** |  |  |  |
| Social Class (ref. Higher-grade professionals) |  |  |  |
| *Lower-grade professionals* | -0.109*** | -0.066* | 0.074 |
|  | (0.030) | (0.030) | (0.129) |
| *Intermediate* | -0.163*** | -0.084* | 0.214+ |
|  | (0.040) | (0.040) | (0.125) |
| *Self-employed* | -0.035 | 0.023 | 0.557*** |
|  | (0.049) | (0.046) | (0.163) |
| *Working class* | -0.166*** | -0.058 | 0.363** |
|  | (0.042) | (0.041) | (0.123) |
| Household Income (ref. High) |  |  |  |
| *Low* | -0.299*** | -0.128*** | -0.126*** |
|  | (0.044) | (0.036) | (0.037) |
| *Middle* | -0.158*** | -0.068* | -0.063+ |
|  | (0.036) | (0.032) | (0.033) |
| Education (ref. Tertiary) |  |  |  |
| *Primary* | -0.401*** | -0.308*** | -0.305*** |
|  | (0.074) | (0.068) | (0.068) |
| *Secondary* | -0.260*** | -0.210*** | -0.201*** |
|  | (0.039) | (0.036) | (0.037) |
| Social Class of Origin (ref. Higher-grade professionals) |  |  |  |
| *Lower-grade professionals* | -0.089* | -0.073* | -0.068+ |
|  | (0.036) | (0.035) | (0.035) |
| *Intermediate* | -0.169*** | -0.142*** | -0.138*** |
|  | (0.041) | (0.040) | (0.040) |
| *Self-employed* | -0.112*** | -0.070* | -0.067* |
|  | (0.034) | (0.032) | (0.032) |
| *Working class* | -0.235*** | -0.190*** | -0.185*** |
|  | (0.041) | (0.040) | (0.039) |
| Age | -0.010*** | -0.010*** | -0.010*** |
|  | (0.001) | (0.001) | (0.001) |
| Female | -0.099*** | -0.089*** | -0.088*** |
|  | (0.023) | (0.024) | (0.024) |
| Subjective Social Status |  | 0.175*** | 0.228*** |
|  |  | (0.013) | (0.017) |
| Social Class * SSS |  |  |  |
| *Lower-grade professionals * SSS* |  |  | -0.020 |
|  |  |  | (0.020) |
| *Intermediate * SSS* |  |  | -0.047* |
|  |  |  | (0.020) |
| *Self-employed * SSS* |  |  | -0.091** |
|  |  |  | (0.031) |
| *Working class * SSS* |  |  | -0.072** |
|  |  |  | (0.022) |
|  |  |  |  |
| Constant | 1.450*** | 0.165 | -0.170 |
|  | (0.111) | (0.116) | (0.135) |
|  |  |  |  |
| **C** |  |  |  |
| Social Class (ref. Higher-grade professionals) |  |  |  |
| *Lower-grade professionals* | -0.109*** | -0.066* | 0.074 |
|  | (0.030) | (0.030) | (0.129) |
| *Intermediate* | -0.163*** | -0.084* | 0.214+ |
|  | (0.040) | (0.040) | (0.125) |
| *Self-employed* | -0.035 | 0.023 | 0.557*** |
|  | (0.049) | (0.046) | (0.163) |
| *Working class* | -0.166*** | -0.058 | 0.363** |
|  | (0.042) | (0.041) | (0.123) |
| Household Income (ref. High) |  |  |  |
| *Low* | -0.242*** | -0.075* | -0.070* |
|  | (0.038) | (0.036) | (0.036) |
| *Middle* | -0.176*** | -0.086** | -0.078** |
|  | (0.031) | (0.029) | (0.029) |
| Education (ref. Tertiary) |  |  |  |
| *Primary* | -0.170** | -0.074 | -0.064 |
|  | (0.057) | (0.053) | (0.054) |
| *Secondary* | -0.056+ | -0.005 | 0.009 |
|  | (0.034) | (0.032) | (0.032) |
| Social Class of Origin (ref. Higher-grade professionals) |  |  |  |
| *Lower-grade professionals* | -0.089* | -0.073* | -0.068+ |
|  | (0.036) | (0.035) | (0.035) |
| *Intermediate* | -0.169*** | -0.142*** | -0.138*** |
|  | (0.041) | (0.040) | (0.040) |
| *Self-employed* | -0.112*** | -0.070* | -0.067* |
|  | (0.034) | (0.032) | (0.032) |
| *Working class* | -0.192*** | -0.145*** | -0.139*** |
|  | (0.042) | (0.041) | (0.041) |
| Age | -0.007*** | -0.007*** | -0.007*** |
|  | (0.001) | (0.001) | (0.001) |
| Female | -0.023 | -0.013 | -0.013 |
|  | (0.024) | (0.024) | (0.024) |
| Subjective Social Status |  | 0.175*** | 0.228*** |
|  |  | (0.013) | (0.017) |
| Social Class * SSS |  |  |  |
| *Lower-grade professionals * SSS* |  |  | -0.020 |
|  |  |  | (0.020) |
| *Intermediate * SSS* |  |  | -0.047* |
|  |  |  | (0.020) |
| *Self-employed * SSS* |  |  | -0.091** |
|  |  |  | (0.031) |
| *Working class * SSS* |  |  | -0.072** |
|  |  |  | (0.022) |
| Constant | -0.259* | -1.548*** | -1.889*** |
|  | (0.102) | (0.129) | (0.126) |
|  |  |  |  |
| **D** |  |  |  |
| Social Class (ref. Higher-grade professionals) |  |  |  |
| *Lower-grade professionals* | -0.109*** | -0.066* | 0.074 |
|  | (0.030) | (0.030) | (0.129) |
| *Intermediate* | -0.163*** | -0.084* | 0.214+ |
|  | (0.040) | (0.040) | (0.125) |
| *Self-employed* | -0.035 | 0.023 | 0.557*** |
|  | (0.049) | (0.046) | (0.163) |
| *Working class* | -0.166*** | -0.058 | 0.363** |
|  | (0.042) | (0.041) | (0.123) |
| Household Income (ref. High) |  |  |  |
| *Low* | 0.232*** | 0.397*** | 0.403*** |
|  | (0.068) | (0.071) | (0.071) |
| *Middle* | 0.048 | 0.141+ | 0.149* |
|  | (0.073) | (0.075) | (0.074) |
| Education (ref. Tertiary) |  |  |  |
| *Primary* | 0.411*** | 0.505*** | 0.519*** |
|  | (0.093) | (0.098) | (0.098) |
| *Secondary* | 0.152 | 0.199* | 0.217* |
|  | (0.093) | (0.094) | (0.094) |
| Social Class of Origin (ref. Higher-grade professionals) |  |  |  |
| *Lower-grade professionals* | -0.089* | -0.073* | -0.068+ |
|  | (0.036) | (0.035) | (0.035) |
| *Intermediate* | -0.169*** | -0.142*** | -0.138*** |
|  | (0.041) | (0.040) | (0.040) |
| *Self-employed* | -0.112*** | -0.070* | -0.067* |
|  | (0.034) | (0.032) | (0.032) |
| *Working class* | 0.033 | 0.082 | 0.087 |
|  | (0.060) | (0.060) | (0.060) |
| Age | -0.011*** | -0.011*** | -0.011*** |
|  | (0.003) | (0.003) | (0.003) |
| Female | -0.116* | -0.105+ | -0.106+ |
|  | (0.056) | (0.056) | (0.056) |
| Subjective Social Status |  | 0.175*** | 0.228*** |
|  |  | (0.013) | (0.017) |
| Social Class * SSS |  |  |  |
| *Lower-grade professionals * SSS* |  |  | -0.020 |
|  |  |  | (0.020) |
| *Intermediate * SSS* |  |  | -0.047* |
|  |  |  | (0.020) |
| *Self-employed * SSS* |  |  | -0.091** |
|  |  |  | (0.031) |
| *Working class * SSS* |  |  | -0.072** |
|  |  |  | (0.022) |
|  |  |  |  |
| Constant | -4.565*** | -5.870*** | -6.213*** |
|  | (0.146) | (0.180) | (0.174) |
|  |  |  |  |
| Country-Waves Fixed Effects | *Yes* | *Yes* | *Yes* |
| Observations | 56042 | 56042 | 56042 |
| BIC | 144372 | 143070 | 143069 |
| AIC | 143640 | 142561 | 142533 |

*Note*: Data from ISSP Research Group (2024), weighted. N=100,004. + p<0.10, * p<0.05, ** p<0.01, *** p<0.001

Table A13. Results of Generalised Ordered Logit Models for Ordinal Dependent Variables for Perceived Social Structure, without Survey Weights.

|  | (1) | (2) | (3) |
| --- | --- | --- | --- |
|  |  |  |  |
| **A** |  |  |  |
| Social Class (ref. Higher-grade professionals) |  |  |  |
| *Lower-grade professionals* | -0.101*** | -0.066** | 0.136 |
|  | (0.025) | (0.025) | (0.102) |
| *Intermediate* | -0.195*** | -0.130*** | 0.155 |
|  | (0.036) | (0.036) | (0.113) |
| *Self-employed* | -0.074+ | -0.029 | 0.571*** |
|  | (0.042) | (0.043) | (0.136) |
| *Working class* | -0.215*** | -0.120** | 0.311** |
|  | (0.043) | (0.043) | (0.110) |
| *NA* | -0.048 | 0.011 | 0.472*** |
|  | (0.041) | (0.040) | (0.105) |
| Subjective Social Status |  | 0.163*** | 0.227*** |
|  |  | (0.011) | (0.019) |
| Social Class * SSS |  |  |  |
| *Lower-grade professionals * SSS* |  |  | -0.029 |
|  |  |  | (0.018) |
| *Intermediate * SSS* |  |  | -0.044* |
|  |  |  | (0.022) |
| *Self-employed * SSS* |  |  | -0.109*** |
|  |  |  | (0.027) |
| *Working class * SSS* |  |  | -0.074*** |
|  |  |  | (0.022) |
| *NA * SSS* |  |  | -0.080*** |
|  |  |  | (0.022) |
| Household Income (ref. High) |  |  |  |
| *Low* | -0.337*** | -0.167*** | -0.165*** |
|  | (0.039) | (0.032) | (0.031) |
| *Middle* | -0.194*** | -0.104*** | -0.099*** |
|  | (0.028) | (0.019) | (0.019) |
| *NA* | -0.183*** | -0.133*** | -0.129** |
|  | (0.039) | (0.040) | (0.039) |
| Education (ref. Tertiary) |  |  |  |
| *Primary* | -0.234*** | -0.126** | -0.126** |
|  | (0.051) | (0.048) | (0.047) |
| *Secondary* | -0.106** | -0.053 | -0.046 |
|  | (0.036) | (0.034) | (0.033) |
| *NA* | -0.133* | 0.026 | 0.016 |
|  | (0.060) | (0.060) | (0.060) |
| Social Class of Origin (ref. Higher-grade professionals) |  |  |  |
| *Lower-grade professionals* | -0.051+ | -0.037 | -0.033 |
|  | (0.030) | (0.031) | (0.031) |
| *Intermediate* | -0.143*** | -0.121*** | -0.117*** |
|  | (0.035) | (0.034) | (0.034) |
| *Self-employed* | -0.085** | -0.050 | -0.047 |
|  | (0.033) | (0.032) | (0.032) |
| *Working class* | -0.184*** | -0.140*** | -0.136*** |
|  | (0.037) | (0.037) | (0.037) |
| *NA* | -0.112* | -0.073 | -0.069 |
|  | (0.046) | (0.047) | (0.046) |
| Age | -0.004*** | -0.004*** | -0.004*** |
|  | (0.001) | (0.001) | (0.001) |
| Female | -0.014 | 0.002 | 0.002 |
|  | (0.019) | (0.018) | (0.018) |
|  |  |  |  |
| Constant | 2.667*** | 1.461*** | 1.063*** |
|  | (0.091) | (0.103) | (0.105) |
|  |  |  |  |
| **B** |  |  |  |
| Social Class (ref. Higher-grade professionals) |  |  |  |
| *Lower-grade professionals* | -0.101*** | -0.066** | 0.087 |
|  | (0.025) | (0.025) | (0.112) |
| *Intermediate* | -0.196*** | -0.133*** | 0.155 |
|  | (0.036) | (0.036) | (0.113) |
| *Self-employed* | -0.053 | -0.010 | 0.571*** |
|  | (0.045) | (0.044) | (0.136) |
| *Working class* | -0.199*** | -0.110** | 0.311** |
|  | (0.038) | (0.038) | (0.110) |
| *NA* | -0.103** | -0.053 | 0.395** |
|  | (0.037) | (0.036) | (0.122) |
| Subjective Social Status |  | 0.151*** | 0.214*** |
|  |  | (0.010) | (0.018) |
| Social Class * SSS |  |  |  |
| *Lower-grade professionals * SSS* |  |  | -0.029 |
|  |  |  | (0.018) |
| *Intermediate * SSS* |  |  | -0.050** |
|  |  |  | (0.019) |
| *Self-employed * SSS* |  |  | -0.104*** |
|  |  |  | (0.025) |
| *Working class * SSS* |  |  | -0.076*** |
|  |  |  | (0.019) |
| *NA * SSS* |  |  | -0.080*** |
|  |  |  | (0.022) |
| Household Income (ref. High) |  |  |  |
| *Low* | -0.310*** | -0.151*** | -0.149*** |
|  | (0.037) | (0.031) | (0.030) |
| *Middle* | -0.197*** | -0.104*** | -0.099*** |
|  | (0.023) | (0.019) | (0.019) |
| *NA* | -0.174*** | -0.116** | -0.112** |
|  | (0.038) | (0.036) | (0.036) |
| Education (ref. Tertiary) |  |  |  |
| *Primary* | -0.374*** | -0.280*** | -0.275*** |
|  | (0.061) | (0.057) | (0.057) |
| *Secondary* | -0.241*** | -0.195*** | -0.183*** |
|  | (0.037) | (0.035) | (0.035) |
| *NA* | -0.271*** | -0.141* | -0.146* |
|  | (0.070) | (0.072) | (0.070) |
| Social Class of Origin (ref. Higher-grade professionals) |  |  |  |
| *Lower-grade professionals* | -0.051+ | -0.037 | -0.033 |
|  | (0.030) | (0.031) | (0.031) |
| *Intermediate* | -0.143*** | -0.121*** | -0.117*** |
|  | (0.035) | (0.034) | (0.034) |
| *Self-employed* | -0.085** | -0.050 | -0.047 |
|  | (0.033) | (0.032) | (0.032) |
| *Working class* | -0.221*** | -0.180*** | -0.176*** |
|  | (0.038) | (0.039) | (0.038) |
| *NA* | -0.112* | -0.073 | -0.069 |
|  | (0.046) | (0.047) | (0.046) |
| Age | -0.010*** | -0.010*** | -0.010*** |
|  | (0.001) | (0.001) | (0.001) |
| Female | -0.094*** | -0.082*** | -0.079*** |
|  | (0.019) | (0.018) | (0.018) |
|  |  |  |  |
| Constant | 1.374*** | 0.260** | -0.115 |
|  | (0.097) | (0.094) | (0.122) |
|  |  |  |  |
| **C** |  |  |  |
| Social Class (ref. Higher-grade professionals) |  |  |  |
| *Lower-grade professionals* | -0.101*** | -0.066** | 0.159 |
|  | (0.025) | (0.025) | (0.119) |
| *Intermediate* | -0.150*** | -0.088* | 0.155 |
|  | (0.038) | (0.038) | (0.113) |
| *Self-employed* | -0.021 | 0.021 | 0.571*** |
|  | (0.040) | (0.039) | (0.136) |
| *Working class* | -0.185*** | -0.099* | 0.311** |
|  | (0.038) | (0.039) | (0.110) |
| *NA* | -0.049 | -0.001 | 0.503*** |
|  | (0.033) | (0.033) | (0.132) |
| Subjective Social Status |  | 0.140*** | 0.195*** |
|  |  | (0.011) | (0.018) |
| Social Class * SSS |  |  |  |
| *Lower-grade professionals * SSS* |  |  | -0.029 |
|  |  |  | (0.018) |
| *Intermediate * SSS* |  |  | -0.032+ |
|  |  |  | (0.018) |
| *Self-employed * SSS* |  |  | -0.088*** |
|  |  |  | (0.023) |
| *Working class * SSS* |  |  | -0.063*** |
|  |  |  | (0.019) |
| *NA * SSS* |  |  | -0.080*** |
|  | -0.086 | 0.033 | 0.021 |
| Household Income (ref. High) | (0.058) | (0.059) | (0.058) |
| *Low* |  |  |  |
|  | -0.051+ | -0.037 | -0.033 |
| *Middle* | (0.030) | (0.031) | (0.031) |
|  | -0.143*** | -0.121*** | -0.117*** |
| *NA* | (0.035) | (0.034) | (0.034) |
|  | -0.085** | -0.050 | -0.047 |
| Education (ref. Tertiary) | (0.033) | (0.032) | (0.032) |
| *Primary* | -0.167*** | -0.126** | -0.122** |
|  | (0.039) | (0.039) | (0.039) |
| *Secondary* | -0.112* | -0.073 | -0.069 |
|  | (0.046) | (0.047) | (0.046) |
| *NA* | -0.006*** | -0.006*** | -0.006*** |
|  | (0.001) | (0.001) | (0.001) |
| Social Class of Origin (ref. Higher-grade professionals) | -0.019 | -0.007 | -0.008 |
| *Lower-grade professionals* | (0.017) | (0.017) | (0.017) |
|  |  |  |  |
| *Intermediate* |  |  |  |
|  |  |  |  |
| *Self-employed* |  |  |  |
|  |  |  |  |
| *Working class* |  |  |  |
|  |  |  |  |
| *NA* |  |  |  |
|  |  |  |  |
| Age |  |  |  |
|  |  |  |  |
| Female |  |  |  |
|  |  |  | (0.022) |
|  |  |  |  |
| Constant | -0.348*** | -1.392*** | -1.764*** |
|  | (0.080) | (0.095) | (0.116) |
|  |  |  |  |
| **D** |  |  |  |
| Social Class (ref. Higher-grade professionals) |  |  |  |
| *Lower-grade professionals* | -0.101*** | -0.066** | 0.220 |
|  | (0.025) | (0.025) | (0.135) |
| *Intermediate* | 0.005 | 0.057 | 0.155 |
|  | (0.056) | (0.056) | (0.113) |
| *Self-employed* | 0.160* | 0.198* | 0.571*** |
|  | (0.080) | (0.078) | (0.136) |
| *Working class* | 0.048 | 0.105 | 0.311** |
|  | (0.070) | (0.074) | (0.110) |
| *NA* | 0.183* | 0.229** | 0.768*** |
|  | (0.072) | (0.073) | (0.150) |
| Subjective Social Status |  |  |  |
|  | 0.137* | 0.144** | 0.148** |
| Social Class * SSS | (0.055) | (0.047) | (0.046) |
| *Lower-grade professionals * SSS* | -0.037 | -0.104*** | -0.099*** |
|  | (0.056) | (0.019) | (0.019) |
| *Intermediate * SSS* | 0.154* | 0.123* | 0.132* |
|  | (0.061) | (0.054) | (0.055) |
| *Self-employed * SSS* |  |  |  |
|  | 0.367*** | 0.406*** | 0.380*** |
| *Working class * SSS* | (0.081) | (0.085) | (0.084) |
|  | 0.118 | 0.138+ | 0.120+ |
| *NA * SSS* | (0.073) | (0.074) | (0.072) |
|  | 0.446*** | 0.497*** | 0.467*** |
| Household Income (ref. High) | (0.117) | (0.117) | (0.113) |
| *Low* |  |  |  |
|  | -0.051+ | -0.037 | -0.033 |
| *Middle* | (0.030) | (0.031) | (0.031) |
|  | -0.143*** | -0.121*** | -0.117*** |
| *NA* | (0.035) | (0.034) | (0.034) |
|  | -0.085** | -0.050 | -0.047 |
| Education (ref. Tertiary) | (0.033) | (0.032) | (0.032) |
| *Primary* | 0.084+ | 0.121** | 0.124** |
|  | (0.047) | (0.046) | (0.046) |
| *Secondary* | -0.112* | -0.073 | -0.069 |
|  | (0.046) | (0.047) | (0.046) |
| *NA* | -0.006*** | -0.006*** | -0.006*** |
|  | (0.002) | (0.002) | (0.002) |
| Social Class of Origin (ref. Higher-grade professionals) | -0.065+ | -0.058 | -0.058 |
| *Lower-grade professionals* | (0.037) | (0.037) | (0.037) |
|  |  | 0.058** | 0.087*** |
| *Intermediate* |  | (0.018) | (0.024) |
|  |  |  |  |
| *Self-employed* |  |  | -0.029 |
|  |  |  | (0.018) |
| *Working class* |  |  | 0.004 |
|  |  |  | (0.022) |
| *NA* |  |  | -0.040 |
|  |  |  | (0.025) |
| Age |  |  | -0.012 |
|  |  |  | (0.022) |
| Female |  |  | -0.080*** |
|  |  |  | (0.022) |
|  |  |  |  |
| Constant | -4.516*** | -4.920*** | -5.186*** |
|  | (0.113) | (0.165) | (0.188) |
|  |  |  |  |
| Country-Wave Fixed Effects | *Yes* | *Yes* | *Yes* |
| BIC | 258527 | 256927 | 256935 |
| AIC | 257966 | 256366 | 256297 |

*Note*: Data from ISSP Research Group (2024). N=100,004. + p<0.10, * p<0.05, ** p<0.01, *** p<0.001

Table A14. Mediation Analyses of the Relationship Between Social Class and Subjective Social Status via Perceived Social Structure and Between Social Class and Perceived Social Structure via Subjective Social Status.

| *Model Type* | Linear Regression Model | Linear Regression Model | Linear Regression Model | Logit Regression Model |
| --- | --- | --- | --- | --- |
| *Variable of Interest (X)* | Social Class | Social Class | Social Class | Social Class |
| *Outcome Variable (Y)* | Subjective Social Status | Perceived Social Structure | Subjective Social Status | Perceived Social Structure (A vs BCDE) |
| *Mediator Variable (Z)* | Perceived Social Structure | Subjective Social Status | Perceived Social Structure (A vs BCDE) | Subjective Social Status |
| **Social Class** |  |  |  |  |
| Higher grade professionals (ref.) |  |  |  |  |
| Lower grade professionals |  |  |  |  |
| *Reduced* | -0.243*** | -0.045*** | -0.243*** | 0.052 |
|  | (0.021) | (0.013) | (0.021) | (0.043) |
| *Full* | -0.235*** | -0.025+ | -0.241*** | 0.010 |
|  | (0.021) | (0.013) | (0.021) | (0.043) |
| *Difference* | -0.008 | -0.020*** | -0.002 | 0.042*** |
|  | (0.007) | (0.005) | (0.005) | (0.011) |
| Intermediate occupations |  |  |  |  |
| *Reduced* | -0.454*** | -0.082*** | -0.454*** | 0.143** |
|  | (0.026) | (0.016) | (0.027) | (0.044) |
| *Full* | -0.437*** | -0.044** | -0.447*** | 0.064 |
|  | (0.026) | (0.016) | (0.027) | (0.044) |
| *Difference* | -0.016* | -0.037*** | -0.007 | 0.078*** |
|  | (0.007) | (0.006) | (0.005) | (0.012) |
| Self-Employed |  |  |  |  |
| *Reduced* | -0.311*** | -0.023 | -0.311*** | 0.060 |
|  | (0.043) | (0.020) | (0.044) | (0.057) |
| *Full* | -0.305*** | 0.003 | -0.309*** | 0.006 |
|  | (0.043) | (0.020) | (0.044) | (0.057) |
| *Difference* | -0.006 | -0.026*** | -0.002 | 0.054*** |
|  | (0.007) | (0.005) | (0.005) | (0.012) |
| Working Class |  |  |  |  |
| *Reduced* | -0.658*** | -0.095*** | -0.658*** | 0.182*** |
|  | (0.026) | (0.019) | (0.026) | (0.054) |
| *Full* | -0.637*** | -0.041* | -0.647*** | 0.068 |
|  | (0.026) | (0.019) | (0.026) | (0.054) |
| *Difference* | -0.021** | -0.054*** | -0.010+ | 0.114*** |
|  | (0.007) | (0.006) | (0.005) | (0.014) |
| **Counfounding Percentages (%)** |  |  |  |  |
| Higher grade professionals (ref.) |  |  |  |  |
| Lower grade professionals | 3.41 | 45.01 | 0.71 | 80.14 |
| Intermediate occupations | 3.36 | 45.85 | 1.45 | 55.02 |
| Self-Employed | 1.93 | 112.64 | 0.55 | 89.79 |
| Working class | 3.14 | 57.05 | 1.57 | 62.50 |

*Note*: Linear and logistic regression models estimated with the KHB decomposition technique (Karlson et al., 2012). The reduced model includes only social class as predictor; the full model adds the mediator (either subjective social status or perceived social structure). The “difference” represents the indirect effect through the mediator. Coefficients are shown for each social class relative to higher-grade professionals. Confounding percentages report the share of the total effect explained by mediation. Standard errors in parentheses. Models control for household income terciles, education, social class background, age, gender, survey year, and country–wave fixed effects. Weighted ISSP data (1992, 1999, 2009, 2019). N=100,004. + p<0.10, * p<0.05, ** p<0.01, *** p<0.001

Table A15. Results of Linear Regression Models of Subjective Social Status as a Function of Social Class and Perceived Social Structure.

|  | (1) | (2) | (3) |
| --- | --- | --- | --- |
|  | M1 | M2 | M3 |
| Social Class (ref. Higher-grade professionals) |  |  |  |
| *Lower-grade professionals* | -0.237*** | -0.228*** | -0.243*** |
|  | (0.021) | (0.021) | (0.039) |
| *Intermediate* | -0.436*** | -0.419*** | -0.427*** |
|  | (0.028) | (0.028) | (0.044) |
| *Self-employed* | -0.284*** | -0.279*** | -0.340*** |
|  | (0.043) | (0.042) | (0.076) |
| *Working class* | -0.623*** | -0.602*** | -0.596*** |
|  | (0.027) | (0.026) | (0.044) |
| *NA* | -0.380*** | -0.377*** | -0.365*** |
|  | (0.040) | (0.039) | (0.048) |
| Household Income (ref. High) |  |  |  |
| *Low* | -1.020*** | -0.988*** | -0.988*** |
|  | (0.045) | (0.042) | (0.042) |
| *Middle* | -0.541*** | -0.523*** | -0.523*** |
|  | (0.029) | (0.028) | (0.028) |
| *NA* | -0.351*** | -0.331*** | -0.332*** |
|  | (0.037) | (0.038) | (0.037) |
| Education (ref. Tertiary) |  |  |  |
| *Primary* | -0.665*** | -0.634*** | -0.634*** |
|  | (0.034) | (0.032) | (0.032) |
| *Secondary* | -0.325*** | -0.310*** | -0.311*** |
|  | (0.023) | (0.022) | (0.022) |
| *NA* | -0.940*** | -0.922*** | -0.920*** |
|  | (0.071) | (0.075) | (0.074) |
| Social Class of Origin (ref. Higher-grade professionals) |  |  |  |
| *Lower-grade professionals* | -0.133*** | -0.126*** | -0.126*** |
|  | (0.028) | (0.028) | (0.028) |
| *Intermediate* | -0.199*** | -0.184*** | -0.184*** |
|  | (0.030) | (0.029) | (0.029) |
| *Self-employed* | -0.272*** | -0.263*** | -0.262*** |
|  | (0.035) | (0.034) | (0.034) |
| *Working class* | -0.336*** | -0.315*** | -0.315*** |
|  | (0.037) | (0.037) | (0.037) |
| *NA* | -0.283*** | -0.271*** | -0.272*** |
|  | (0.041) | (0.041) | (0.041) |
| Age | -0.002+ | -0.002 | -0.002 |
|  | (0.001) | (0.001) | (0.001) |
| Female | -0.080*** | -0.077*** | -0.077*** |
|  | (0.018) | (0.018) | (0.018) |
| Perceived Social Structure (ref. B) |  |  |  |
| *A* |  | -0.314*** | -0.360*** |
|  |  | (0.025) | (0.073) |
| *C* |  | 0.175*** | 0.176*** |
|  |  | (0.020) | (0.043) |
| *D* |  | 0.337*** | 0.341*** |
|  |  | (0.023) | (0.043) |
| *E* |  | 0.132* | 0.184 |
|  |  | (0.055) | (0.139) |
| Perceived Social Structure * Social Class |  |  |  |
| *A * Lower-grade professionals* |  |  | 0.072 |
|  |  |  | (0.074) |
| *A * Intermediate* |  |  | 0.075 |
|  |  |  | (0.077) |
| *A* Self-employed* |  |  | 0.177 |
|  |  |  | (0.135) |
| *A * Working class* |  |  | 0.013 |
|  |  |  | (0.080) |
| *A * NA* |  |  | 0.006 |
|  |  |  | (0.096) |
| *C * Lower-grade professionals* |  |  | 0.031 |
|  |  |  | (0.050) |
| *C * Intermediate* |  |  | -0.025 |
|  |  |  | (0.059) |
| *C * Self-employed* |  |  | 0.071 |
|  |  |  | (0.074) |
| *C * Working class* |  |  | -0.033 |
|  |  |  | (0.054) |
| *C * NA* |  |  | -0.006 |
|  |  |  | (0.060) |
| *D * Lower-grade professionals* |  |  | -0.020 |
|  |  |  | (0.051) |
| *D * Intermediate* |  |  | -0.022 |
|  |  |  | (0.056) |
| *D * Self-employed* |  |  | 0.033 |
|  |  |  | (0.072) |
| *D * Working class* |  |  | 0.013 |
|  |  |  | (0.058) |
| *D * NA* |  |  | -0.005 |
|  |  |  | (0.062) |
| *E * Lower-grade professionals* |  |  | -0.036 |
|  |  |  | (0.149) |
| *E * Intermediate* |  |  | 0.077 |
|  |  |  | (0.150) |
| *E * Self-employed* |  |  | -0.015 |
|  |  |  | (0.161) |
| *E * Working class* |  |  | -0.012 |
|  |  |  | (0.154) |
| *E * NA* |  |  | -0.216 |
|  |  |  | (0.179) |
|  |  |  |  |
|  |  |  |  |
| Constant | 6.753*** | 6.642*** | 6.647*** |
|  | (0.055) | (0.056) | (0.057) |
|  |  |  |  |
| Country-Wave Fixed Effect | *Yes* | *Yes* | *Yes* |
| BIC | 371612 | 369955 | 370149 |
| AIC | 371441 | 369746 | 369750 |

*Note*: Data from ISSP Research Group (2024). N=100,004. + p<0.10, * p<0.05, ** p<0.01, *** p<0.001

Figure A4. Predicted Subjective Social Status by Social Class and Perceived Inequality Structure.
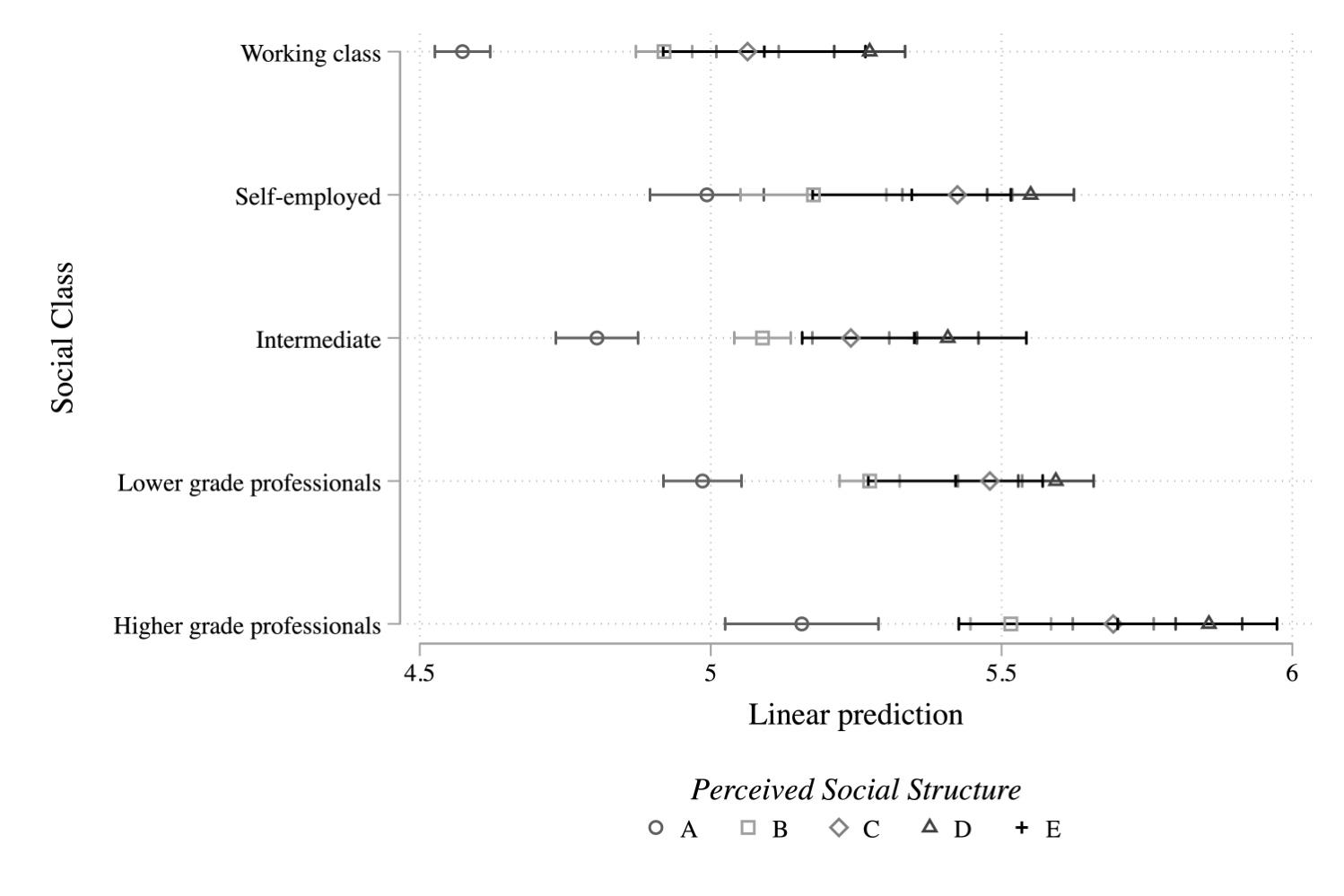


*Note*: Points show predicted values of subjective social status (1–10) for each perceived inequality structure type (A–E), separately by social class. Estimates are from linear regression models including social class as predictor and perceived inequality structure as mediator. Horizontal bars represent 95% confidence intervals. Results are based on Model 3 in Table A15. Weighted ISSP data (1992, 1999, 2009, 2019).

Figure A5. Predicted Probability of Perceiving Type A (Most Unequal) Social Structure by Social Class and Subjective Social Status, Logit Specification.


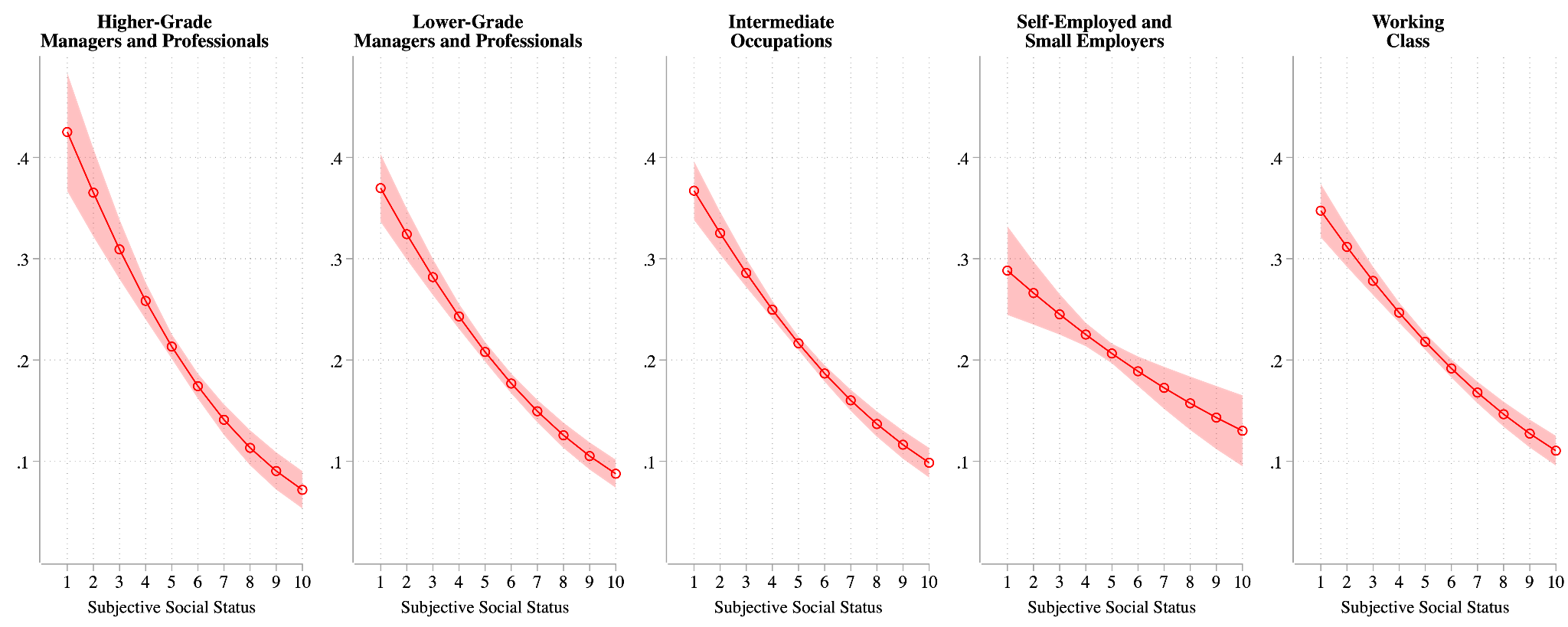


*Note*: Lines show predicted probabilities of selecting Type A (see Figure 1) across levels of subjective social status (1–10), separately for each social class. Shaded areas represent 95% confidence intervals. Estimates are from a Generalized Ordered Logit model controlling for household income, education, social class of origin, age, gender, survey year, and country–wave fixed effects. Predictions are based on Model 3 in Table A15. Weighted ISSP data (1992, 1999, 2009, 2019); N = 100,004.

Table A16. Results of Logit Models Predicting the Probability of Selecting Type A (Most Unequal) Social Structure by Social Class and Subjective Social Status.

|  | (1) | (2) | (3) |
| --- | --- | --- | --- |
|  | Social Class  (Log Odds) | SSS and Social Class  (Log Odds) | Interaction Social Class * SSS  (Log Odds) |
| Social Class (ref. Higher-grade professionals) |  |  |  |
| *Lower-grade professionals* | 0.054 | 0.018 | -0.281+ |
|  | (0.043) | (0.044) | (0.162) |
| *Intermediate* | 0.148*** | 0.082+ | -0.308+ |
|  | (0.043) | (0.045) | (0.166) |
| *Self-employed* | 0.058 | 0.012 | -0.742*** |
|  | (0.055) | (0.056) | (0.211) |
| *Working class* | 0.184*** | 0.087 | -0.418* |
|  | (0.053) | (0.054) | (0.164) |
| *NA* | -0.008 | -0.073 | -0.635*** |
|  | (0.046) | (0.046) | (0.156) |
| Household Income (ref. High) |  |  |  |
| *Low* | 0.317*** | 0.154*** | 0.152*** |
|  | (0.038) | (0.033) | (0.032) |
| *Middle* | 0.178*** | 0.091** | 0.086** |
|  | (0.032) | (0.030) | (0.030) |
| *NA* | 0.182*** | 0.133** | 0.129* |
|  | (0.050) | (0.051) | (0.052) |
| Education (ref. Tertiary) |  |  |  |
| *Primary* | 0.218*** | 0.105* | 0.103* |
|  | (0.050) | (0.048) | (0.048) |
| *Secondary* | 0.095* | 0.039 | 0.030 |
|  | (0.037) | (0.036) | (0.035) |
| *NA* | 0.102 | -0.068 | -0.058 |
|  | (0.075) | (0.080) | (0.081) |
| Social Class of Origin (ref. Higher-grade professionals) |  |  |  |
| *Lower-grade professionals* | 0.037 | 0.013 | 0.009 |
|  | (0.046) | (0.047) | (0.046) |
| *Intermediate* | 0.089+ | 0.058 | 0.054 |
|  | (0.049) | (0.048) | (0.048) |
| *Self-employed* | 0.011 | -0.034 | -0.036 |
|  | (0.045) | (0.045) | (0.045) |
| *Working class* | 0.113* | 0.060 | 0.056 |
|  | (0.050) | (0.051) | (0.050) |
| *NA* | 0.029 | -0.017 | -0.020 |
|  | (0.065) | (0.066) | (0.065) |
| Age | 0.004*** | 0.004*** | 0.004*** |
|  | (0.001) | (0.001) | (0.001) |
| Female | -0.004 | -0.018 | -0.020 |
|  | (0.023) | (0.023) | (0.023) |
| Subjective Social Status |  | -0.166*** | -0.251*** |
|  |  | (0.012) | (0.028) |
| Social Class * SSS |  |  |  |
| *Lower-grade professionals * SSS* |  |  | 0.050+ |
|  |  |  | (0.030) |
| *Intermediate * SSS* |  |  | 0.065* |
|  |  |  | (0.030) |
| *Self-employed * SSS* |  |  | 0.140*** |
|  |  |  | (0.042) |
| *Working class * SSS* |  |  | 0.089** |
|  |  |  | (0.031) |
| *NA * SSS* |  |  | 0.101** |
|  |  |  | (0.032) |
|  |  |  |  |
|  |  |  |  |
| Constant | -2.538*** | -1.296*** | -0.793*** |
|  | (0.098) | (0.129) | (0.147) |
|  |  |  |  |
| Country-Wave Fixed Effects | *Yes* | *Yes* | *Yes* |
| BIC | 97181 | 96074 | 96083 |
| AIC | 96981 | 95865 | 95826 |

*Note*: Data from ISSP Research Group (2024). N=100,004. + p<0.10, * p<0.05, ** p<0.01, *** p<0.001

Figure A6. Predicted Probability of Perceiving Type A (Most Unequal) Social Structure by Social Class and Subjective Social Status, Logit Specification.


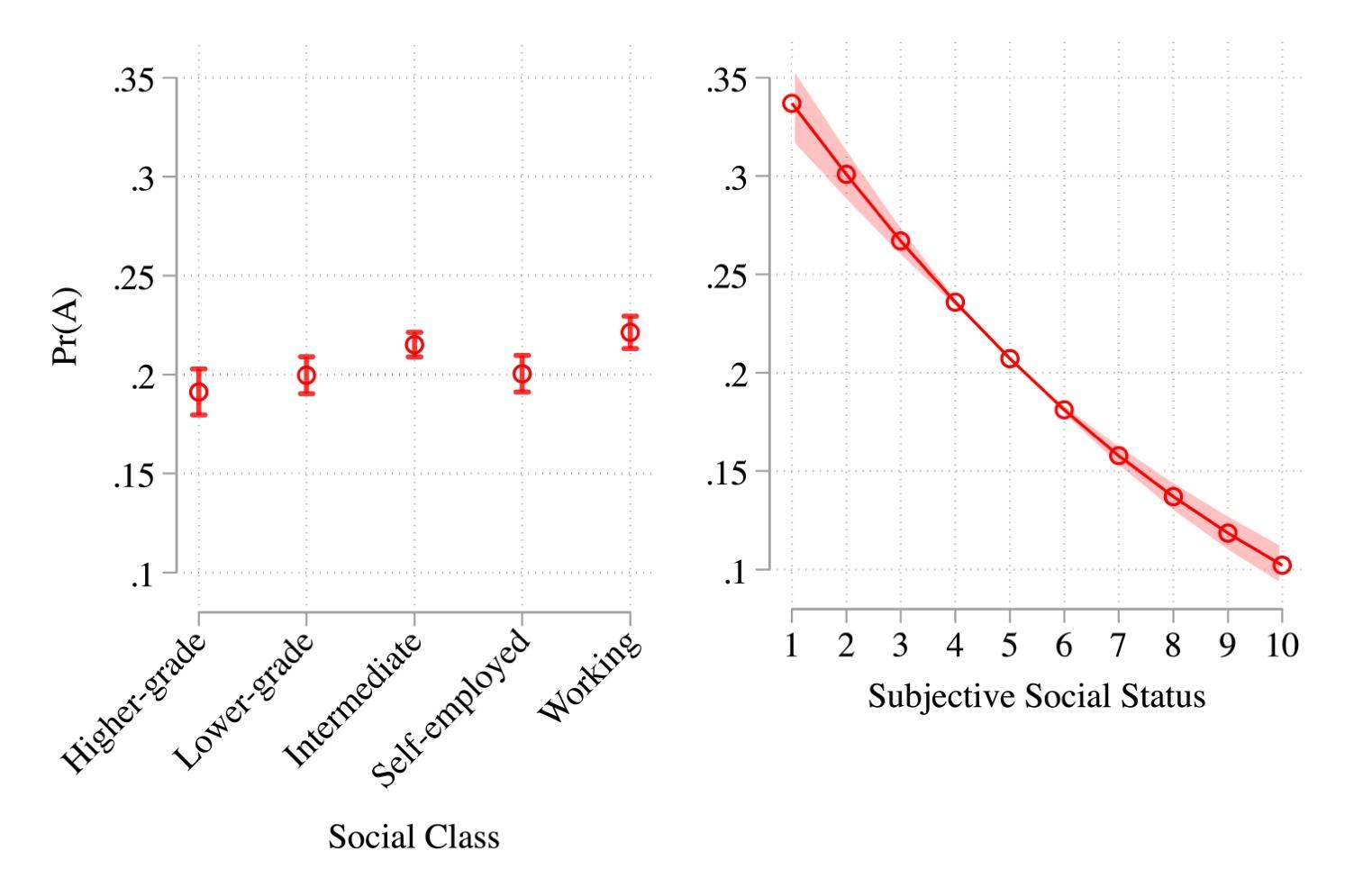


*Note*: Panel A shows predicted probabilities of selecting Type A (most unequal structure) across social classes. Panel B shows the same probabilities across levels of subjective social status (1–10). Shaded areas represent 95% confidence intervals. Estimates are from logit models including social class and subjective social status as predictors, with perceived social structure as outcome. Results are based on Models 1 and 2 in Table A16. Weighted ISSP data (1992, 1999, 2009, 2019).

Table A17. Results of Generalised Ordered Logit Models for Ordinal Dependent Variables for Perceived Social Structure, with interaction between social class and education.

|  | (1) | (2) | (3) |
| --- | --- | --- | --- |
|  | Social Class | SSS and Social Class | Interaction Social Class * SSS |
|  |  |  |  |
| A |  |  |  |
| Social Class (ref. Higher-grade professionals) |  |  |  |
| *Lower-grade professionals* | -0.073+ | -0.030 | 0.171+ |
|  | (0.040) | (0.040) | (0.102) |
| *Intermediate* | -0.163** | -0.057 | 0.205+ |
|  | (0.052) | (0.048) | (0.112) |
| *Self-employed* | -0.069 | -0.013 | 0.568*** |
|  | (0.080) | (0.079) | (0.131) |
| *Working class* | -0.206** | -0.083 | 0.374** |
|  | (0.065) | (0.060) | (0.118) |
| *NA* | 0.002 | 0.029 | 0.468*** |
|  | (0.080) | (0.072) | (0.103) |
| Education (ref. Tertiary) |  |  |  |
| *Primary* | -0.308** | -0.216* | -0.095 |
|  | (0.105) | (0.109) | (0.100) |
| *Secondary* | -0.063 | 0.016 | -0.009 |
|  | (0.073) | (0.072) | (0.055) |
| *NA* | -0.137 | -0.058 | -0.070 |
|  | (0.157) | (0.155) | (0.188) |
| Social Class * Education |  |  |  |
| *Lower-grade professionals * Primary* | 0.057 | 0.067 | -0.069 |
|  | (0.108) | (0.108) | (0.094) |
| *Lower-grade professionals * Secondary* | -0.057 | -0.081 | -0.047 |
|  | (0.070) | (0.069) | (0.056) |
| *Lower-grade professionals * NA* | 0.873* | 0.829* | 0.617 |
|  | (0.365) | (0.364) | (0.381) |
| *Intermediate * Primary* | 0.022 | 0.021 | -0.103 |
|  | (0.097) | (0.101) | (0.091) |
| *Intermediate * Secondary* | -0.031 | -0.068 | -0.044 |
|  | (0.067) | (0.067) | (0.057) |
| *Intermediate * NA* | -0.105 | -0.084 | -0.042 |
|  | (0.238) | (0.239) | (0.270) |
| *Self-employed * Primary* | 0.071 | 0.094 | -0.057 |
|  | (0.119) | (0.123) | (0.108) |
| *Self-employed * Secondary* | -0.043 | -0.079 | -0.076 |
|  | (0.100) | (0.100) | (0.085) |
| *Self-employed * NA* | 0.050 | 0.149 | 0.086 |
|  | (0.198) | (0.189) | (0.207) |
| *Working class * Primary* | 0.089 | 0.091 | -0.058 |
|  | (0.104) | (0.107) | (0.095) |
| *Working class * Secondary* | -0.055 | -0.097 | -0.095 |
|  | (0.071) | (0.071) | (0.062) |
| *Working class * NA* | -0.091 | -0.025 | -0.037 |
|  | (0.204) | (0.196) | (0.198) |
| *NA * Primary* | 0.046 | 0.106 | -0.010 |
|  | (0.122) | (0.124) | (0.114) |
| *NA * Secondary* | -0.056 | -0.058 | -0.019 |
|  | (0.086) | (0.083) | (0.077) |
| *NA * NA* | 0.027 | 0.155 | 0.131 |
|  | (0.211) | (0.201) | (0.192) |
| Household Income (ref. High) |  |  |  |
| *Low* | -0.335*** | -0.167*** | -0.165*** |
|  | (0.037) | (0.031) | (0.031) |
| *Middle* | -0.178*** | -0.096*** | -0.099*** |
|  | (0.023) | (0.021) | (0.019) |
| *NA* | -0.195*** | -0.146** | -0.128** |
|  | (0.052) | (0.053) | (0.039) |
| Social Class of Origin (ref. Higher-grade professionals) |  |  |  |
| *Lower-grade professionals* | -0.073* | -0.052 | -0.033 |
|  | (0.034) | (0.035) | (0.030) |
| *Intermediate* | -0.152*** | -0.124** | -0.116*** |
|  | (0.042) | (0.041) | (0.034) |
| *Self-employed* | -0.106** | -0.066+ | -0.047 |
|  | (0.037) | (0.036) | (0.032) |
| *Working class* | -0.188*** | -0.136*** | -0.136*** |
|  | (0.041) | (0.041) | (0.037) |
| *NA* | -0.115* | -0.072 | -0.068 |
|  | (0.049) | (0.050) | (0.046) |
| Age | -0.005*** | -0.004*** | -0.004*** |
|  | (0.001) | (0.001) | (0.001) |
| Female | -0.002 | 0.011 | 0.003 |
|  | (0.022) | (0.022) | (0.018) |
| Subjective Social Status |  | 0.169*** | 0.226*** |
|  |  | (0.011) | (0.018) |
| Social Class * SSS |  |  |  |
| *Lower-grade professionals * SSS* |  |  | -0.031+ |
|  |  |  | (0.018) |
| *Intermediate * SSS* |  |  | -0.045* |
|  |  |  | (0.021) |
| *Self-employed * SSS* |  |  | -0.097*** |
|  |  |  | (0.023) |
| *Working class * SSS* |  |  | -0.074*** |
|  |  |  | (0.021) |
| *NA * SSS* |  |  | -0.079*** |
|  |  |  | (0.021) |
|  |  |  |  |
| Constant | 2.672*** | 1.418*** | 1.049*** |
|  | (0.097) | (0.117) | (0.106) |
|  |  |  |  |
| B |  |  |  |
| Social Class (ref. Higher-grade professionals) |  |  |  |
| *Lower-grade professionals* | -0.073+ | -0.030 | 0.114 |
|  | (0.040) | (0.040) | (0.112) |
| *Intermediate* | -0.157** | -0.057 | 0.205+ |
|  | (0.053) | (0.048) | (0.112) |
| *Self-employed* | -0.209* | -0.157+ | 0.409** |
|  | (0.088) | (0.081) | (0.135) |
| *Working class* | -0.160** | -0.025 | 0.374** |
|  | (0.058) | (0.056) | (0.118) |
| *NA* | -0.047 | 0.029 | 0.382** |
|  | (0.077) | (0.072) | (0.119) |
| Education (ref. Tertiary) |  |  |  |
| *Primary* | -0.482*** | -0.417*** | -0.260* |
|  | (0.107) | (0.108) | (0.102) |
| *Secondary* | -0.210** | -0.147* | -0.147** |
|  | (0.066) | (0.064) | (0.055) |
| *NA* | -0.137 | -0.058 | -0.070 |
|  | (0.157) | (0.155) | (0.188) |
| Social Class * Education |  |  |  |
| *Lower-grade professionals * Primary* | 0.057 | 0.067 | -0.069 |
|  | (0.108) | (0.108) | (0.094) |
| *Lower-grade professionals * Secondary* | -0.057 | -0.081 | -0.047 |
|  | (0.070) | (0.069) | (0.056) |
| *Lower-grade professionals * NA* | -0.070 | -0.082 | -0.090 |
|  | (0.163) | (0.168) | (0.220) |
| *Intermediate * Primary* | 0.022 | 0.021 | -0.103 |
|  | (0.097) | (0.101) | (0.091) |
| *Intermediate * Secondary* | -0.031 | -0.068 | -0.044 |
|  | (0.067) | (0.067) | (0.057) |
| *Intermediate * NA* | -0.253 | -0.248 | -0.105 |
|  | (0.191) | (0.189) | (0.215) |
| *Self-employed * Primary* | 0.372** | 0.417** | 0.214+ |
|  | (0.131) | (0.128) | (0.119) |
| *Self-employed * Secondary* | 0.099 | 0.090 | 0.050 |
|  | (0.093) | (0.091) | (0.078) |
| *Self-employed * NA* | 0.050 | 0.149 | 0.086 |
|  | (0.198) | (0.189) | (0.207) |
| *Working class * Primary* | 0.089 | 0.091 | -0.058 |
|  | (0.104) | (0.107) | (0.095) |
| *Working class * Secondary* | -0.055 | -0.097 | -0.095 |
|  | (0.071) | (0.071) | (0.062) |
| *Working class * NA* | -0.250 | -0.220 | -0.202 |
|  | (0.201) | (0.195) | (0.200) |
| *NA * Primary* | 0.046 | 0.106 | -0.010 |
|  | (0.122) | (0.124) | (0.114) |
| *NA * Secondary* | -0.056 | -0.058 | -0.019 |
|  | (0.086) | (0.083) | (0.077) |
| *NA * NA* | -0.023 | 0.035 | 0.004 |
|  | (0.229) | (0.222) | (0.202) |
| Household Income (ref. High) |  |  |  |
| *Low* | -0.302*** | -0.151*** | -0.150*** |
|  | (0.036) | (0.031) | (0.030) |
| *Middle* | -0.178*** | -0.096*** | -0.099*** |
|  | (0.023) | (0.021) | (0.019) |
| *NA* | -0.186*** | -0.134*** | -0.111** |
|  | (0.035) | (0.035) | (0.036) |
| Social Class of Origin (ref. Higher-grade professionals) |  |  |  |
| *Lower-grade professionals* | -0.073* | -0.052 | -0.033 |
|  | (0.034) | (0.035) | (0.030) |
| *Intermediate* | -0.152*** | -0.124** | -0.116*** |
|  | (0.042) | (0.041) | (0.034) |
| *Self-employed* | -0.106** | -0.066+ | -0.047 |
|  | (0.037) | (0.036) | (0.032) |
| *Working class* | -0.233*** | -0.185*** | -0.174*** |
|  | (0.043) | (0.044) | (0.038) |
| *NA* | -0.115* | -0.072 | -0.068 |
|  | (0.049) | (0.050) | (0.046) |
| Age | -0.010*** | -0.010*** | -0.010*** |
|  | (0.001) | (0.001) | (0.001) |
| Female | -0.092*** | -0.083*** | -0.079*** |
|  | (0.019) | (0.019) | (0.018) |
| Subjective Social Status |  | 0.155*** | 0.214*** |
|  |  | (0.010) | (0.017) |
| Social Class * SSS |  |  |  |
| *Lower-grade professionals * SSS* |  |  | -0.031+ |
|  |  |  | (0.018) |
| *Intermediate * SSS* |  |  | -0.053** |
|  |  |  | (0.018) |
| *Self-employed * SSS* |  |  | -0.097*** |
|  |  |  | (0.023) |
| *Working class * SSS* |  |  | -0.077*** |
|  |  |  | (0.018) |
| *NA * SSS* |  |  | -0.079*** |
|  |  |  | (0.021) |
|  |  |  |  |
| Constant | 1.397*** | 0.232* | -0.123 |
|  | (0.106) | (0.106) | (0.120) |
|  |  |  |  |
| C |  |  |  |
| Social Class (ref. Higher-grade professionals) |  |  |  |
| *Lower-grade professionals* | -0.073+ | -0.030 | 0.179 |
|  | (0.040) | (0.040) | (0.119) |
| *Intermediate* | -0.117* | -0.057 | 0.205+ |
|  | (0.051) | (0.048) | (0.112) |
| *Self-employed* | -0.024 | 0.002 | 0.607*** |
|  | (0.075) | (0.071) | (0.141) |
| *Working class* | -0.152* | -0.050 | 0.374** |
|  | (0.059) | (0.056) | (0.118) |
| *NA* | -0.000 | 0.029 | 0.486*** |
|  | (0.073) | (0.072) | (0.130) |
| Education (ref. Tertiary) |  |  |  |
| *Primary* | -0.210* | -0.134 | -0.032 |
|  | (0.105) | (0.106) | (0.094) |
| *Secondary* | -0.015 | 0.057 | 0.027 |
|  | (0.062) | (0.060) | (0.049) |
| *NA* | -0.137 | -0.058 | -0.070 |
|  | (0.157) | (0.155) | (0.188) |
| Social Class * Education |  |  |  |
| *Lower-grade professionals * Primary* | 0.057 | 0.067 | -0.069 |
|  | (0.108) | (0.108) | (0.094) |
| *Lower-grade professionals * Secondary* | -0.057 | -0.081 | -0.047 |
|  | (0.070) | (0.069) | (0.056) |
| *Lower-grade professionals * NA* | 0.500** | 0.481* | 0.440* |
|  | (0.190) | (0.187) | (0.213) |
| *Intermediate * Primary* | 0.022 | 0.021 | -0.103 |
|  | (0.097) | (0.101) | (0.091) |
| *Intermediate * Secondary* | -0.031 | -0.068 | -0.044 |
|  | (0.067) | (0.067) | (0.057) |
| *Intermediate * NA* | 0.063 | 0.081 | 0.191 |
|  | (0.211) | (0.211) | (0.233) |
| *Self-employed * Primary* | 0.174 | 0.208 | 0.061 |
|  | (0.130) | (0.128) | (0.125) |
| *Self-employed * Secondary* | -0.066 | -0.078 | -0.052 |
|  | (0.085) | (0.083) | (0.077) |
| *Self-employed * NA* | 0.050 | 0.149 | 0.086 |
|  | (0.198) | (0.189) | (0.207) |
| *Working class * Primary* | 0.089 | 0.091 | -0.058 |
|  | (0.104) | (0.107) | (0.095) |
| *Working class * Secondary* | -0.055 | -0.097 | -0.095 |
|  | (0.071) | (0.071) | (0.062) |
| *Working class * NA* | -0.111 | -0.079 | -0.076 |
|  | (0.200) | (0.195) | (0.204) |
| *NA * Primary* | 0.046 | 0.106 | -0.010 |
|  | (0.122) | (0.124) | (0.114) |
| *NA * Secondary* | -0.056 | -0.058 | -0.019 |
|  | (0.086) | (0.083) | (0.077) |
| *NA * NA* | 0.218 | 0.282 | 0.177 |
|  | (0.231) | (0.224) | (0.205) |
| Household Income (ref. High) |  |  |  |
| *Low* | -0.227*** | -0.082** | -0.096*** |
|  | (0.033) | (0.030) | (0.029) |
| *Middle* | -0.178*** | -0.096*** | -0.099*** |
|  | (0.023) | (0.021) | (0.019) |
| *NA* | -0.114*** | -0.060+ | -0.054+ |
|  | (0.034) | (0.034) | (0.030) |
| Social Class of Origin (ref. Higher-grade professionals) |  |  |  |
| *Lower-grade professionals* | -0.073* | -0.052 | -0.033 |
|  | (0.034) | (0.035) | (0.030) |
| *Intermediate* | -0.152*** | -0.124** | -0.116*** |
|  | (0.042) | (0.041) | (0.034) |
| *Self-employed* | -0.106** | -0.066+ | -0.047 |
|  | (0.037) | (0.036) | (0.032) |
| *Working class* | -0.170*** | -0.121** | -0.121** |
|  | (0.044) | (0.045) | (0.039) |
| *NA* | -0.115* | -0.072 | -0.068 |
|  | (0.049) | (0.050) | (0.046) |
| Age | -0.007*** | -0.007*** | -0.006*** |
|  | (0.001) | (0.001) | (0.001) |
| Female | -0.021 | -0.010 | -0.007 |
|  | (0.019) | (0.018) | (0.017) |
| Subjective Social Status |  | 0.145*** | 0.197*** |
|  |  | (0.010) | (0.018) |
| Social Class * SSS |  |  |  |
| *Lower-grade professionals * SSS* |  |  | -0.031+ |
|  |  |  | (0.018) |
| *Intermediate * SSS* |  |  | -0.036* |
|  |  |  | (0.017) |
| *Self-employed * SSS* |  |  | -0.097*** |
|  |  |  | (0.023) |
| *Working class * SSS* |  |  | -0.065*** |
|  |  |  | (0.018) |
| *NA * SSS* |  |  | -0.079*** |
|  |  |  | (0.021) |
|  |  |  |  |
| Constant | -0.345*** | -1.433*** | -1.789*** |
|  | (0.091) | (0.104) | (0.115) |
|  |  |  |  |
| D |  |  |  |
| Social Class (ref. Higher-grade professionals) |  |  |  |
| *Lower-grade professionals* | -0.073+ | -0.030 | 0.235+ |
|  | (0.040) | (0.040) | (0.135) |
| *Intermediate* | 0.014 | -0.057 | 0.205+ |
|  | (0.075) | (0.048) | (0.112) |
| *Self-employed* | 0.330* | 0.268* | 1.032*** |
|  | (0.135) | (0.126) | (0.175) |
| *Working class* | 0.070 | 0.031 | 0.374** |
|  | (0.094) | (0.078) | (0.118) |
| *NA* | 0.172+ | 0.029 | 0.719*** |
|  | (0.092) | (0.072) | (0.143) |
| Education (ref. Tertiary) |  |  |  |
| *Primary* | 0.361** | 0.414** | 0.490*** |
|  | (0.134) | (0.135) | (0.115) |
| *Secondary* | 0.167+ | 0.240* | 0.180* |
|  | (0.095) | (0.094) | (0.085) |
| *NA* | -0.137 | -0.058 | -0.070 |
|  | (0.157) | (0.155) | (0.188) |
| Social Class * Education |  |  |  |
| *Lower-grade professionals * Primary* | 0.057 | 0.067 | -0.069 |
|  | (0.108) | (0.108) | (0.094) |
| *Lower-grade professionals * Secondary* | -0.057 | -0.081 | -0.047 |
|  | (0.070) | (0.069) | (0.056) |
| *Lower-grade professionals * NA* | -0.446 | -0.594 | -0.147 |
|  | (0.876) | (0.877) | (0.805) |
| *Intermediate * Primary* | 0.022 | 0.021 | -0.103 |
|  | (0.097) | (0.101) | (0.091) |
| *Intermediate * Secondary* | -0.031 | -0.068 | -0.044 |
|  | (0.067) | (0.067) | (0.057) |
| *Intermediate * NA* | 0.863** | 0.883** | 0.949** |
|  | (0.296) | (0.304) | (0.353) |
| *Self-employed * Primary* | -0.212 | -0.202 | -0.358* |
|  | (0.184) | (0.182) | (0.159) |
| *Self-employed * Secondary* | -0.074 | -0.112 | -0.062 |
|  | (0.135) | (0.131) | (0.129) |
| *Self-employed * NA* | 0.050 | 0.149 | 0.086 |
|  | (0.198) | (0.189) | (0.207) |
| *Working class * Primary* | 0.089 | 0.091 | -0.058 |
|  | (0.104) | (0.107) | (0.095) |
| *Working class * Secondary* | -0.055 | -0.097 | -0.095 |
|  | (0.071) | (0.071) | (0.062) |
| *Working class * NA* | 0.705** | 0.664** | 0.615* |
|  | (0.250) | (0.249) | (0.281) |
| *NA * Primary* | 0.046 | 0.106 | -0.010 |
|  | (0.122) | (0.124) | (0.114) |
| *NA * Secondary* | -0.056 | -0.058 | -0.019 |
|  | (0.086) | (0.083) | (0.077) |
| *NA * NA* | 0.750** | 0.816** | 0.709** |
|  | (0.264) | (0.252) | (0.248) |
| Household Income (ref. High) |  |  |  |
| *Low* | 0.072 | 0.165*** | 0.149** |
|  | (0.046) | (0.048) | (0.047) |
| *Middle* | -0.178*** | -0.096*** | -0.099*** |
|  | (0.023) | (0.021) | (0.019) |
| *NA* | 0.073 | 0.122+ | 0.127* |
|  | (0.074) | (0.072) | (0.054) |
| Social Class of Origin (ref. Higher-grade professionals) |  |  |  |
| *Lower-grade professionals* | -0.073* | -0.052 | -0.033 |
|  | (0.034) | (0.035) | (0.030) |
| *Intermediate* | -0.152*** | -0.124** | -0.116*** |
|  | (0.042) | (0.041) | (0.034) |
| *Self-employed* | -0.106** | -0.066+ | -0.047 |
|  | (0.037) | (0.036) | (0.032) |
| *Working class* | 0.045 | 0.083 | 0.118* |
|  | (0.054) | (0.054) | (0.047) |
| *NA* | -0.115* | -0.072 | -0.068 |
|  | (0.049) | (0.050) | (0.046) |
| Age | -0.006*** | -0.007*** | -0.007*** |
|  | (0.002) | (0.002) | (0.002) |
| Female | -0.070+ | -0.062+ | -0.059 |
|  | (0.036) | (0.035) | (0.037) |
| Subjective Social Status |  | 0.059** | 0.101*** |
|  |  | (0.019) | (0.025) |
| Social Class * SSS |  |  |  |
| *Lower-grade professionals * SSS* |  |  | -0.031+ |
|  |  |  | (0.018) |
| *Intermediate * SSS* |  |  | -0.007 |
|  |  |  | (0.022) |
| *Self-employed * SSS* |  |  | -0.097*** |
|  |  |  | (0.023) |
| *Working class * SSS* |  |  | -0.024 |
|  |  |  | (0.022) |
| *NA * SSS* |  |  | -0.079*** |
|  |  |  | (0.021) |
|  |  |  |  |
| Constant | -4.450*** | -4.858*** | -5.274*** |
|  | (0.107) | (0.171) | (0.191) |
|  |  |  |  |
| Observations | 100004 | 100004 | 100004 |
| BIC | 259718 | 257987 | 257204 |
| AIC | 258909 | 257197 | 256291 |

*Note*: Data from ISSP Research Group (2024), weighted. N=100,004. + p<0.10, * p<0.05, ** p<0.01, *** p<0.001
